# Catalytic Formal Hydroamination of Allylic Alcohols Using Manganese PNP‐Pincer Complexes

**DOI:** 10.1002/adsc.202100081

**Published:** 2021-03-17

**Authors:** Leandro Duarte de Almeida, Florian Bourriquen, Kathrin Junge, Matthias Beller

**Affiliations:** ^1^ Leibniz-Institut für Katalyse e.V. Albert-Einstein-Str. 29a 18059 Rostock Germany

**Keywords:** hydroamination, allylic alcohol, manganese, amine, pincer complex

## Abstract

Several manganese‐PNP pincer catalysts for the formal hydroamination of allylic alcohols are presented. The resulting γ‐amino alcohols are selectively obtained in high yields applying **Mn‐1** in a tandem process under mild conditions.

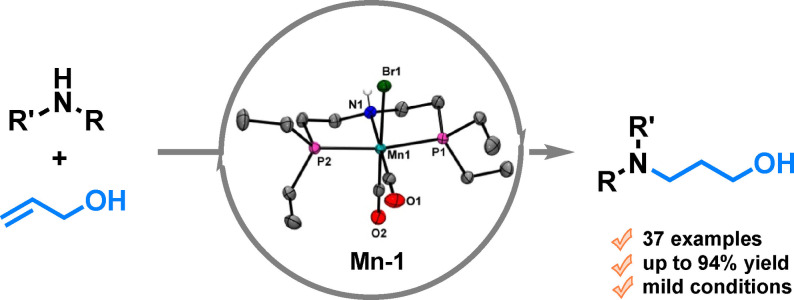

Nitrogen containing compounds, including γ‐amino alcohols, show many interesting biological properties and thus find numerous applications as agrochemicals and pharmaceuticals (Scheme 1a). In general, the formation of C−N bonds continues to be a relevant topic in many organic syntheses and has numerous implementations in the chemical industry.^[1]^ Among the various methodologies known in this area, direct hydroamination reactions are particularly appealing as they are in line with the green chemistry principles due to the 100% atom efficiency and the good availability of substrates.^[2]^ In the past, a plethora of different catalyst systems and metals have been utilized for olefin hydroamination as outlined in several excellent reviews.^[3]^

**Scheme 1:**
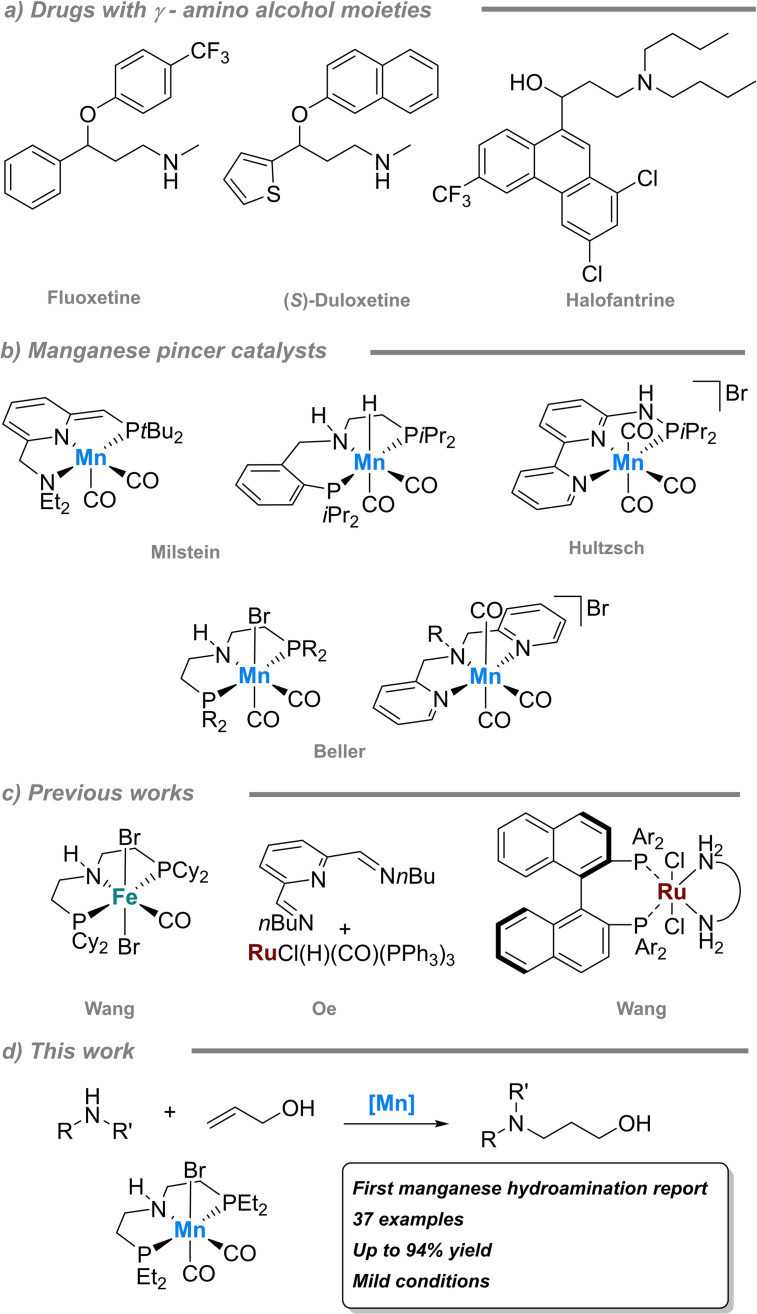
Importance of γ‐amino alcohols and catalyst systems for carbon‐nitrogen bond formation.

Due to the significance of C−N bond formations, many research groups have focussed on the development of new methodologies in this field, especially in recent years using earth‐abundant 3d metals.^[4]^ Amongst these metals, manganese has attracted increasing attention.^[5]^ Due to its broad range of oxidation states, manganese complexes present valuable features for (redox) catalysis (Scheme 1b). Indeed, Milstein and co‐workers reported the use of a Mn−PNN pincer complex for the aza‐Michael addition of amines and unsaturated nitriles.^[6]^ The same group also realised the dehydrogenative coupling of amines with methanol to formamides utilising a Mn−PNP pincer catalyst.^[7]^ In a similar vein, our group developed the first *N*‐alkylation of amines with primary alcohols using Mn−PNP catalysts.^[8]^ In addition, Hultzsch and co‐workers reported the *N*‐alkylation of amines with secondary alcohols using a manganese PN^3^‐pincer catalyst.^[9]^ Primary amines could also be coupled with diols to obtain cyclic imines^[10a]^ or pyrroles.^[10b]^

Besides the recent publications of C−N bond formation with manganese catalysts,^[11]^ so far none of these methodologies include olefin hydroamination. In the past few years, formal hydroamination of allylic alcohols has received a lot of attention, with most publications making use of ruthenium catalysts (Scheme 1c). In this respect in 2015, Oe reported a borrowing hydrogen approach with ruthenium catalysts for synthesising *anti*‐Markovnikov γ‐amino alcohols.^[12]^

Recently, Wang and co‐workers employed chiral ruthenium catalysts – similar to Noyori's catalysts – for the asymmetric hydroamination of allylic alcohols with piperazines.^[13]^ Complementary to these original publications, the Buchwald group reported a copper‐phosphine catalyst, in conjunction with silanes, to obtain asymmetric γ‐amino alcohols using hydroxyl amines as nucleophiles.^[14]^ Notably, the only example using a non‐noble metal catalyst for the formal hydroamination of amines with allylic alcohols was developed by the Wang group. By applying an iron PNP‐pincer complex as a catalyst, the corresponding *anti*‐Markovnikov γ‐amino alcohols could be obtained in high yields by a tandem reaction composed of a dehydrogenation/Michael addition/hydrogenation sequence.^[15]^ Inspired by this excellent report and our previous work on manganese pincer catalysis for transfer hydrogenation^[16]^ and dehydrogenation reactions,^[17]]^ we decided to evaluate these type of manganese compounds for the formal hydroamination of allylic alcohols (Scheme 1d).

In preliminary experiments, we examined the reaction of *N*‐methylaniline (**1 a**) with prop‐2‐en‐1‐ol (**2 a**) in the presence of various manganese PNP‐ (**Mn‐1**–**6**) and NNN‐pincer (**Mn‐7**–**8**) complexes, K_3_PO_4_ as base and catalytic amount of sodium borane.^[18]^ As shown in the Table 1, manganese catalysts bearing a NNN‐pincer scaffold were inactive in the catalytic test reaction, while with nearly all manganese PNP‐pincer complexes the corresponding hydroaminated product **3 a** was formed. A lower yield of **3 a** was produced using **Mn‐4**, which possess an electron‐withdrawing ligand (Entry 4). For electron‐donating ligands on the P moiety, a decrease of steric hindrance favoured the formation of product **3 a**, as seen for **Mn‐1**, **Mn‐5** and **Mn‐6**. The best result was obtained with catalyst **Mn‐1** containing the diethyl moiety, while a negligible difference in catalytic activity was observed for the neutral (**Mn‐1**) and cationic (**Mn‐2**) complexes (Entries 1–2). No reaction took place with the *N*‐methyl catalyst **Mn‐3**, which indicates the involvement of the NH‐moiety in the catalytic process via metal ligand cooperation (MLC) (Entry 3).^[18a]^ Applying the commercially available Ru‐MACHO BH_3_ and iron‐PNP (**Fe‐1** and **Fe‐2**) catalysts in the model reaction, the results showed that catalysts **Mn‐1**, **Mn‐2** and **Mn‐6** are more suitable for the formal hydroamination than ruthenium and iron pincer complexes under the chosen reaction conditions.

**Table 1:** Manganese‐catalysed allyl alcohol hydro‐amination.^[a]^

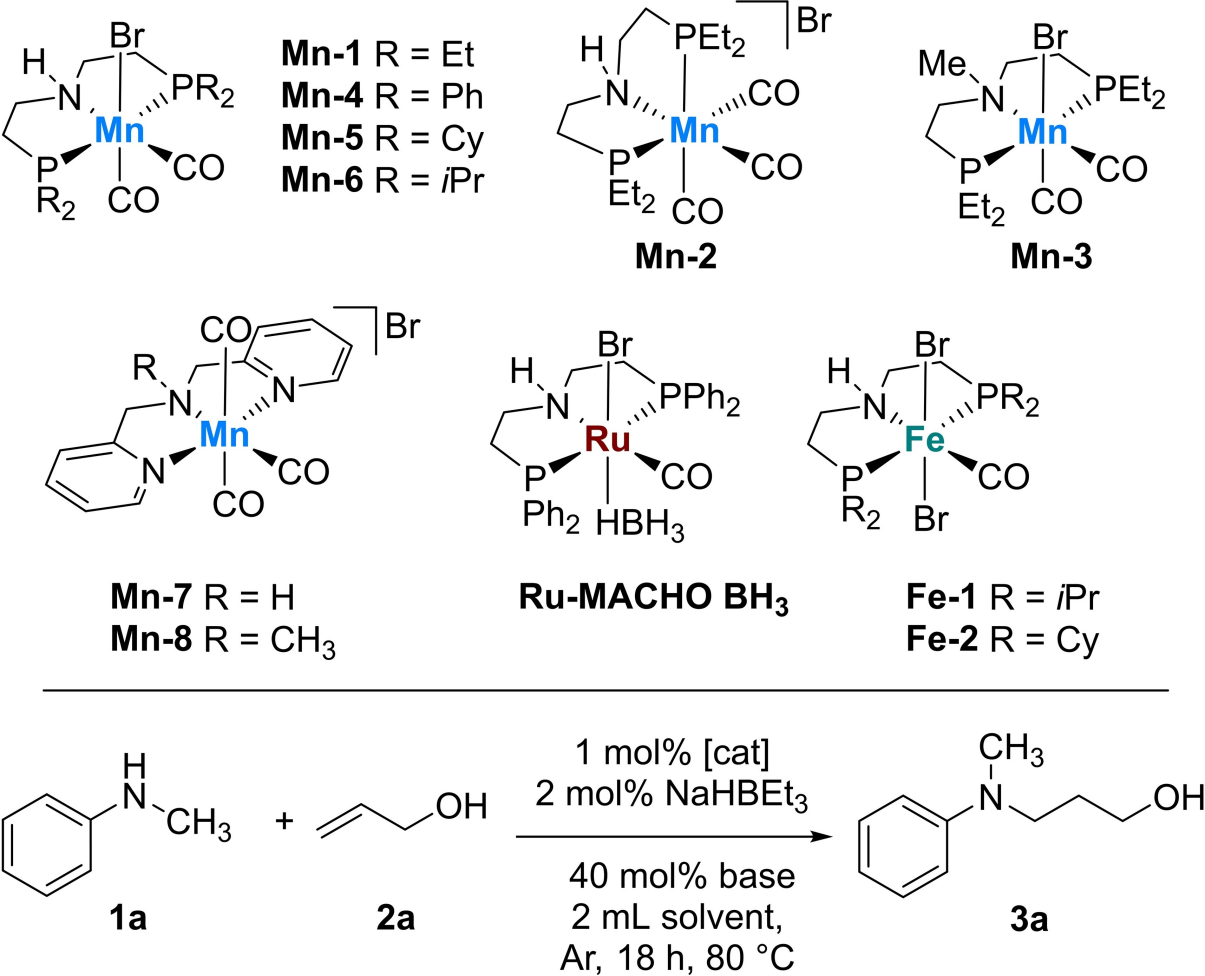			
Entry	Cat. [mol%]	Base [mol%]	Yield **3 a** [%]^[b]^
1	**Mn‐1**	K_3_PO_4_ [40]	68
2	**Mn‐2**	K_3_PO_4_ [40]	67
3	**Mn‐3**	K_3_PO_4_ [40]	0
4	**Mn‐4**	K_3_PO_4_ [40]	49
5	**Mn‐5**	K_3_PO_4_ [40]	54
6	**Mn‐6**	K_3_PO_4_ [40]	64
7	**Mn‐7**	K_3_PO_4_ [40]	0
8	**Mn‐8**	K_3_PO_4_ [40]	0
9^[c]^	**Ru‐MACHO BH_3_**	K_3_PO_4_ [40]	34
10	**Fe‐1**	K_3_PO_4_ [40]	40
11	**Fe‐2**	K_3_PO_4_ [40]	52
12	**Mn‐1**	K_2_CO_3_ [40]	71
13^[d]^	**Mn‐1**	K_2_CO_3_ [40]	76
14^[d]^	**Mn‐1**	K_2_CO_3_ [20]	64
15^[d,e]^	**Mn‐1**	K_2_CO_3_ [40]	81
16^[d,f]^	**Mn‐1**	K_2_CO_3_ [40]	87

^[a]^ *N*‐Methylaniline **1 a** (0.5 mmol), allyl alcohol **2 a** (1 mmol), catalyst (1 mol%), NaHBEt_3_ (2 mol%), K_3_PO_4_ (40 mol%), toluene (2 mL), 18 h, 80 °C. ^[b]^ Yields are determined by internal standard. ^[c]^ No NaHBEt_3_. ^[d]^ Cyclohexane (2 mL). ^[e]^ 18 h, 60 °C. ^[f]^ 24 h, 60 °C.

After the catalyst screening, reactions conditions such as solvent, base and temperature were optimised (see ESI). Different polar aprotic solvents were tested, while oxygen‐containing solvents presented low hydroamination yields. The most suitable solvent for hydroamination of *N*‐methylaniline was cyclohexane providing yields up to 87% at 60 °C and 24 h (Entries 13, 15, 16). The best result in the base screening was achieved in the presence of non‐stoichiometric amounts of simple K_2_CO_3_. (Entry 12). Here, a certain amount of base was found to be crucial to achieve high product yields. While with 20 mol% of K_2_CO_3_ the yield of **3 a** dropped down (Entry 14), no further improvements were achieved with base loading above 40 mol% (Table S1). These findings agree with the results of the Wang group demonstrating the beneficial role of base for the dehydrogenation step of the allylic alcohol.^[15]^

Next, a series of different amines and *N*‐heterocycles were tested for manganese‐catalysed hydroamination (Scheme 2). For secondary amines, an increase of steric bulk for *N*‐substituted anilines led to decreased yields (products **3 a**–**c** and **3 e**). No hydroaminated product was observed for diphenylamine, which might be related to the high steric hindrance and decreased nucleophilicity on the nitrogen atom. Furthermore, the substituent position of aromatic amines played an important role (see products **3 f**–**g**). Formal hydroamination of *N*‐allyl aniline was carried out, leading to 53% yield of **3 i**. Different substituents, such as Br, F, CF_3_ and OMe were tolerated for allyl alcohol hydroamination (products **3 j**–**m**), whilst the nitro group‐containing substrate was not reactive. Gratifyingly, an α‐amino alcohol reacted well to provide the amino diol product **3 n** under our standard reaction conditions.

**Scheme 2:**
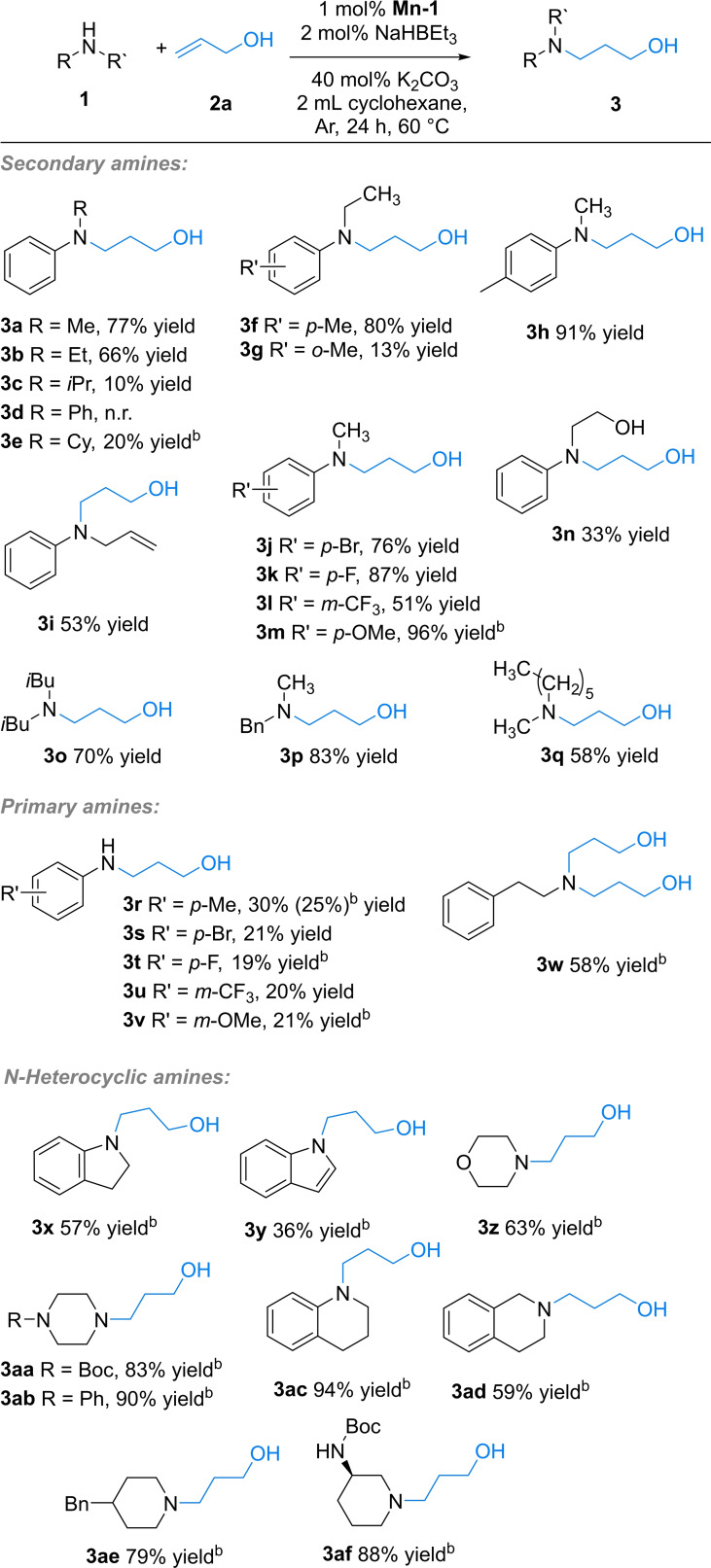
Manganese catalysed formal hydroamination of allyl alcohol. ^a)^ Amine (0.5 mmol), allyl alcohol (1 mmol), **Mn‐1** (1 mol%), NaHBEt_3_ (2 mol%), cyclohexane (2 mL), K_2_CO_3_ (40 mol%), 60 °C, 24 h, isolated yields. ^b)^ **Mn‐1** (2 mol%), NaHBEt_3_ (4 mol%).

Furthermore, the reaction of *N*‐methyl aniline **1 a** and *N*‐methyl toluidine **1 h** with allyl alcohol **2 a** were realized in a 5 mmol scale giving the respective products in 71% (**3 a**) and 64% (**3 h**) isolated yields after 24 hours at 60 °C (SI). Although in both cases slightly reduced product yields were obtained, the general practicability of the catalytic protocol could be successfully demonstrated.

Aliphatic secondary amines were also successfully converted with yields between 58–83% (products **3 o**–**q**). As observed for aromatic amines, reactions of aliphatic amines were also influenced by steric hindrance. For primary anilines, hydroamination products were obtained in significantly lower yields (**3 r**–**3 v**). This trend can be explained by a lower electrophilicity of the neutral imine intermediate which will be formed by condensation with **2 a** according to the previously discussed mechanism.^[15]^ Interestingly, reaction of an aliphatic primary amine with prop‐2‐en‐1‐ol (**2 a**) resulted in the formation of the double hydroamination product **3 w** in 58% yield. Given that *N*‐heterocycles are very relevant constituents of pharmaceutical drugs, we carried out several reactions with different heterocyclic motifs, such as pyrrolidine, piperidine, piperazine, morpholine, and indole. Here, product yields of 36–94% were achieved for γ‐amino alcohols with the different heterocycles. In general, six‐membered heterocycles presented higher reactivity than five‐membered heterocycles for allyl alcohol hydroamination. It is assumed that the lower nitrogen nucleophilicity in case of indole led to a decreased yield of **3 y** compared to **3 x**. For piperazines, no significant differences were observed for various substituted derivatives (products **3 aa**–**ab**). In the case of tetrahydroquinolines, the nitrogen position had a considerable effect on hydroamination yield (products **3 ac**–**ad**).

Due to the high electron‐withdrawing ability of the *tert*‐butoxycarbonyl (Boc) protecting group, the mono‐hydroaminated product **3 af** could be selectively obtained in 88% yield. This example offers many possibilities for posterior functionalisation of such γ‐amino alcohols.

Next, we also evaluated the scope of allylic alcohols using 1‐phenylpiperazine **4 a**, (Scheme 3) as piperazine rings are commonly encountered in many leading pharmaceuticals.^[19]^ Indeed, several allylic alcohols could be hydroaminated with 1‐phenylpiperazine **4 a** in yields ranging from 25–82%. Hydroamination yield was not so affected by steric hindrance of a methyl group in the olefin of an allylic alcohol (product **5 a**). However, no reaction took place using 3‐methyl‐but‐2‐ene‐1‐ol, which can be attributed to the difficulty for piperazine to attack this sterically hindered trisubstituted olefin. On the other hand, an allylic alcohol containing an endocyclic olefinic bond gave the desired product **5 b** in 41% yield.

**Scheme 3:**
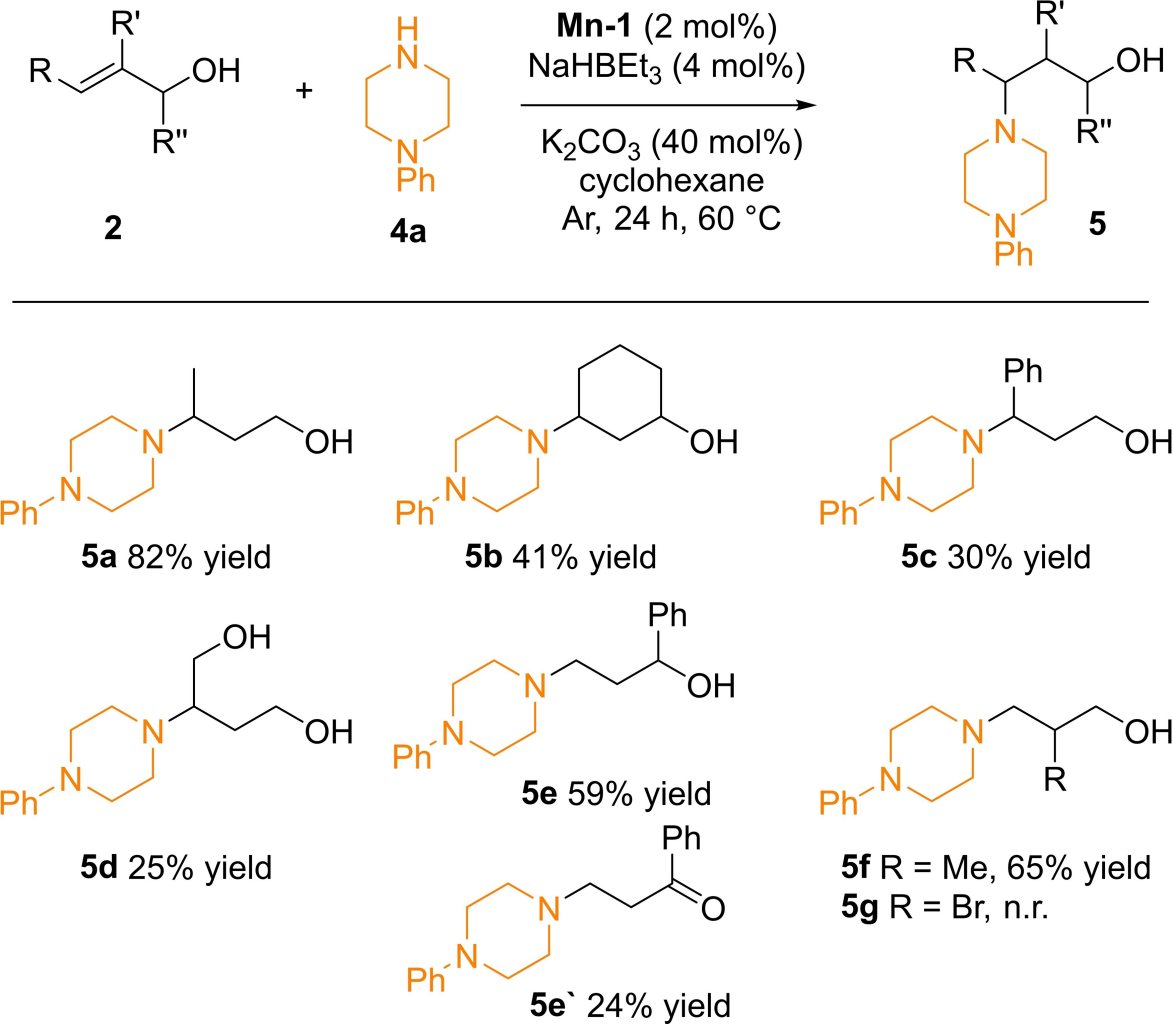
Substrate scope of allylic alcohol formal hydroamination. ^a)^ 1‐Phenylpiperazine (0.5 mmol), alcohol (1 mmol), **Mn‐1** (2 mol%), NaHBEt_3_ (4 mol%), cyclohexane (2 mL), K_2_CO_3_ (40 mol%), 60 °C, 24 h, isolated yields.

The natural product cinnamyl alcohol successfully underwent hydroamination, too, leading to 30% yield of **5 c**. This decrease in reactivity in case of product **5 c** compared to **5 a** might be related to the bulkiness of the conjugated phenyl ring, and by the electron delocalisation of the ring. Furthermore, the symmetric allylic diol but‐2‐ene‐1,4‐diol could be selectively hydroaminated to give product **5 d**. For the α‐substituted alcohol 1‐phenyl‐2‐propen‐1‐ol (**2 e**), the alcohol (**5 e**) as well as the ketone (**5 e‘**) containing hydroamination product were obtained in 59% and 24% isolated yields, respectively. This shows that even the ketone intermediates formed after the Michael addition step can be hydrogenated to produce the corresponding γ‐amino alcohol. Finally, we tested substrates with substituents at the carbon β‐position, and the respective product **5 f** was obtained in 65% yield. However, the more sensitive bromide‐substituted alcohol did not react.

In summary, the successful application of a well‐defined manganese PNP‐pincer complex in the formal hydroamination of allylic alcohols with primary, secondary amines and *N*‐heterocycles was reported. The corresponding γ‐amino alcohols were obtained in up to 94% yield in a tandem reaction combining dehydrogenation/Michael addition/hydrogenation sequence. The properties of these non‐innocent pincer ligands allowed application of this non‐noble metal catalyst in organic synthesis with a broad substrate scope.

## Experimental Section

Reactions were carried out in a heat‐gun dried under vacuum 25 mL Schlenk flask equipped with a magnetic stirring bar. The catalyst (2.2 mg, 0.005 mmol, [1 mol%] **Mn‐1**) was added inside a glovebox. Outside the glovebox 0.5 mL of cyclohexane and 10 μL (1 M solution in toluene) of NaHBEt_3_ (0.01 mmol, [2 mol%]) was added and the mixture was stirred for 10 min at room temperature. After this, *N*‐methylaniline (**1 a**) (54 mg, 0.5 mmol), prop‐2‐en‐1‐ol (**2 a**) (59 mg, 1 mmol), K_2_CO_3_ (0.2 mmol, [40 mol%]) and 1.5 mL of cyclohexane were added to Schlenk flask. The flask was inserted in an aluminium block and heated to 60 °C for 24 hours. After reaction completion, the flask was cooled down to room temperature and 3 mL of CH_2_Cl_2_ were added to the mixture for GC analysis. Dodecane was used as internal standard for GC measurements.

All other hydroamination products were isolated by column chromatograph and characterised by NMR spectroscopy. For purification, the reaction mixture was cooled to room temperature, transferred to a round‐bottom flask, adsorbed on Celite, and concentrated under vacuum to obtain a dried powder which was separated using CombiFlash Rf 200 (Teledyne) equipment. Product was concentrated under vacuum to obtain product yield.

## Supporting information

As a service to our authors and readers, this journal provides supporting information supplied by the authors. Such materials are peer reviewed and may be re‐organized for online delivery, but are not copy‐edited or typeset. Technical support issues arising from supporting information (other than missing files) should be addressed to the authors.

Supporting Information
