# Supplementary material for: Catalytic Formal Hydroamination of Allylic Alcohols Using Manganese PNP‐Pincer Complexes
Source: Adv Synth Catal. 2021 Mar 17;363(17):4177–81. doi: 10.1002/adsc.202100081 (PMC8519145; doi:10.1002/adsc.202100081)
Supplement: Supplementary file 1 — Supporting Information [file ADSC-363-4177-s001.pdf]

Supporting Information

Catalytic Formal Hydroamination of Allylic Alcohols Using Manganese PNP-Pincer Complexes

Leandro Duarte de Almeida, Florian Bourriquen, Kathrin Junge,* and Matthias Beller*
© 2021 The Authors. Advanced Synthesis & Catalysis published by Wiley-VCH GmbH. This is an open access article under the terms of the Creative Commons Attribution License, which permits use, distribution and reproduction in any medium, provided the original work is properly cited.

Supporting Information

Catalytic Hydroamination of Allylic Alcohols using Manganese PNP-Pincer Complexes

Leandro Duarte de Almeida[a], Florian Bourriquen[a], Kathrin Junge,[a]* Matthias Beller[a]*

Supporting Information

Table of Contents

1. General information of experimental section 2

2. General procedure for hydroamination reaction 2

2.1 General procedure for hydroamination reaction 2

2.2 Catalytic procedure for hydroamination reaction in 5 mmol scale 3

3. Complex synthesis 4

3.1 Synthesis of diethyl PNP-pincer ligand 4

3.1.1 *N,N*-bis(2-chloroethyl)-1,1,1-trimethylsilanamine 4

3.1.2 Bis(2-(diethylphosphaneyl)ethyl)amine 5

3.2 Synthesis of Mn pincer catalyst **Mn-1** 7

4. Table S1. Optimization of reaction conditions for allyl alcohol hydroamination 9

5. Spectroscopic data of hydroamination products 10

6. NMR spectra of isolated hydroamination products 24

7. Literature 103

1. General information of experimental section

All the reactants were obtained from commercial sources and used as received unless otherwise specified. All solvents used in the experiments were degassed and stored with previously heated 3Å molecular sieves. Schlenk techniques were applied for all experiments under inert atmosphere. 0.20 mm silica gel 60 with fluorescent indicator UV_{254} TLC plates were used to monitor reaction progress, HPLC grade solvents were used as eluent. For flash column chromatography HPLC heptane, ethyl acetate and dichloromethane and acetone ≥99.8% were used. ^{1}H NMR spectrum were measured in AV-300, AV-400 and Fourier-300 Bruker equipment. Signals were reported relative to $CDCl_3$ (δ 7.26 ppm for ^{1}H and 77.26 ppm for ^{13}C), MeOH-*d*4 (δ 4.78 ppm for ^{1}H and 49.15 ppm for ^{13}C) and C_6H_6 (δ 7.16 ppm for ^{1}H and 128.39 ppm for ^{13}C). Coupling constant were reported as *J* value in Hz. Multiplets were reported as: b – broad, s – singlet, d – doublet, t – triplet, q – quartet, p – pentet, hept – heptet, dd – doublet of doublets, td – triplet of doublets , ddt – doublet of doublet of triplets , dt – doublet of triplets , ddd – doublet of doublet of doublets , dq – doublet of quartets and m – multiplet. Gas chromatography was performed on Agilent 7890A equipped with FID detector and HP5 column. High resolution mass spectra measurements were recorded on MAT 95XP ThermoFisher Mass Spectrometer using Electrospray Ionization mode.

2. Catalytic procedure for hydroamination reaction

2.1 General procedure for hydroamination reaction

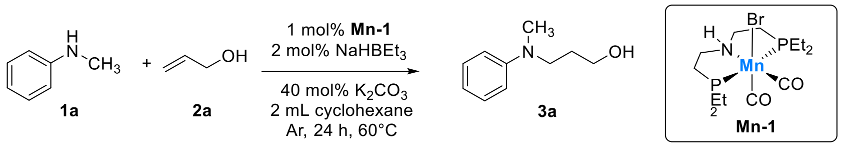

C_7H_9N **107,07** | C_3H_6O **58,04** | $C_{10}H_{15}NO$ **165,12** | $C_{14}H_{29}BrMnNO_2P_2$ **439,02**

Reactions were carried out in a heat-gun dried under vacuum 25 mL Schlenk flask equipped with a magnetic stirring bar. Catalyst (2.2 mg, 0.005 mmol, [1 mol%] **Mn-1**) was added inside a glovebox. Outside the glovebox it was added 0.5 mL of cyclohexane and 10 μL (1 M solution in toluene) of $NaHBEt_3$ (0.01 mmol, [2 mol%]), the mixture was stirred for 10 min at room temperature. After this, N-methylaniline (54 mg, 0.5 mmol), allyl alcohol (59 mg, 1 mmol), K_2CO_3 (0.2 mmol, [40 mol%]) and 1.5 mL of cyclohexane were added to Schlenk flask. The flask was inserted in an aluminium block and heated to 60 °C for 24 hours. After reaction completion, the flask was cooled down to room temperature and added 3 mL of CH_2Cl_2 to the mixture for GC analysis. Dodecane was used as internal standard for GC measurements.

For hydroamination products purification, reaction mixture was cooled down to room temperature, transferred to a round-bottom flask, adsorbed on Celite and concentrated under vacuum to obtain a dried powder which was separated using CombiFlash Rf 200 (Teledyne) equipment. Product was concentrated under vacuum to obtain product yield.

2.2 Catalytic procedure for hydroamination reaction in 5 mmol scale

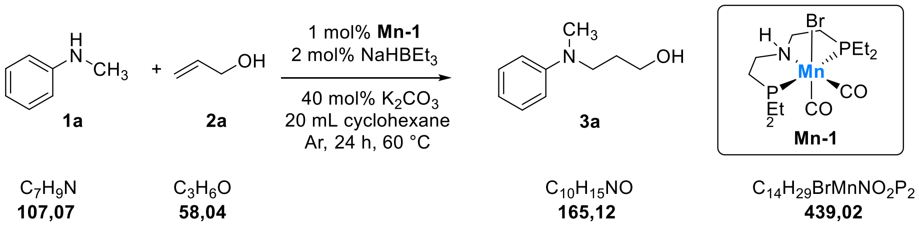

Reactions were carried out in a heat-gun dried under vacuum 50 mL Schlenk flask equipped with a magnetic stirring bar. Catalyst (22 mg, 0.05 mmol, [1 mol%] **Mn-1**) was added inside a glovebox. Outside the glovebox 5 mL of cyclohexane and 100 µL (1 M solution in toluene) of $NaHBEt_3$ (0.1 mmol, [2 mol%]) was added and the mixture was stirred for 10 min at room temperature. After this, N-methylaniline (540 mg, 5 mmol), allyl alcohol (590 mg, 10 mmol), K_2CO_3 (2 mmol, [40 mol%]) and 15 mL of cyclohexane were added to Schlenk flask. The flask was inserted in an aluminium block and heated to 60°C for 24 hours. After reaction completion, the flask was cooled down to room temperature and added 30 mL of CH_2Cl_2 to the mixture for GC analysis.

Product **3a** was isolated by column chromatography using 1:5 ethyl acetate/heptane as eluent. Product yield: 0.584 g (71% yield). Obtained as a yellow oil. Rf = 0.108 (1:5 ethyl acetate/heptane).

Product **3h** was isolated by column chromatography using 1:5 ethyl acetate/heptane as eluent. Product yield: 0.573 g (64% yield). Obtained as a yellow oil. Rf = 0.139 (1:5 ethyl acetate/heptane).

3. Complex synthesis

3.1 Synthesis of diethyl PNP-pincer ligand

3.1.1 *N,N*-bis(2-chloroethyl)-1,1,1-trimethylsilanamine

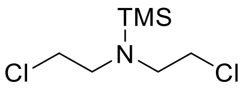

The title compound was synthesized referring to a previously reported procedure.[1] To a suspension of bis(2-chloroethyl)amine hydrochloride (10 g, 56.0 mmol) in 100 mL of Et_2O, 0.25 mL of DMSO and triethylamine (23.4 mL, 17.0 g, 168.0 mmol) were added at 0 °C. Chlorotrimethylsilane (24.9 mL, 21.3 g, 196.0 mmol) was added dropwise over 30 min. The mixture was stirred at 0 °C for one hour and allowed warm to room temperature and stirred for further four days. The solution was filtered, the volatiles were removed under reduced pressure. The title compound was isolated as an orange oil (81%, 9.73 g).

NMR: ^{1}H NMR (300 MHz, C_6D_6) δ 3.02 – 2.94 (t, 4H), 2.74 – 2.65 (t, 4H), -0.09 (s, 9H).

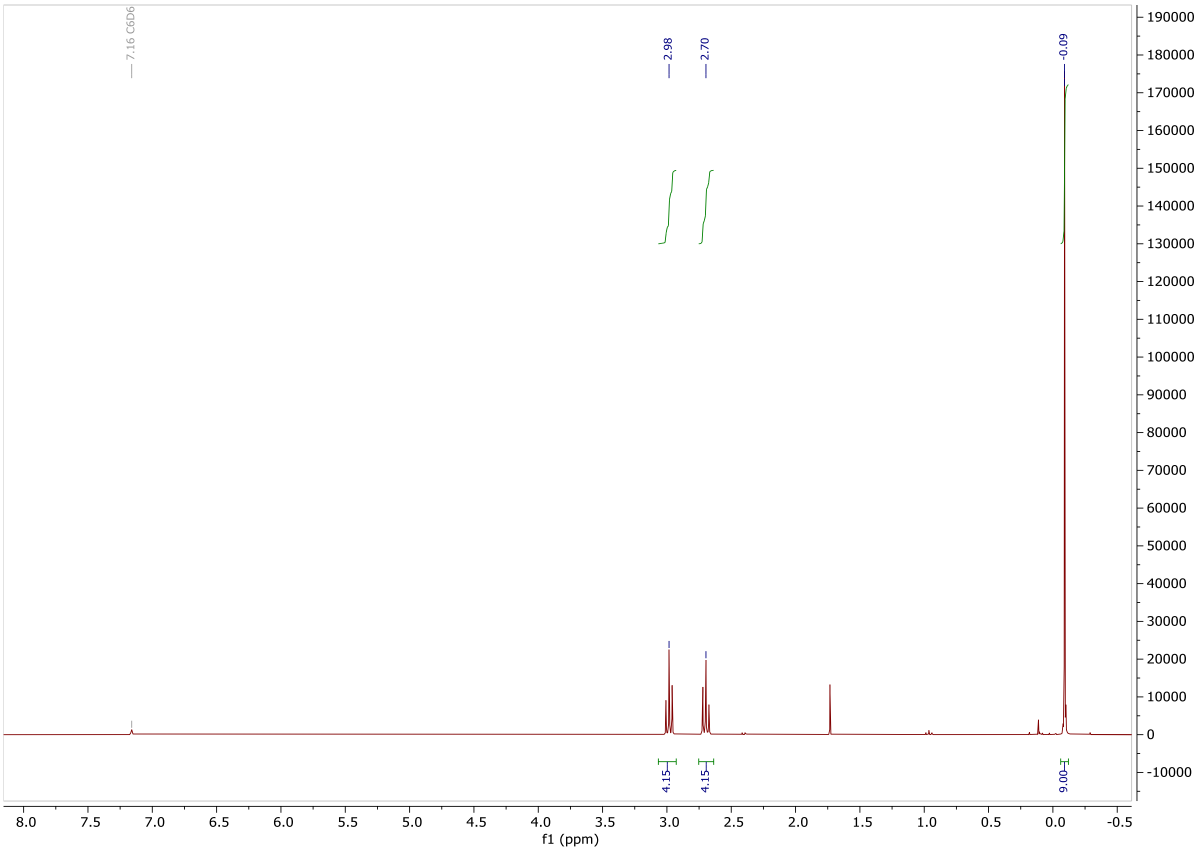

3.1.2 Bis(2-(diethylphosphaneyl)ethyl)amine

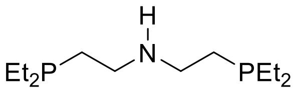

The title compound was synthesized similarly to a previously reported procedure.[2] To a solution of Et_2PH (1 g, 11 mmol) in 50 mL of hexane at – 78 °C, BuLi (5 mL, 12.5 mmol, 2.5M in hexane solution) was added dropwise over 20 min. The mixture could return to room temperature and stirred at 35 °C for 1h. Et_2PLi precipitated as a white solid. After return to room temperature 20 mL of THF were added. The solution was cooled to - 40 °C, and a solution of *N*,*N*-bis(2-chloroethyl)-1,1,1-trimethylsilanamine (1.178 g, 5.55 mmol) in 10 mL of THF was added dropwise under vigorous stirring. The mixture was finally allowed to room temperature and heated to 60 °C overnight. After return to room temperature, the ligand was treated with TBAF (16.5 mL of a 1M in THF in 50 mL of degassed water) and the resulting mixture was stirred vigorously for two days at 60 °C. The mixture was cooled room temperature, the phases were separated, and the aqueous phase was extracted with diethyl ether (3 x 20 mL). The organic phases were combined, dried over $MgSO_4$, filtered, and the volatiles were removed under reduced pressure, yielding the title compound as a colourless oil. (83%, 1.14 g).

NMR: ^1H NMR (300 MHz, C_6D_6) δ 2.78 – 2.64 (m, 4H), 1.53 – 1.43 (m, 4H), 1.29 – 1.21 (m, 8H), 1.06 – 0.90 (m, 12H). ^{13}C NMR (75 MHz, C_6D_6) δ 54.39, 47.64, 47.40, 30.25, 28.33, 28.13, 27.32, 21.04, 19.81, 19.64, 14.40, 10.04, 9.98, 9.86, 9.81. ^{31}P NMR (122 MHz, C_6D_6) δ -27.13.

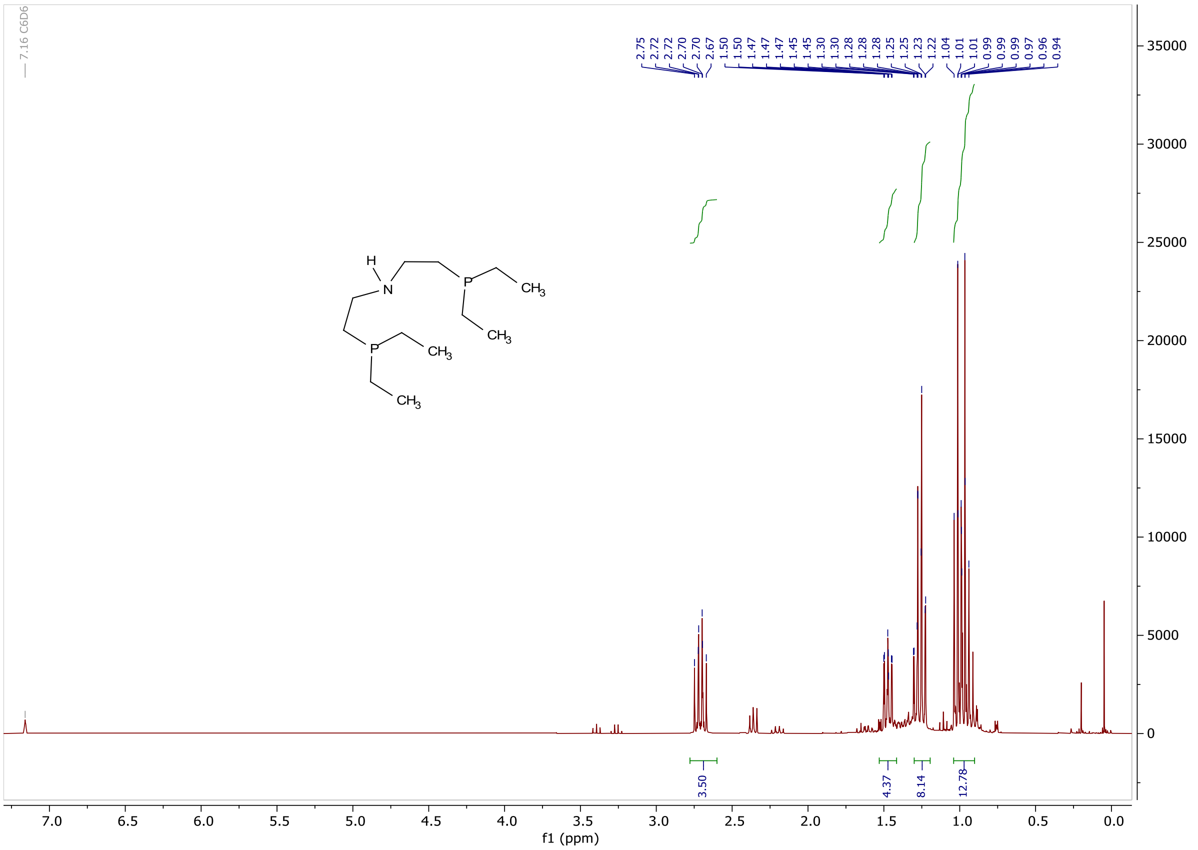

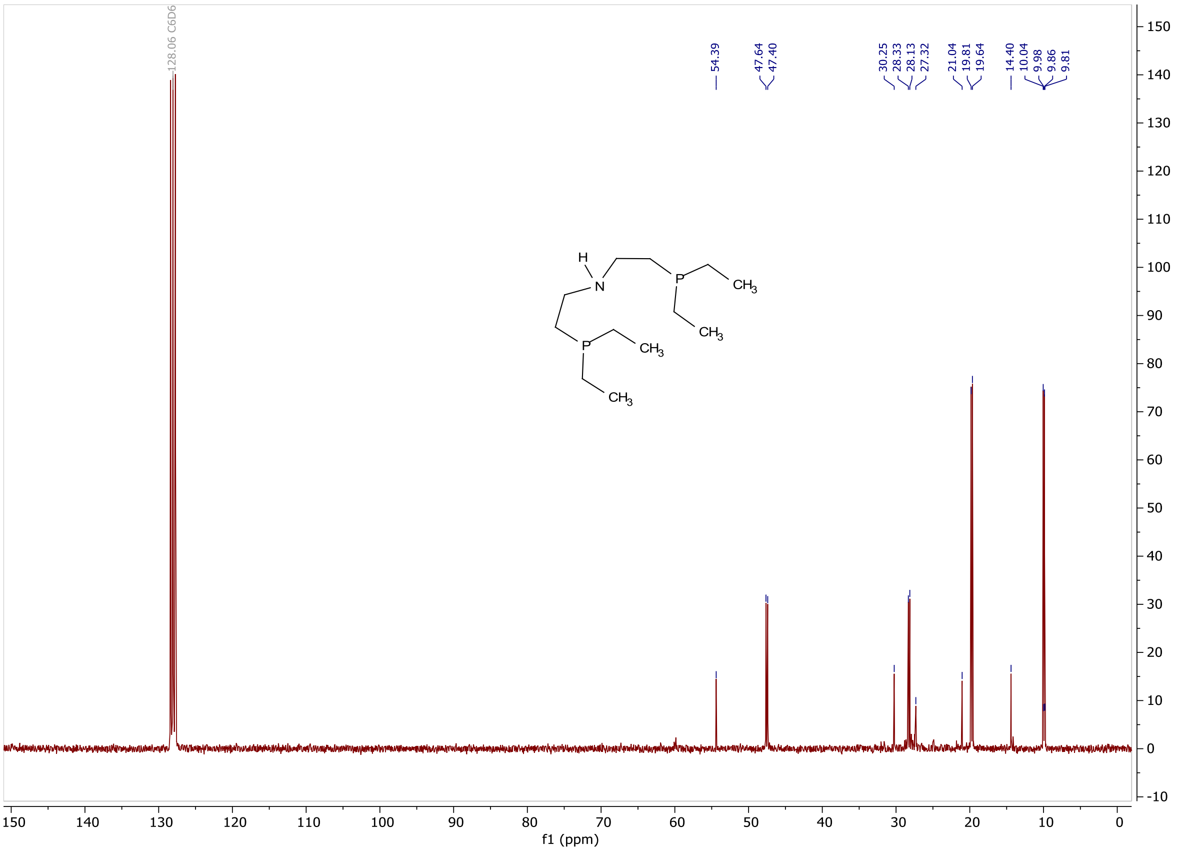

128.06 C6D6
54.39
47.64
47.40
30.25
28.33
28.13
27.32
21.04
19.81
19.64
14.40
10.04
9.98
9.86
9.81
H
N
P
CH3
CH3
P
CH3
CH3
f1 (ppm)

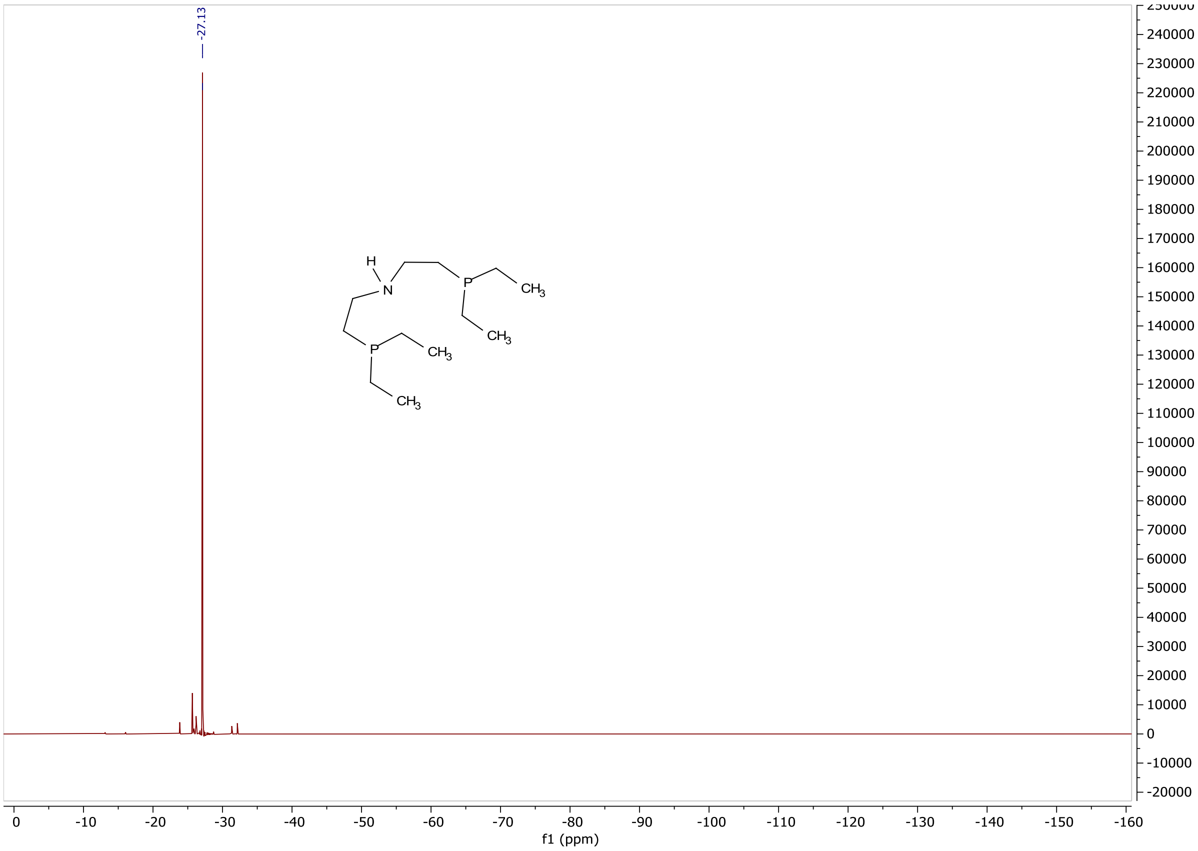

-27.13
H
N
P
CH3
CH3
P
CH3
CH3
f1 (ppm)

3.2 Synthesis of Mn pincer catalyst Mn-1

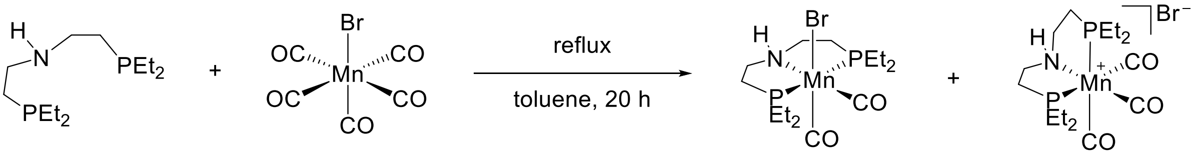

Mn-1 **Mn-2**

The procedure for catalyst synthesis was already reported previously.[2] To the orange-yellow suspension of $Mn(CO)_5Br$ (315 mg, 1.61 mmol) in toluene (15 mL) $[HN(CH_2CH_2P(Et)_2)_2]$ (300 mg, 1.23 mmol, dissolved in 2 mL toluene) was added. A solid was formed during this time and it was heated to reflux and further stirred for 20 h under argon flow. Reaction mixture was cooled down to room temperature and concentrated under vacuum. Crude was thoroughly washed with toluene several times and the solvent was transferred to another Schlenk. Remaining pale-yellow solid was dried in vacuum and yielded **Mn-2** (64%). The dark yellow solvent from the washings was evaporated and gave a dark orange yellow sticky solid after vacuum evaporation. The solid was washed by cannula filtration procedure with heptane three times and two times with methanol (2 x 2 mL) affording **Mn-1** as an orange yellow solid (18%). **Mn-2** can be transferred into **Mn-1** be reflux in toluene (120 °C) for 16 hours yielding additional 44% of **Mn-1** giving 62% overall yield of **Mn-1**.

NMR: ^1H NMR (300 MHz, C_6D_6) δ 2.68 (b, 1H), 2.31-2.23 (m, 4H), 2.19-2.02 (m, 2H), 1.86–1.57 (m, 6H), 1.45-1.36 (m, 2H), 1.32-1.21 (m, 2H), 1.19-0.97 (m, 12H). ^{13}C MNR (75 MHz, C_6D_6) δ 51.67; 27.25; 19.55; 19.45; 16.98; 8.75; 8.57. ^{31}P NMR (122 MHz, C_6D_6) δ 68.25.

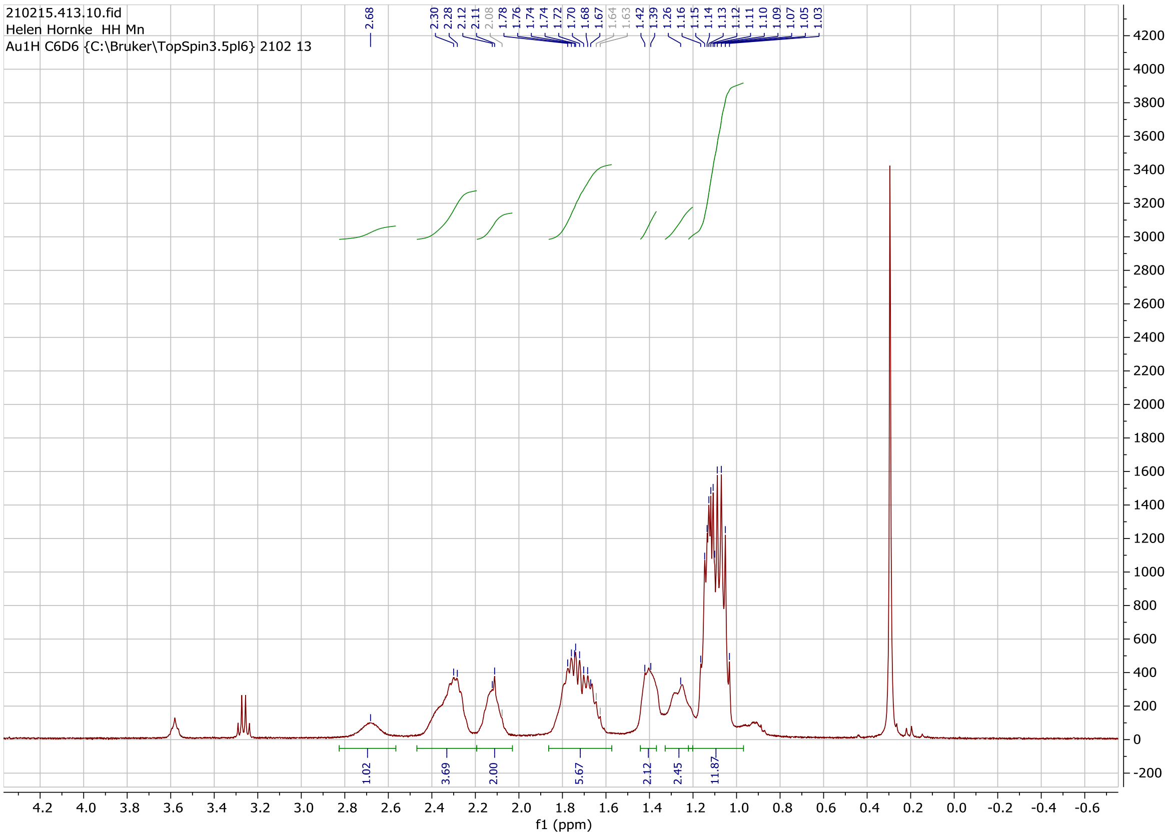

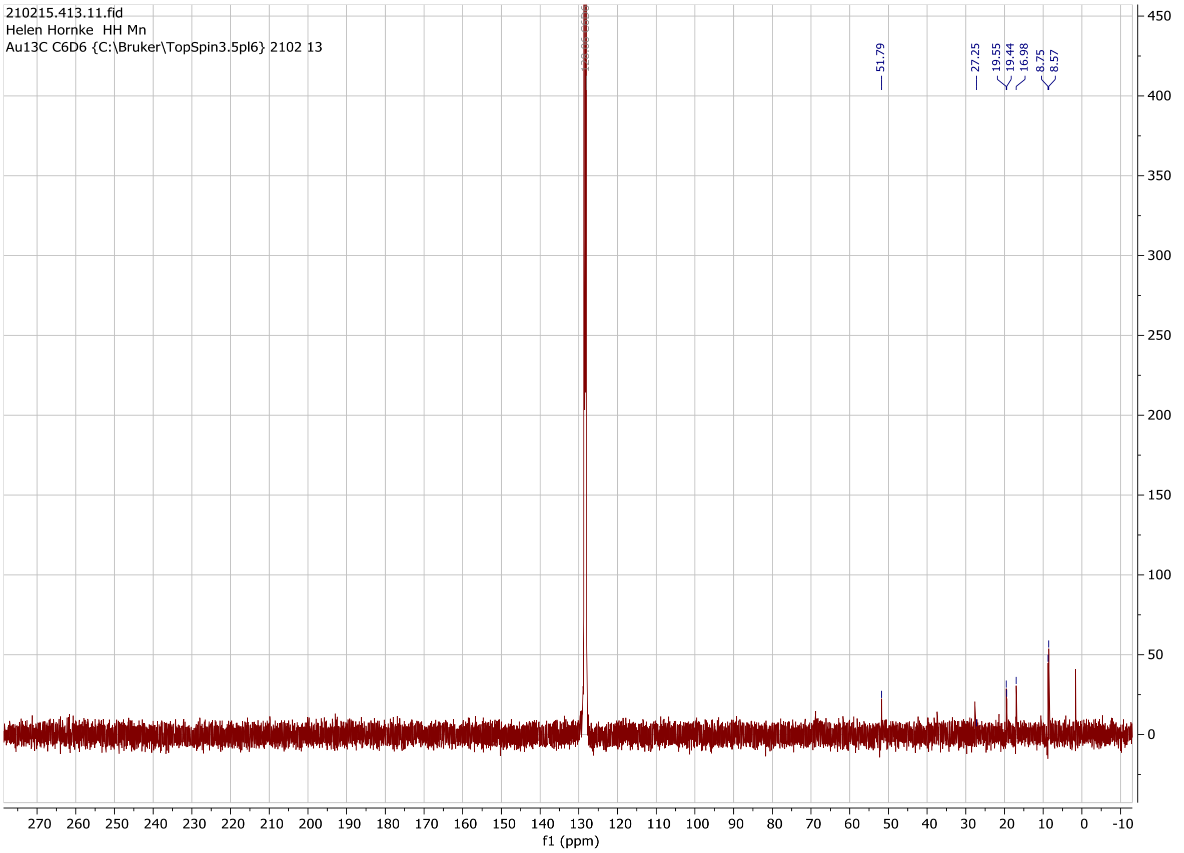

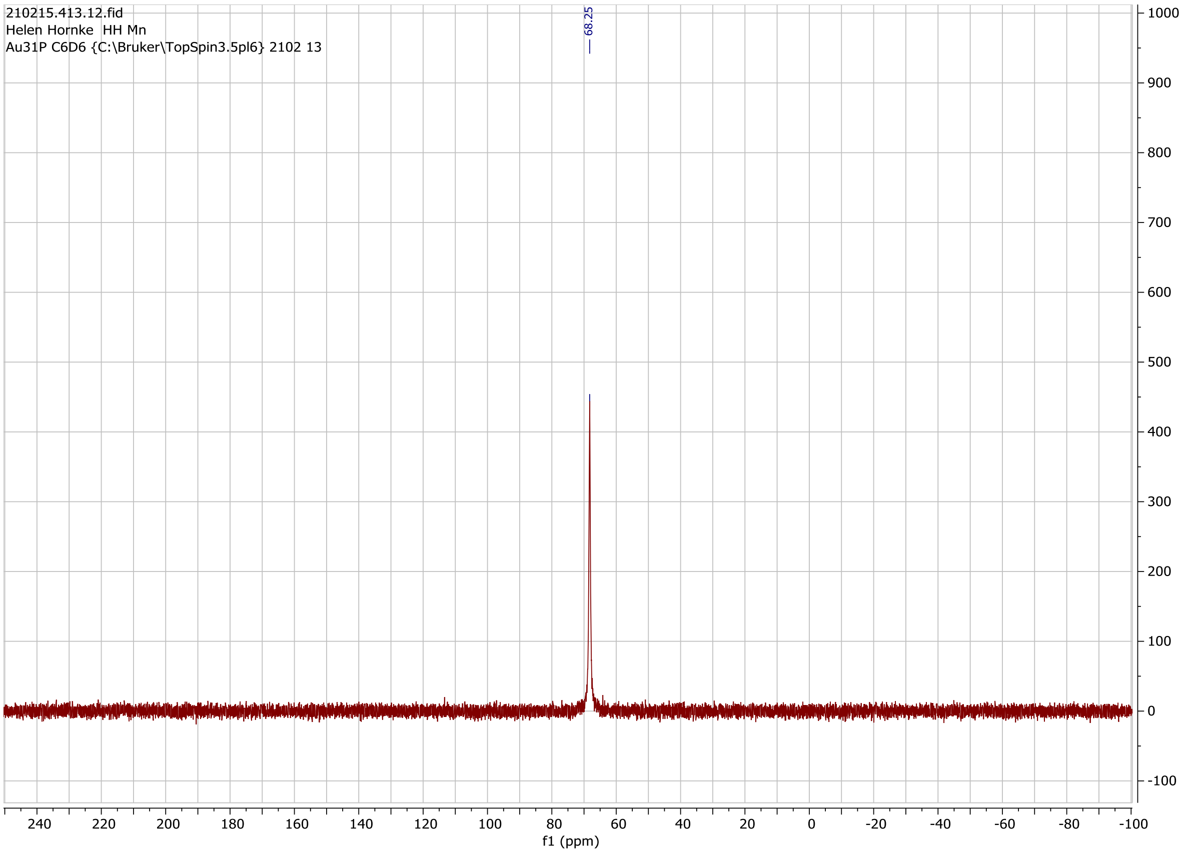

Catalysts **Mn-3**[3], **Mn-4**[4], **Mn-5**[5], **Mn-6**[5], **Mn-7**[6], **Mn-8**[6], **Fe-1**[7] and **Fe-2**[8] were prepared according to previously reported methodology.

4. Table S1. Optimization of reaction conditions for allyl alcohol hydroamination[a]

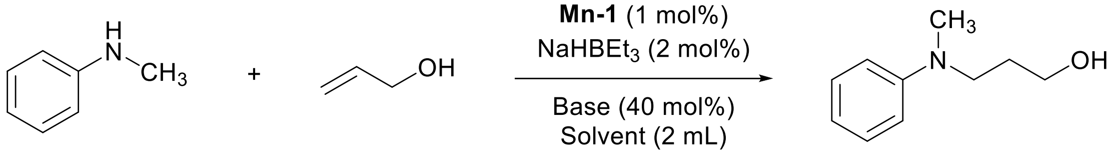

1a 2a 3a

Entry	Base	Solvent	Time (h)	Temperature (°C)	Yield[b] (%)
1	K_3PO_4	Toluene	18	80	68
2	Na_2CO_3	Toluene	18	80	53
3	NaOEt	Toluene	18	80	0
4	K_2CO_3	Toluene	18	80	71
5	K_2CO_3	THF	18	80	24
6	K_2CO_3	1,4-dioxane	18	80	0
7	K_2CO_3	Heptane	18	80	70
8	K_2CO_3	Cyclohexane	18	80	76
9[c]	K_2CO_3	Cyclohexane	18	80	64
10	K_2CO_3	Cyclohexane	24	40	6
11	K_2CO_3	Cyclohexane	18	60	81
12	K_2CO_3	Cyclohexane	24	60	87
13[d]	K_2CO_3	Cyclohexane	24	60	38
14[d,e]	K_2CO_3	Cyclohexane	24	60	48
15[e]	K_2CO_3	Cyclohexane	24	60	75

[a] amine (0.5 mmol), allyl alcohol (1 mmol), catalyst (1 mol%), $NaHBEt_3$ (2 mol%), solvent (2 mL), base (40 mol%), under argon atmosphere. [b] GC yields. [c] K_2CO_3 (20 mol%). [d] allyl alcohol (1.5 mmol). [e] K_2CO_3 (60 mol%).

5. Spectroscopic data of hydroamination products

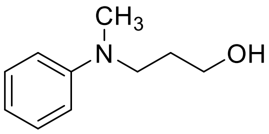

3-(Methyl(phenyl)amino)propan-1-ol (3a): The product **3a** was isolated as a yellow oil by column chromatography using 1:5 ethyl acetate/heptane as eluent. Product yield: 0.0633 g (77% yield). Rf = 0.108 (1:5 ethyl acetate/heptane).

NMR: ^{1}H NMR (300 MHz, $CDCl_3$) δ 7.32 – 7.17 (m, 2H), 6.84 – 6.68 (m, 3H), 3.74 (t, *J* = 6.0 Hz, 2H), 3.45 (t, *J* = 6.9 Hz, 2H), 2.94 (s, 3H), 1.98 (s, 1H), 1.85 (p, 2H). ^{13}C NMR (75 MHz, $CDCl_3$) δ 149.73, 129.31, 116.92, 113.08, 61.13, 50.36, 38.62, 29.73.

HRMS: calcd for $C_{10}H_{15}NO$ ($[M+H]^+$): 165.1148, found: 165.1149.

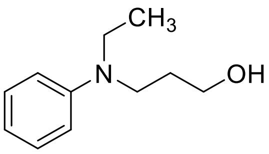

3-(Ethyl(phenyl)amino)propan-1-ol (3b): The product **3b** was isolated as a yellow oil by column chromatography using 1:5 ethyl acetate/heptane as eluent. Product yield: 0.0593 g (66% yield). Rf = 0.157 (1:5 ethyl acetate/heptane).

NMR: ^{1}H NMR (300 MHz, $CDCl_3$) δ 7.30 – 7.17 (m, 2H), 6.84 – 6.63 (m, 3H), 3.75 (t, *J* = 5.9 Hz, 2H), 3.43-3.34 (p, *J* = 7.0 Hz, 4H), 1.89-1.81 (p, 3H), 1.15 (t, *J* = 7.0 Hz, 3H). ^{13}C NMR (75 MHz, $CDCl_3$) δ 129.41, 116.56, 113.23, 61.31, 47.89, 45.60, 30.31, 12.14.

HRMS: calcd for $C_{11}H_{17}NO$ ($[M+H]^+$): 179.1304, found: 179.1305.

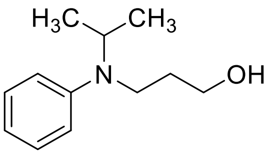

3-(Isopropyl(phenyl)amino)propan-1-ol (3c): The product **3c** was isolated as a yellow oil by column chromatography using 1:5 ethyl acetate/heptane as eluent. Product yield: 0.0093 g (10% yield). Rf = 0.167 (1:5 ethyl acetate/heptane).

NMR: ^{1}H NMR (300 MHz, $CDCl_3$) δ 7.31 – 7.17 (m, 2H), 6.89 (d, *J* = 7.8 Hz, 2H), 6.80 (tt, *J* = 7.3, 1.1 Hz, 1H), 3.93 (hept, *J* = 6.6 Hz, 1H), 3.75 (t, *J* = 5.8 Hz, 2H), 3.28 (t, 2H), 2.47 (b, 1H), 1.79 (p, 2H), 1.17 (d, *J* = 6.6 Hz, 6H). ^{13}C NMR (75 MHz, $CDCl_3$) δ 129.23, 118.55, 116.81, 62.27, 51.39, 42.23, 30.84, 19.86.

HRMS: calcd for $C_{12}H_{19}NO$ ([M+H]$^+$): 193.1461, found: 193.1466.

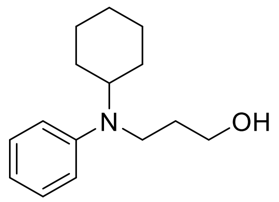

3-(Cyclohexyl(phenyl)amino)propan-1-ol (3e): The product **3e** was isolated as a yellow oil by column chromatography using 1:5 ethyl acetate/heptane as eluent. Product yield: 0.0086 g (7% yield) for 1 mol% catalyst and 0.0234 g (20% yield) for 2 mol% catalyst. Rf = 0.182 (1:5 ethyl acetate/heptane).

NMR: ^{1}H NMR (300 MHz, $CDCl_3$) δ 7.31 – 7.17 (m, 2H), 6.86 (d, *J* = 8.1 Hz, 2H), 6.77 (t, *J* = 7.1 Hz, 1H), 3.74 (t, *J* = 5.8 Hz, 2H), 3.33 (t, *J* = 7.0 Hz, 2H), 2.37 (b, 1H), 1.96 – 1.63 (m, 8H), 1.50 – 1.47 (m, 4H), 1.16-1.06 (m, 1H). ^{13}C NMR (75 MHz, $CDCl_3$) δ 148.75, 129.22, 118.17, 116.35, 62.20, 60.10, 43.26, 31.14, 30.61, 26.34, 26.02.
HRMS: calcd for $C_{15}H_{23}NO$ ([M+H]$^+$): 233.1774, found: 233.1773.

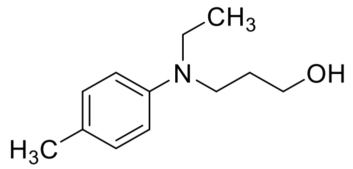

3-(Ethyl(p-tolyl)amino)propan-1-ol (3f): The product **3f** was isolated as a yellow oil by column chromatography using 1:5 ethyl acetate/heptane as eluent. Product yield: 0.0772 g (80% yield). Rf = 0.130 (1:5 ethyl acetate/heptane).

NMR: ^{1}H NMR (300 MHz, $CDCl_3$) δ 7.05 (d, *J* = 8.8, 0.7 Hz, 2H), 6.73 (d, *J* = 8.6 Hz, 2H), 3.76 (t, *J* = 5.9 Hz, 2H), 3.41 – 3.25 (m, 4H), 2.26 (s, 3H), 2.18 (b, 1H), 1.86-1.78 (p, *J* = 6.7, 5.8 Hz, 2H), 1.11 (t, *J* = 7.0 Hz, 3H). ^{13}C NMR (75 MHz, $CDCl_3$) δ 129.93, 114.73, 61.80, 48.89, 46.27, 30.13, 20.40, 12.10.

HRMS: calcd for $C_{12}H_{19}NO$ ([M+H]$^+$): 193.1461, found: 193.1466.

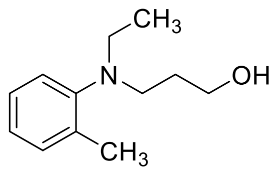

3-(Ethyl(o-tolyl)amino)propan-1-ol (3g): The product **3g** was isolated as a yellow oil by column chromatography using 1:5 ethyl acetate/heptane as eluent. Product yield: 0.0125 g (13% yield). Rf = 0.195 (1:5 ethyl acetate/heptane).

NMR: ^{1}H NMR (300 MHz, $CDCl_3$) δ 7.25 – 7.07 (m, 3H), 7.02 (td, *J* = 7.2, 1.7 Hz, 1H), 3.75 (t, 2H), 3.43 (b, 1H), 3.16 (t, *J* = 6.2 Hz, 2H), 2.99 (q, *J* = 7.1 Hz, 2H), 2.31 (s, 3H), 1.75-1.67 (q, *J* = 11.4, 5.9 Hz, 2H), 0.99 (t, *J* = 7.1 Hz, 3H). ^{13}C NMR (75 MHz, $CDCl_3$) δ 149.54, 134.92, 131.42, 126.43, 124.21, 122.18, 63.32, 51.86, 48.82, 29.03, 18.51, 11.72.

HRMS: calcd for $C_{12}H_{19}NO$ ($[M+H]^+$): 193.1461, found: 193.1467.

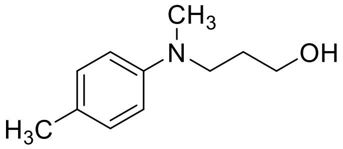

3-(Methyl(p-tolyl)amino)propan-1-ol (3h): The product **3h** was isolated as a yellow oil by column chromatography using 1:5 ethyl acetate/heptane as eluent. Product yield: 0.0818 g (91% yield). Rf = 0.139 (1:5 ethyl acetate/heptane).

NMR: ^{1}H NMR (300 MHz, $CDCl_3$) δ 7.07 (d, *J* = 8.1 Hz, 2H), 6.74 (d, *J* = 8.7 Hz, 2H), 3.74 (t, *J* = 6.0 Hz, 2H), 3.40 (t, *J* = 6.8 Hz, 2H), 2.89 (s, 3H), 2.28 (s, 3H), 2.25 (b, 1H), 1.88-1.79 (p, *J* = 12.7, 6.2 Hz, 2H). ^{13}C NMR (75 MHz, $CDCl_3$) δ 147.93, 129.83, 126.73, 114.00, 61.49, 51.28, 39.01, 29.55, 20.36.

HRMS: calcd for $C_{11}H_{17}NO$ ($[M+H]^+$): 179.1304, found: 179.1304.

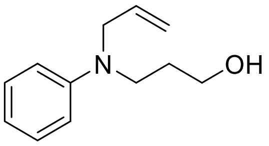

3-(Allyl(phenyl)amino)propan-1-ol (3i): The products **3i** was isolated as a colorless oil by column chromatography using 1:5 ethyl acetate/heptane as eluent. Product yield: 0.0502 g (53% yield). Rf = 0.126 (1:5 ethyl acetate/heptane).

NMR: ^{1}H NMR (400 MHz, $CDCl_3$) δ 7.33 – 7.23 (m, 2H), 6.85 – 6.72 (m, 3H), 5.96-5.87 (ddt, *J* = 17.1, 10.1, 5.0 Hz, 1H), 5.26 – 5.18 (m, 2H), 3.99 (dt, *J* = 5.0, 1.8 Hz, 2H), 3.79 (t, *J* = 6.0 Hz, 2H), 3.50 (t, 2H), 1.94-1.88 (p, 3H). ^{13}C NMR (101 MHz, $CDCl_3$) δ 148.51, 134.19, 129.29, 116.61, 116.33, 112.90, 60.94, 53.70, 47.82, 30.26.

HRMS: calcd for $C_{12}H_{17}NO$ ($[M+H]^+$): 191.1304, found: 191.1305.

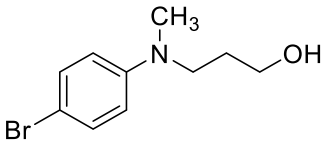

3-((4-Bromophenyl)(methyl)amino)propan-1-ol (3j): The product **3j** was isolated as a colorless oil by column chromatography using 1:5 ethyl acetate/heptane as eluent. Product yield: 0.0928 g (76% yield). Rf = 0.075 (1:5 ethyl acetate/heptane).

NMR: ^{1}H NMR (400 MHz, $CDCl_3$) δ 7.33 – 7.26 (m, 2H), 6.61 (d, *J* = 9.1 Hz, 2H), 3.71 (t, *J* = 6.0 Hz, 2H), 3.42 (t, *J* = 7.0 Hz, 2H), 2.90 (s, 3H), 1.82 (p, 2H), 1.69 (b, 1H). ^{13}C NMR (101 MHz, $CDCl_3$) δ 148.56, 131.94, 114.34, 108.50, 60.77, 49.95, 38.63, 29.61.

HRMS: calcd for $C_{10}H_{14}NOBr$ ($[M+H]^+$): 243.0253, found: 243.0250.

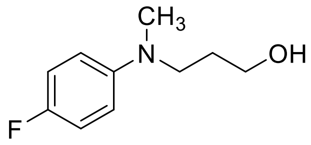

3-((4-Fluorophenyl)(methyl)amino)propan-1-ol (3k): The product **3k** was isolated as a colorless oil by column chromatography using 2:5 ethyl acetate/heptane as eluent. Product yield: 0.0801 g (87% yield). Rf = 0.063 (1:5 ethyl acetate/heptane).

NMR: ^{1}H NMR (400 MHz, $CDCl_3$) δ 6.94 (t, *J* = 8.5 Hz, 2H), 6.72 (dd, *J* = 9.1, 4.3 Hz, 2H), 3.72 (t, *J* = 5.8 Hz, 2H), 3.36 (t, *J* = 6.7 Hz, 2H), 2.86 (s, 3H), 2.45 (b, 1H), 1.80 (t, *J* = 6.2 Hz, 2H). ^{13}C NMR (101 MHz, $CDCl_3$) δ 157.03, 154.69, 146.67, 146.65, 115.70, 115.48, 114.87, 114.80, 61.29, 51.52, 39.24, 29.42. ^{19}F NMR (376 MHz, $CDCl_3$) δ -128.23.

HRMS: calcd for $C_{10}H_{14}NOF$ ($[M+H]^+$): 183.1053, found: 183.1054.

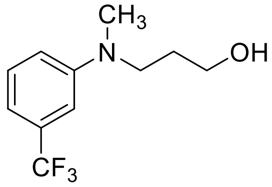

3-(Methyl(3-(trifluoromethyl)phenyl)amino)propan-1-ol (3l): The product **3l** was isolated as a colorless oil by column chromatography using 1:5 ethyl acetate/heptane as eluent. Product yield: 0.0601 g (51% yield). Rf = 0.062 (1:5 ethyl acetate/heptane).

NMR: ^{1}H NMR (400 MHz, $CDCl_3$) δ 7.30 (td, *J* = 8.2, 0.9 Hz, 1H), 6.95 – 6.85 (m, 3H), 3.72 (t, *J* = 6.0 Hz, 2H), 3.49 (t, *J* = 7.0 Hz, 2H), 2.97 (s, 3H), 1.85 (p, 2H), 1.75 (b, 1H). ^{13}C NMR (101 MHz, $CDCl_3$) δ 149.54, 132.03, 131.72, 131.41, 131.10, 129.67, 126.02, 123.31, 115.30, 112.67, 108.53, 60.53, 49.54, 38.48, 29.70. ^{19}F NMR (376 MHz, $CDCl_3$) δ -62.70.

HRMS: calcd for $C_{11}H_{14}NOF_3$ ($[M+H]^+$): 233.1022, found: 233.1026.

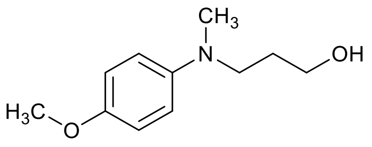

3-((4-Methoxyphenyl)(methyl)amino)propan-1-ol (3m): The product **3m** was isolated as a yellow oil by column chromatography using 1:3 ethyl acetate/heptane as eluent. Product yield: 0.0905 g (96% yield). Rf = 0.270 (1:1 ethyl acetate/heptane).

NMR: ^{1}H NMR (300 MHz, CD_3OD) δ 6.67 – 6.61 (m, 4H), 3.55 (s, 3H), 3.44 (t, *J* = 6.2 Hz, 2H), 3.13 (t, *J* = 7.3 Hz, 2H), 2.65 (s, 3H), 1.57 (p, *J* = 6.5 Hz, 2H). ^{13}C NMR (75 MHz, CD_3OD) δ 153.72, 145.88, 116.87, 115.60, 60.88, 56.05, 52.27, 39.75, 30.37.

HRMS: calcd for $C_{11}H_{25}NO$ ($[M+H]^+$): 196.1337, found: 196.1336.

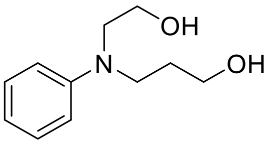

3-((Hydroxyethyl)(phenyl)amino)propan-1-ol (3n): The product **3n** was isolated as a yellow oil by column chromatography using 1:3 ethyl acetate/heptane as eluent. Product yield: 0.0320 g (33% yield). Rf = 0.092 (1:1 ethyl acetate/heptane).

NMR: ^{1}H NMR (300 MHz, CD_3OD) δ 7.17 – 7.04 (m, 2H), 6.70 – 6.59 (m, 3H), 3.70 – 3.53 (m, 6H), 3.26 (t, *J* = 5.6 Hz, 2H), 1.79 (p, *J* = 6.3 Hz, 2H). ^{13}C NMR (75 MHz, CD_3OD) δ 150.03, 130.02, 118.38, 114.32, 70.45, 68.98, 60.01, 44.79, 33.62.

HRMS: calcd for $C_{11}H_{18}NO_2$ ($[M+H]^+$): 196.1338, found: 196.1342.ea

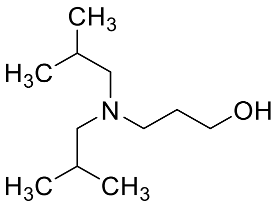

3-(Diisobutylamino)propan-1-ol (3o): The product **3o** was isolated as a yellow oil by column chromatography using 1:5 ethyl acetate/heptane as eluent. Product yield: 0.0660 g (70% yield). Rf = 0.139 (1:1 ethyl acetate/heptane).

NMR: ^{1}H NMR (300 MHz, $CDCl_3$) δ 5.04 (b, 1H), 3.78 (t, 2H), 2.55 (t, 2H), 2.11 (d, *J* = 7.1 Hz, 4H), 1.91 – 1.74 (m, 2H), 1.74 – 1.64 (m, 2H), 0.91 (d, *J* = 6.6 Hz, 12H). ^{13}C NMR (75 MHz, $CDCl_3$) δ 64.69, 63.95, 56.61, 28.08, 26.17, 21.25.

HRMS: calcd for $C_{11}H_{18}NO_2$ ($[M+H]^+$): 188.2014, found: 188.2018.

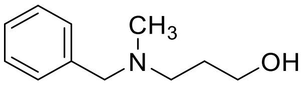

3-(benzyl(methyl)amino)propan-1-ol (3p): The product **3p** was isolated as a yellow oil by column chromatography using 1:1 ethyl acetate/acetone as eluent. Product yield: 0.0743 g (83% yield). Rf = 0.297 (1:1 ethyl acetate/acetone).

NMR: ^{1}H NMR (300 MHz, CD_3OD) δ 7.38 – 7.19 (m, 5H), 3.61 (td, *J* = 6.2, 1.6 Hz, 2H), 3.52 (s, 2H), 2.58 – 2.46 (m, 2H), 2.20 (s, 3H), 1.76 (p, *J* = 6.1 Hz, 2H). ^{13}C NMR (75 MHz, CD_3OD) δ 139.10, 130.55, 129.33, 128.36, 63.17, 62.08, 55.92, 42.27, 30.43.

HRMS: calcd for $C_{11}H_{17}NO$ ($[M+H]^+$): 180.1388, found: 180.1384.

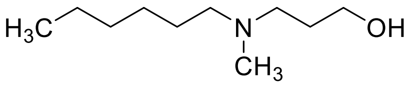

3-(Hexyl(methyl)amino)propan-1-ol (3q): The product **3q** was isolated as a yellow oil by column chromatography using 2:3 ethyl acetate/acetone as eluent. Product yield: 0.0497 g (58% yield). Rf = 0.133 (1:10 methanol/acetone).

NMR: ^{1}H NMR (300 MHz, $CDCl_3$) δ 3.77 (t, *J* = 5.2 Hz, 2H), 2.56 (t, *J* = 5.7 Hz, 2H), 2.38 – 2.27 (m, 2H), 2.21 (s, 3H), 1.73 – 1.60 (m, 2H), 1.44 (q, *J* = 7.4 Hz, 2H), 1.26 (q, *J* = 3.6, 2.4 Hz, 6H), 0.93 – 0.80 (m, 3H). ^{13}C NMR (75 MHz, $CDCl_3$) δ 64.93, 58.75, 58.39, 42.03, 31.87, 27.81, 27.32, 27.11, 22.69, 14.14.

HRMS: calcd for $C_{10}H_{23}NO$ ($[M+H]^+$): 174.1858, found: 174.1858.

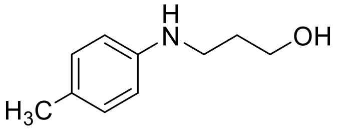

Product 3r

3-(p-Tolylamino)propan-1-ol (3r): The product **3r** was isolated as a yellow oil by column chromatography using 2:5 ethyl acetate/heptane as eluent. Product yield: 0.0244 g (30% yield). Rf = 0.333 (ethyl acetate).

NMR: ^{1}H NMR (300 MHz, CD_3OD) δ 6.98 – 6.87 (m, 2H), 6.63 – 6.52 (m, 2H), 3.67 (t, *J* = 6.2 Hz, 2H), 3.14 (t, *J* = 7.0 Hz, 2H), 2.19 (s, 3H), 1.81 (p, 2H). ^{13}C NMR (75 MHz, CD_3OD) δ 147.89, 130.46, 127.48, 114.64, 61.06, 42.56, 33.10, 20.49.

HRMS: calcd for $C_{10}H_{15}NO$ ($[M+H]^+$): 166.1232, found: 166.1232.

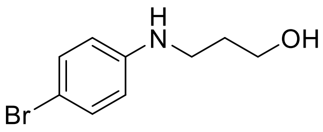

3-((4-bromophenyl)amino)propan-1-ol (3s): The product **3s** was isolated as a yellow oil by column chromatography using 1:3 ethyl acetate/heptane as eluent. Product yield: 0.0236 g (21% yield). Rf = 0.135 (1:3 ethyl acetate/heptane).

NMR: ^{1}H NMR (300 MHz, CD_3OD) δ 7.22 – 7.12 (m, 2H), 6.59 – 6.48 (m, 2H), 3.67 (t, *J* = 6.2 Hz, 2H), 3.14 (t, *J* = 6.9 Hz, 2H), 1.81 (p, 2H). ^{13}C NMR (75 MHz, CD_3OD) δ 149.60, 132.66, 115.33, 108.76, 60.85, 41.66, 32.97.

HRMS: calcd for $C_9H_{12}NOBr$ ($[M+H]^+$): 230.0180, found: 230.0184.

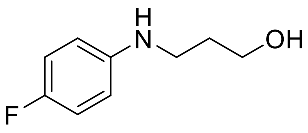

3-((4-Fluorophenyl)amino)propan-1-ol (3t): The product **3t** was isolated as a yellow oil by column chromatography using 1:3 ethyl acetate/heptane as eluent. Product yield: 0.0163 g (19% yield). Rf = 0.122 (1:3 ethyl acetate/heptane).

NMR: ^{1}H NMR (300 MHz, CD_3OD) δ 6.92 – 6.77 (m, 2H), 6.68 – 6.55 (m, 2H), 3.67 (t, *J* = 6.3 Hz, 2H), 3.14 (t, *J* = 7.0 Hz, 2H), 1.81 (p, *J* = 6.4 Hz, 2H). ^{13}C NMR (75 MHz, CD_3OD) δ 158.58, 155.49, 146.85, 116.33, 116.04, 115.03, 114.94, 60.97, 42.64, 33.04.

HRMS: calcd for $C_9H_{12}NOF$ ($[M+H]^+$): 170.0981, found: 170.0982.

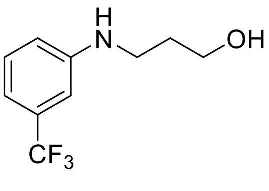

3-((3-(Trifluoromethyl)phenyl)amino)propan-1-ol (3u): The product **3u** was isolated as a yellow oil by column chromatography using 1:3 ethyl acetate/heptane as eluent. Product yield: 0.0220 g (20% yield). Rf = 0.164 (1:3 ethyl acetate/heptane).

NMR: ^{1}H NMR (300 MHz, CD_3OD) δ 7.29 – 7.17 (m, 1H), 6.85 – 6.76 (m, 3H), 3.69 (t, *J* = 6.2 Hz, 2H), 3.20 (t, *J* = 6.9 Hz, 2H), 1.85 (p, 2H). ^{13}C NMR (75 MHz, CD_3OD) δ 150.88, 132.13, 130.61, 127.84, 116.62, 113.33, 113.28, 109.42, 109.36, 60.77, 41.35, 32.92. ^{19}F NMR (282 MHz, CD_3OD) δ -64.38.

HRMS: calcd for $C_{10}H_{12}F_3NO$ ($[M+H]^+$): 220.0949, found: 220.0952.

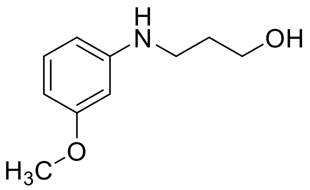

Product 3v

3-((3-Methoxyphenyl)amino)propan-1-ol (3v): The product **3v** was isolated as a yellow oil by column chromatography using 1:3 ethyl acetate/heptane as eluent. Product yield: 0.0193 g (21% yield). Rf = 0.137 (1:3 ethyl acetate/heptane).

NMR: ^{1}H NMR (300 MHz, CD_3OD) δ 7.05 – 6.93 (m, 1H), 6.29 – 6.13 (m, 3H), 3.72 (s, 3H), 3.67 (t, *J* = 6.3 Hz, 2H), 3.16 (t, *J* = 6.9 Hz, 2H), 1.82 (p, 2H). ^{13}C NMR (75 MHz, CD_3OD) δ 162.22, 151.72, 130.68, 107.10, 103.34, 99.83, 60.97, 55.36, 41.91, 33.10.

HRMS: calcd for $C_{10}H_{15}NO_2$ ($[M+H]^+$): 182.1181, found: 182.1177.

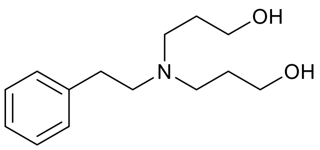

3,3'-(Phenethylazanediyl)bis(propan-1-ol) (3w): The product **3w** was isolated as a yellow oil by column chromatography using 1:1 ethyl acetate/acetone as eluent. Product yield: 0.0693 g (58% yield). Rf = 0.135 (1:1 ethyl acetate/acetone).

NMR: ^{1}H NMR (300 MHz, CD_3OD) δ 7.32 – 7.10 (m, 5H), 3.61 (t, *J* = 6.1 Hz, 4H), 2.82 – 2.54 (m, 8H), 1.71 (p, *J* = 12.6, 6.4 Hz, 4H). ^{13}C NMR (75 MHz, CD_3OD) δ 141.48, 129.71, 129.43, 127.06, 61.90, 57.05, 52.42, 33.91, 30.27.

HRMS: calcd for $C_{14}H_{23}NO_2$ ($[M+H]^+$): 238.1807, found: 238.1810.

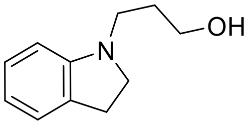

3-(Indolin-1-yl)propan-1-ol (3x): The product **3x** was isolated as a purple oil by column chromatography using 1:5 ethyl acetate/heptane as eluent. Product yield: 0.0504 g (57% yield). Rf = 0.351 (1:1 ethyl acetate/heptane).

NMR: ^{1}H NMR (400 MHz, $CDCl_3$) δ 7.15 – 7.05 (m, 2H), 6.72 (td, *J* = 7.3, 1.0 Hz, 1H), 6.61 (d, *J* = 7.5 Hz, 1H), 3.83 (t, 2H), 3.37 (t, *J* = 8.2 Hz, 2H), 3.23 (t, *J* = 6.6 Hz, 2H), 2.97 (t, *J* = 8.2 Hz, 2H), 1.90 (p, 2H). ^{13}C NMR (101 MHz, $CDCl_3$) δ 130.55, 127.50, 124.69, 118.82, 108.23, 62.30, 53.97, 48.60, 29.95, 28.72.

HRMS: calcd for $C_{11}H_{15}NO$ ([M+H]$^+$): 178.1232, found: 178.1232.

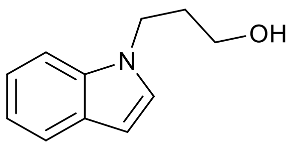

3-(1H-Indol-1-yl)propan-1-ol (3y): The product **3y** was isolated as a yellow oil by column chromatography using 1:1 heptane/ethyl acetate as eluent. Product yield: 0.0317 g (36% yield). Rf = 0.349 (1:1 heptane/ethyl acetate).

NMR: ^{1}H NMR (300 MHz, CD_3OD) δ 7.52 (dt, *J* = 7.8, 1.0 Hz, 1H), 7.30 (dt, *J* = 8.1, 0.9 Hz, 1H), 7.12 – 7.00 (m, 1H), 7.01 – 6.91 (m, 2H), 3.61 (t, *J* = 6.5 Hz, 2H), 2.80 (t, *J* = 7.1 Hz, 2H), 1.92 (p, *J* = 13.7, 6.7 Hz, 2H). ^{13}C NMR (75 MHz, CD_3OD) δ 138.18, 128.77, 122.68, 122.11, 119.33, 119.29, 116.00, 112.07, 62.68, 34.32, 22.34.

HRMS: calcd for $C_{11}H_{13}NO$ ([M+H]$^+$): 176.1075, found: 176.1075.

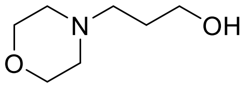

Product 3z

3-Morpholinopropan-1-ol (3z): The product **3z** was isolated by column chromatography as a yellow oil using 1:1 ethyl acetate/acetone as eluent. Product yield: 0.0454 g (63% yield). Rf = 0.122 (1:1 acetone/ethyl acetate).

NMR: ^{1}H NMR (300 MHz, CD_3OD) δ 3.72 – 3.63 (m, 4H), 3.60 (t, *J* = 6.2 Hz, 2H), 2.51 – 2.40 (m, 6H), 1.72 (p, *J* = 6.4 Hz, 2H). ^{13}C NMR (75 MHz, CD_3OD) δ 67.59, 61.59, 57.23, 54.74, 29.81.

HRMS: calcd for $C_7H_{15}NO_2$ ([M+H]$^+$): 146.1181, found: 146.1178.

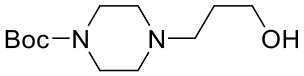

tert-Butyl 4-(3-hydroxypropyl)piperazine-1-carboxylate (3aa): The product **3aa** was isolated as a white solid by column chromatography using 1:10 dichloromethane/ethyl acetate as eluent. Product yield: 0.1016 g (83% yield). Obtained. Rf = 0.189 (1:10 methanol/ethyl acetate).

NMR: ^{1}H NMR (300 MHz, CD_3OD) δ 3.62 (t, *J* = 6.2 Hz, 2H), 3.43 (t, *J* = 5.1 Hz, 4H), 2.54 – 2.39 (m, 6H), 1.81 – 1.63 (m, 2H), 1.45 (s, 9H). ^{13}C NMR (75 MHz, CD_3OD) δ 156.33, 81.26, 61.57, 56.76, 54.01, 30.12, 28.61.

HRMS: calcd for $C_{12}H_{24}N_2O_3$ ([M+H]$^+$): 245.1865, found: 245.1867.

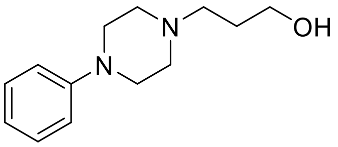

3-(4-Phenylpiperazin-1-yl)propan-1-ol (3ab): The product **3ab** was isolated as a white solid by column chromatography using 1:1 ethyl acetate/acetone as eluent. Product yield: 0.0995 g (90% yield). Rf = 0.338 (1:1 acetone/ethyl acetate).

NMR: ^{1}H NMR (300 MHz, CD_3OD) δ 7.27 – 7.14 (m, 2H), 6.94 (d, *J* = 7.8 Hz, 2H), 6.82 (t, *J* = 7.3 Hz, 1H), 3.62 (t, *J* = 6.2 Hz, 2H), 3.15 (t, 4H), 2.62 (t, 4H), 2.50 (t, 2H), 1.76 (q, 2H). ^{13}C NMR (75 MHz, CD_3OD) δ 152.59, 130.05, 121.17, 117.45, 61.73, 56.89, 54.26, 50.23, 30.10.

HRMS: calcd for $C_{13}H_{20}N_2O$ ($[M+H]^+$): 221.1654, found: 221.1654.

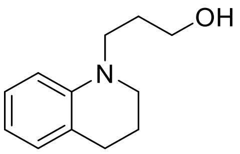

3-(3,4-Dihydroquinolin-1(2H)-yl)propan-1-ol (3ac): The product **3ac** was isolated as a yellow oil by column chromatography using 1:1 ethyl acetate/heptane as eluent. Product yield: 0.0901 g (94% yield). Rf = 0.405 (1:1 heptane/ethyl acetate).

NMR: ^{1}H NMR (300 MHz, $CDCl_3$) δ 7.09 (ddd, *J* = 8.2, 7.3, 1.7 Hz, 1H), 6.98 (dq, *J* = 7.3, 1.2 Hz, 1H), 6.68 (dd, *J* = 8.3, 1.1 Hz, 1H), 6.62 (td, *J* = 7.3, 1.2 Hz, 1H), 3.76 (t, *J* = 6.0 Hz, 2H), 3.41 (t, *J* = 7.0 Hz, 2H), 3.31 (t, 2H), 2.79 (t, *J* = 6.4 Hz, 2H), 2.15 (b, 1H), 2.05 – 1.93 (m, 2H), 1.88 (p, *J* = 6.3 Hz, 2H). ^{13}C NMR (75 MHz, $CDCl_3$) δ 145.47, 129.28, 127.15, 122.71, 115.92, 111.05, 61.09, 49.61, 48.74, 29.36, 28.17, 22.24.

HRMS: calcd for $C_{12}H_{17}NO$ ($[M+H]^+$): 192.1388, found: 192.1388.

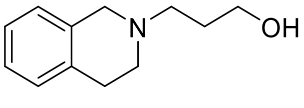

3-(3,4-Dihydroisoquinolin-2(1H)-yl)propan-1-ol (3ad): The product **3ad** was isolated as a yellow oil by column chromatography using 1:1 ethyl acetate/acetone as eluent. Product yield: 0.0623 g (59% yield). Rf = 0.329 (1:1 acetone/ethyl acetate).

NMR: ^{1}H NMR (300 MHz, $CDCl_3$) δ 7.19 – 7.06 (m, 3H), 7.06 – 6.97 (m, 1H), 4.52 (b, 1H), 3.82 (t, *J* = 5.3 Hz, 2H), 3.70 (s, 2H), 2.90 (t, *J* = 5.7 Hz, 2H), 2.85 – 2.71 (m, 4H), 1.82 (p, *J* = 5.3 Hz, 2H). ^{13}C NMR

(75 MHz, $CDCl_3$) δ 134.22, 134.08, 128.64, 126.56, 126.33, 125.77, 64.31, 58.39, 56.47, 50.80, 28.99, 27.71.

HRMS: calcd for $C_{12}H_{17}NO$ ($[M+H]^+$): 192.1388, found: 192.1385.

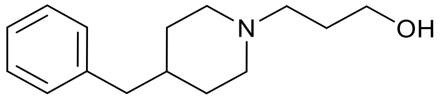

3-(4-Benzylpiperidin-1-yl)propan-1-ol (3ae): The product **3ae** was isolated as a white solid by column chromatography using 1:1 acetone/ethyl acetate as eluent. Product yield: 0.0917 g (79% yield). Rf = 0.194 (1:5 methanol/ethyl acetate).

NMR: ^1H NMR (300 MHz, CD_3OD) δ 7.32 – 7.20 (m, 2H), 7.18 – 7.08 (m, 3H), 3.59 (t, *J* = 6.2 Hz, 2H), 2.92 (d, *J* = 12.0 Hz, 2H), 2.52 (d, *J* = 6.9 Hz, 2H), 2.42 (t, 2H), 1.91 (td, *J* = 11.9, 2.5 Hz, 2H), 1.77 – 1.43 (m, 5H), 1.36 – 1.17 (m, 2H). ^{13}C NMR (75 MHz, CD_3OD) δ 141.64, 130.10, 129.20, 126.88, 61.91, 57.27, 54.88, 43.96, 39.02, 32.80, 30.13.

HRMS: calcd for $C_{15}H_{23}NO$ ($[M+H]^+$): 234.1858, found: 234.1857.

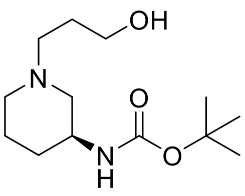

tert-Butyl (S)-(1-(3-hydroxypropyl)piperidin-3-yl)carbamate (3af): The product **3af** was isolated as a yellow oil by column chromatography using 1:1 ethyl acetate/acetone as eluent. Product yield: 0.1143 g (88% yield). Obtained. Rf = 0.567 (1:1 acetone/ethyl acetate).

NMR: ^1H NMR (300 MHz, CD_3OD) δ 3.68 – 3.48 (m, 3H), 2.83 (dd, 2H), 2.46 (t, *J* = 7.5 Hz, 2H), 2.16 (s, 1H), 2.11 – 1.97 (m, 1H), 1.96 – 1.52 (m, 6H), 1.43 (s, 9H), 1.24 (tq, *J* = 11.0, 6.8, 5.5 Hz, 1H). ^{13}C NMR (75 MHz, CD_3OD) δ 157.51, 79.86, 61.70, 59.98, 56.95, 54.39, 31.39, 30.16, 28.78, 24.57.

HRMS: calcd for $C_{13}H_{26}N_2O_3$ ($[M+H]^+$): 259.2021, found: 259.2021.

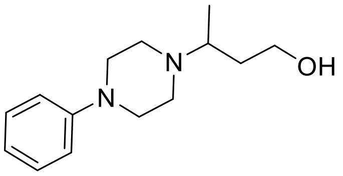

3-(4-Phenylpiperazin-1-yl)butan-1-ol (5a): The product **5a** was isolated as a white solid by column chromatography using ethyl acetate as eluent. Product yield: 0.0967 g (82% yield). Rf = 0.189 (ethyl acetate).

NMR: ^{1}H NMR (300 MHz, CD_3OD) δ 7.31 – 7.16 (m, 2H), 6.96 (dd, *J* = 8.8, 1.1 Hz, 2H), 6.84 (tt, *J* = 7.2, 1.1 Hz, 1H), 3.78 – 3.62 (m, 2H), 3.25 – 3.07 (m, 4H), 2.97 – 2.61 (m, 5H), 1.90 (h, 1H), 1.48 (ddt, *J* = 14.0, 6.5, 5.1 Hz, 1H), 1.08 (d, *J* = 6.6 Hz, 3H). ^{13}C NMR (75 MHz, $CDCl_3$) δ 151.17, 129.08, 119.93, 116.23, 61.29, 49.60, 48.00, 33.76, 13.24.

HRMS: calcd for $C_{14}H_{22}N_2O$ ($[M+H]^+$): 235.1810 found: 235.1805.

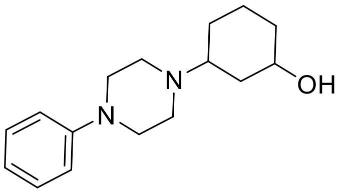

3-(4-Phenylpiperazin-1-yl)cyclohexan-1-ol (5b): The product **5b** was isolated as a white solid by column chromatography using ethyl acetate as eluent. Product yield: 0.0532 g (41% yield). Rf = 0.095 (ethyl acetate).

NMR: ^{1}H NMR (400 MHz, CD_3OD) δ 7.27 – 7.18 (m, 2H), 7.00 – 6.92 (m, 2H), 6.83 (tt, *J* = 7.3, 1.0 Hz, 1H), 3.31 (p, *J* = 1.6 Hz, 1H), 3.17 (t, 4H), 2.83 – 2.71 (m, 5H), 2.03 – 1.87 (m, 2H), 1.81 – 1.24 (m, 6H). ^{13}C NMR (101 MHz, CD_3OD) δ 152.72, 130.04, 121.14, 117.43, 67.68, 59.15, 50.57, 50.09, 35.89, 33.56, 29.20, 20.78.

HRMS: calcd for $C_{16}H_{24}N_2O$ ($[M+H]^+$): 261.1967, found: 261.1963.

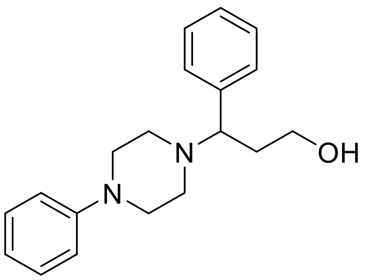

3-Phenyl-3-(4-phenylpiperazin-1-yl)propan-1-ol (5c): The product **5c** was isolated as a white solid by column chromatography using 1:1 ethyl acetate/heptane as eluent. Product yield: 0.0440 g (30% yield). Rf = 0.081 (1:1 ethyl acetate/heptane).

NMR: ^{1}H NMR (300 MHz, CD_3OD) δ 7.41 – 7.22 (m, 5H), 7.25 – 7.12 (m, 2H), 6.91 (dd, 2H), 6.81 (tt, 1H), 3.63 (dd, *J* = 8.6, 6.0 Hz, 1H), 3.59 – 3.36 (m, 2H), 3.12 (td, *J* = 4.5, 2.4 Hz, 4H), 2.61 (t, *J* = 5.0 Hz, 4H), 2.33 (h, *J* = 13.6, 6.6 Hz, 1H), 1.93 (ddt, *J* = 13.6, 8.7, 5.5 Hz, 1H). ^{13}C NMR (75 MHz, CD_3OD) δ 152.67, 139.78, 129.96, 129.26, 128.62, 121.13, 117.44, 68.60, 61.00, 51.25, 50.73, 35.57.

HRMS: calcd for $C_{19}H_{24}N_2O$ ($[M+H]^+$): 297.1967, found: 297.1966.

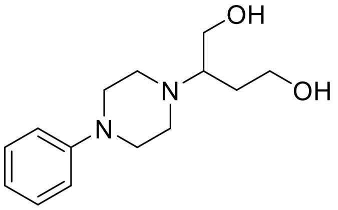

2-(4-Phenylpiperazin-1-yl)butane-1,4-diol (5d): The product **5d** was isolated as a yellow oil by column chromatography using ethyl acetate as eluent. Product yield: 0.0308 g (25% yield). Rf = 0.081 (ethyl acetate).

NMR: ^{1}H NMR (300 MHz, CD_3OD) δ 7.29 – 7.16 (m, 2H), 7.01 – 6.90 (m, 2H), 6.83 (tt, *J* = 7.3, 1.1 Hz, 1H), 3.76 – 3.53 (m, 4H), 3.23 – 2.99 (m, 4H), 2.99 – 2.64 (m, 5H), 1.85 (dq, *J* = 14.1, 7.0 Hz, 1H), 1.58 (dq, *J* = 14.2, 5.6 Hz, 1H). ^{13}C NMR (75 MHz, CD_3OD) δ 152.84, 130.00, 121.11, 117.53, 65.08, 61.98, 51.22, 49.92, 30.49.

HRMS: calcd for $C_{14}H_{22}N_2O_2$ ($[M+H]^+$): 251.1759, found: 251.1763.

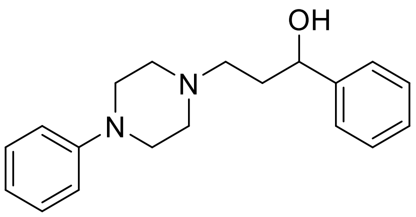

1-Phenyl-3-(4-phenylpiperazin-1-yl)propan-1-ol (5e): The product **5e** was isolated as a yellow solid by column chromatography using 1:20 ether/DCM as eluent. Product yield: 0.0880 g (59% yield). Rf = 0.243 (1:1 ethyl acetate/heptane).

NMR: ^{1}H NMR (300 MHz, CD_3OD) δ 7.41 – 7.29 (m, 4H), 7.29 – 7.16 (m, 3H), 7.01 – 6.90 (m, 2H), 6.84 (tt, *J* = 7.4, 1.0 Hz, 1H), 4.76 (dd, *J* = 7.8, 5.0 Hz, 1H), 3.18 (t, *J* = 5.0 Hz, 4H), 2.75 – 2.55 (m, 5H), 2.48 (ddd, *J* = 12.4, 8.2, 5.6 Hz, 1H), 2.09 – 1.83 (m, 2H). ^{13}C NMR (75 MHz, CD_3OD) δ 152.61, 146.06, 130.04, 129.33, 128.31, 126.92, 121.20, 117.49, 74.52, 56.78, 54.28, 50.35, 36.29.

HRMS: calcd for $C_{19}H_{24}N_2O$ ($[M+H]^+$): 297.1967, found: 297.1966.

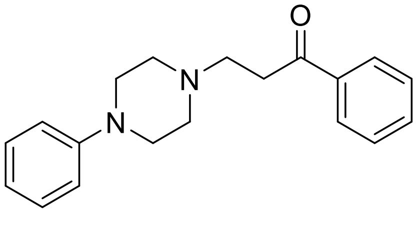

1-Phenyl-3-(4-phenylpiperazin-1-yl)propan-1-one (5e`):** The product **5e` was isolated as a colorless solid by column chromatography using 1:10 ether/DCM as eluent. Product yield: 0.0357 g (24% yield). Rf = 0.378 (1:1 ethyl acetate/heptane).

NMR: ^{1}H NMR (300 MHz, CD_3OD) δ 8.07 – 7.96 (m, 2H), 7.66 – 7.55 (m, 1H), 7.55 – 7.44 (m, 2H), 7.29 – 7.16 (m, 2H), 7.00 – 6.90 (m, 2H), 6.89 – 6.77 (m, 1H), 3.17 (t, 4H), 2.90 – 2.82 (m, 2H), 2.69 (t, 4H). ^{13}C NMR (75 MHz, CD_3OD) δ 200.77, 152.61, 138.22, 134.38, 130.05, 129.79, 129.17, 121.14, 117.44, 54.25, 54.06, 50.21.

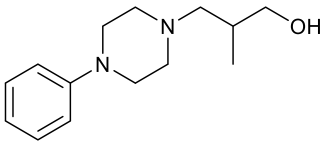

2-Methyl-3-(4-phenylpiperazin-1-yl)propan-1-ol 5f: The product **5f** was isolated as a yellow solid by column chromatography using 1:1 heptane/ethyl acetate as eluent. Product yield: 0.0766 g (65% yield). Rf = 0.192 (1:3 ethyl acetate/heptane).

NMR: ^{1}H NMR (300 MHz, CD_3OD) δ 7.22 (t, J = 7.8 Hz, 2H), 6.94 (d, J = 8.0 Hz, 2H), 6.83 (t, J = 7.3 Hz, 1H), 3.52 (d, J = 6.2 Hz, 2H), 3.13 (d, J = 5.1 Hz, 4H), 2.62 (dq, J = 34.5, 6.1 Hz, 4H), 2.44 (dd, J = 12.4, 8.9 Hz, 1H), 2.31 (dd, J = 12.4, 5.4 Hz, 1H), 2.02 (h, *J* = 6.4 Hz, 1H), 0.89 (d, *J* = 6.6 Hz, 3H). ^{13}C NMR (75 MHz, CD_3OD) δ 152.62, 130.00, 121.04, 117.38, 69.04, 65.02, 54.69, 50.44, 33.00, 15.64.

HRMS: calcd for $C_{14}H_{22}N_2O$ ($[M+H]^+$): 235.1810, found: 235.1812.

6. NMR spectra of isolated hydroamination products

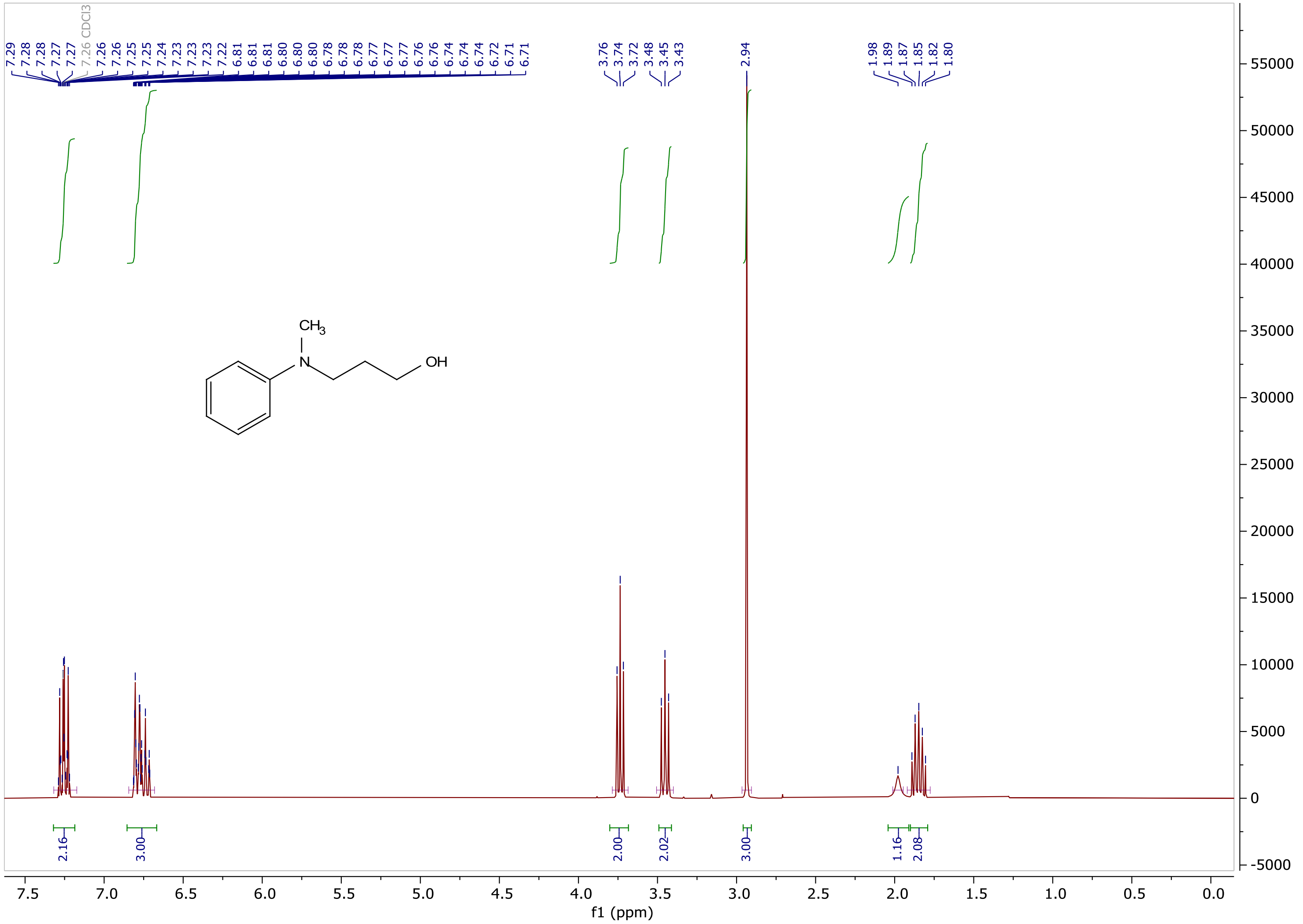

^{1}H NMR (300 MHz, $CDCl_3$) spectrum of compound **3a**.

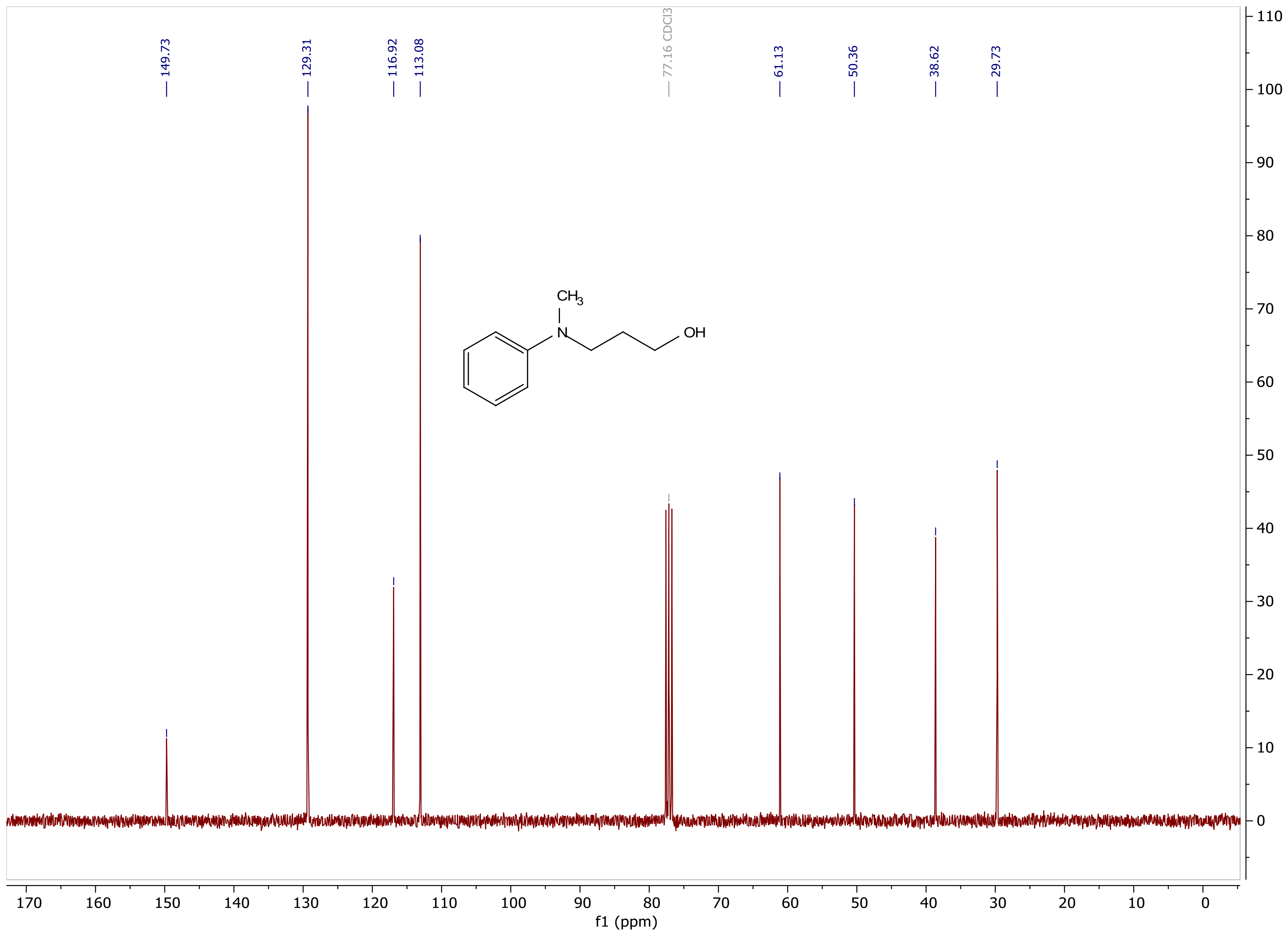

^{13}C NMR (75 MHz, $CDCl_3$) spectrum of compound **3a**.

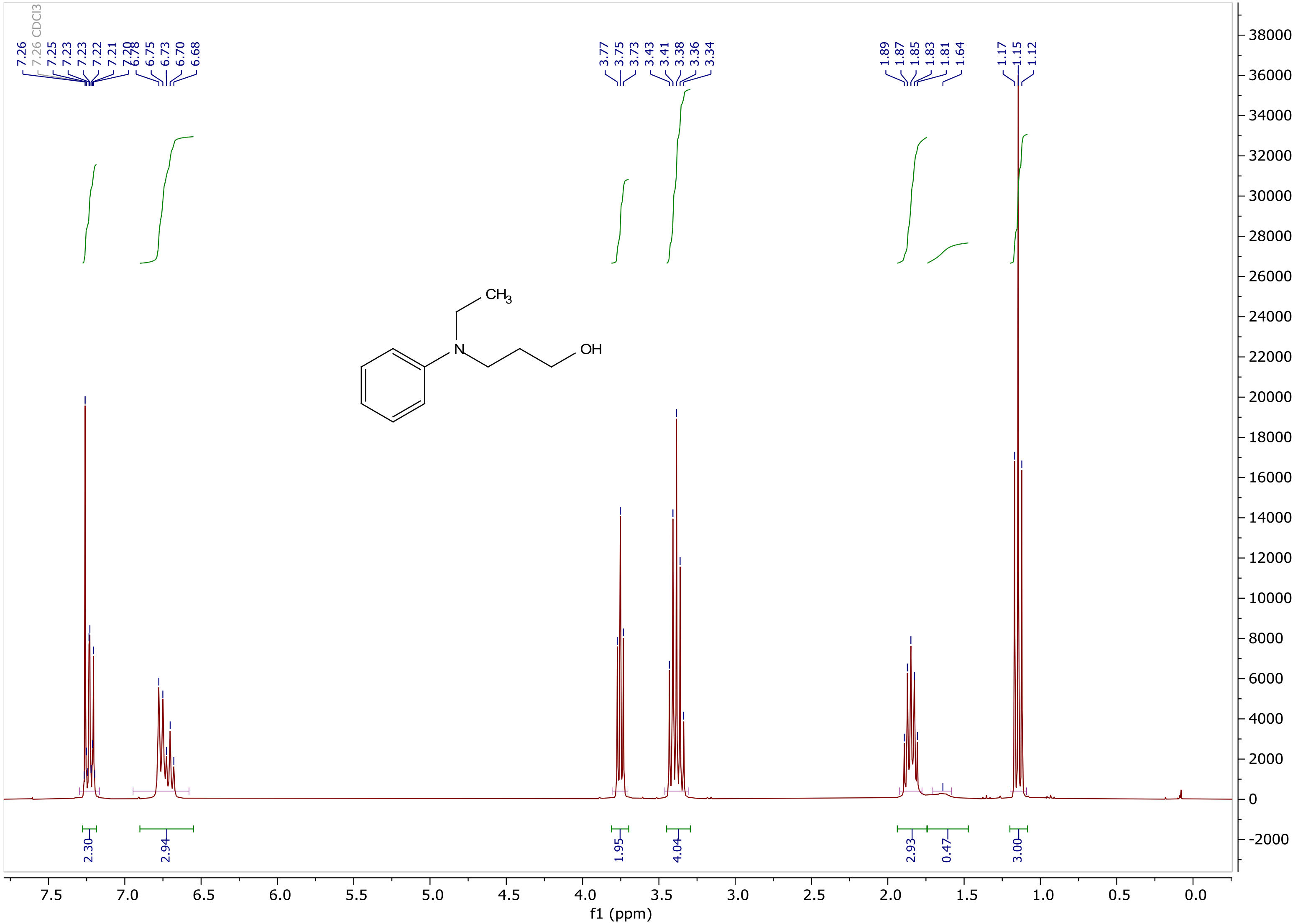

^{1}H NMR (300 MHz, $CDCl_3$) spectrum of compound **3b**.

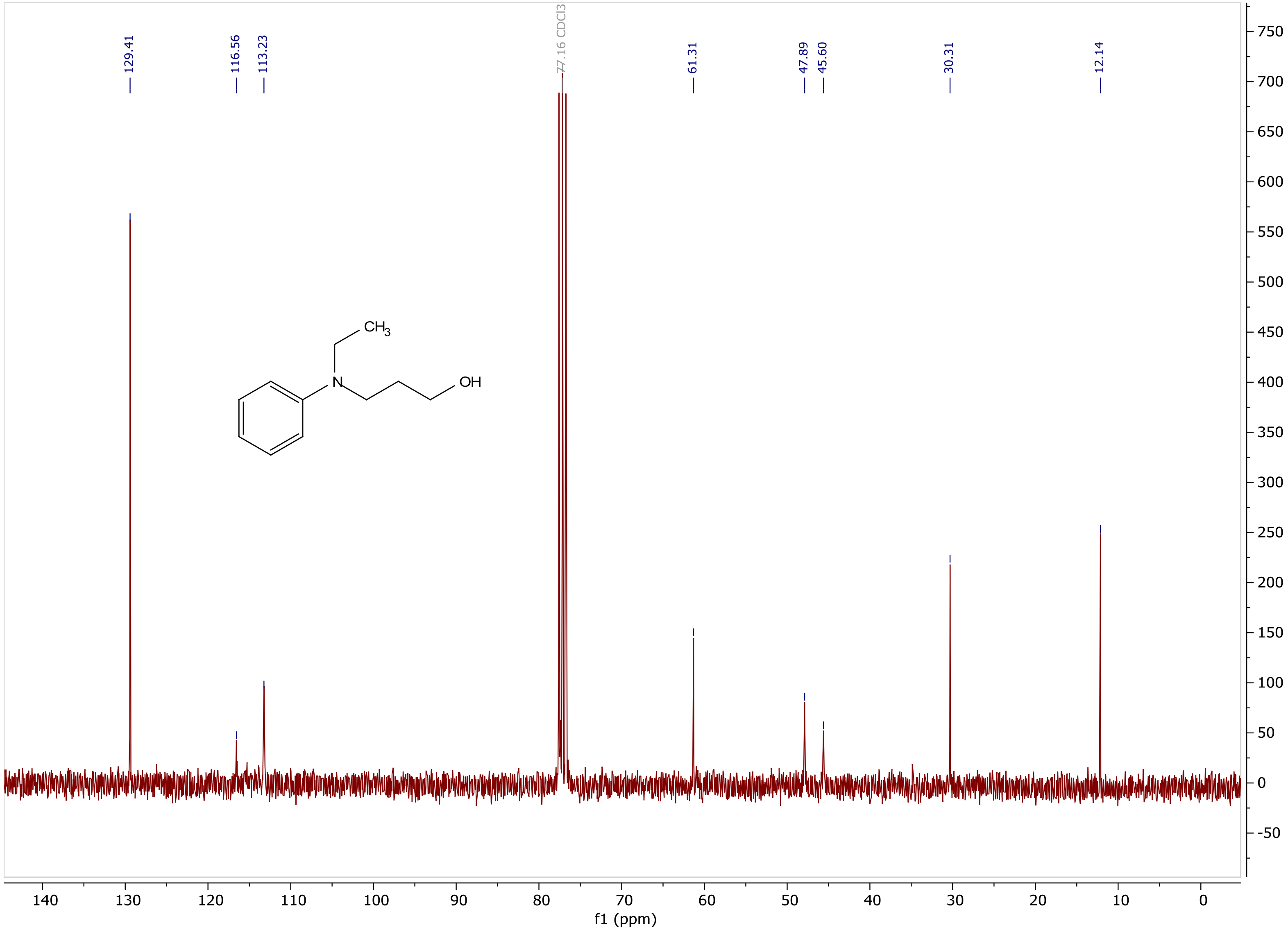

^{13}C NMR (75 MHz, $CDCl_3$) spectrum of compound **3b**.

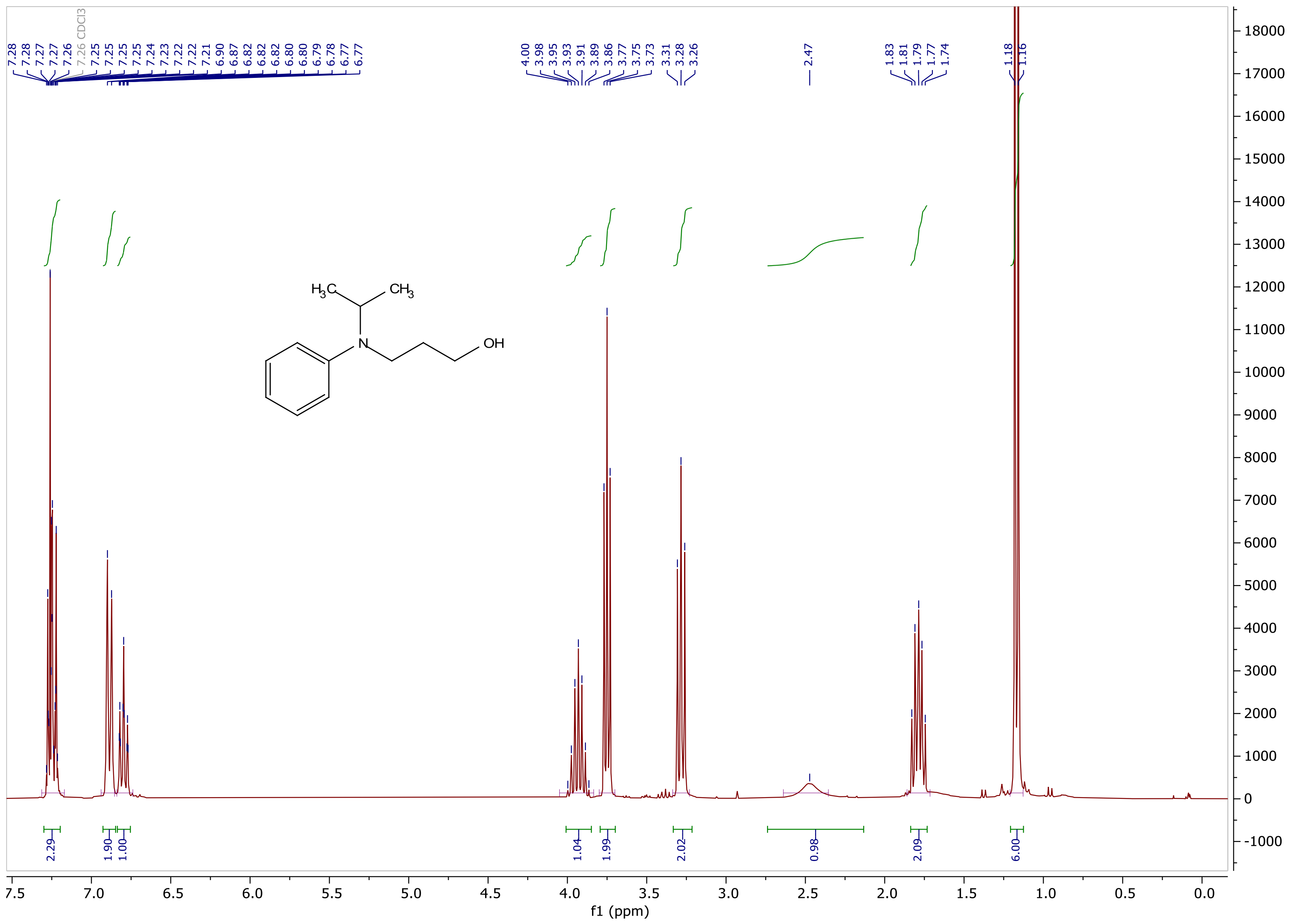

^{1}H NMR (300 MHz, $CDCl_3$) spectrum of compound **3c.**

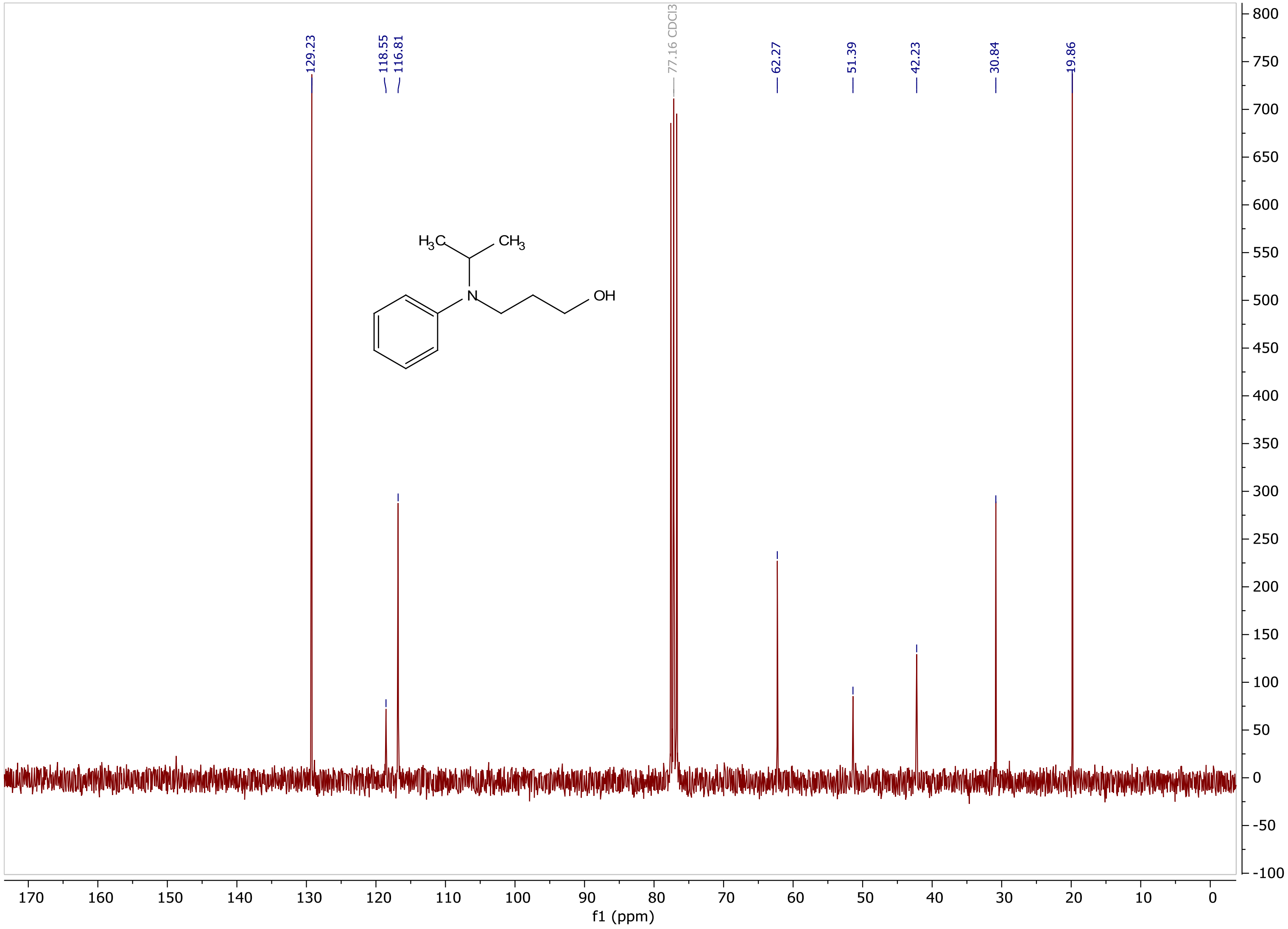

^{13}C NMR (75 MHz, $CDCl_3$) spectrum of compound **3c.**

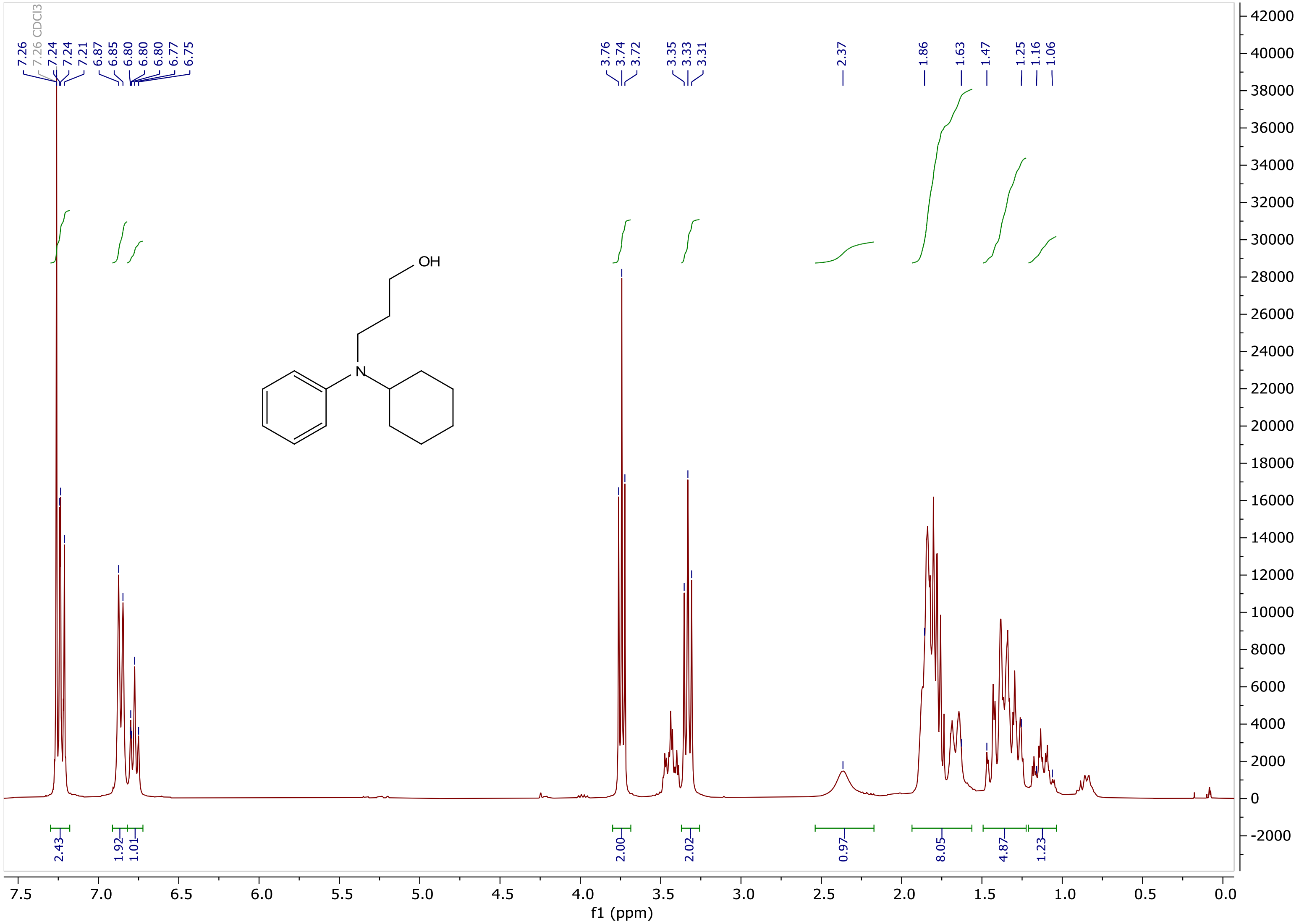

^{1}H NMR (300 MHz, $CDCl_3$) spectrum of compound **3e.**

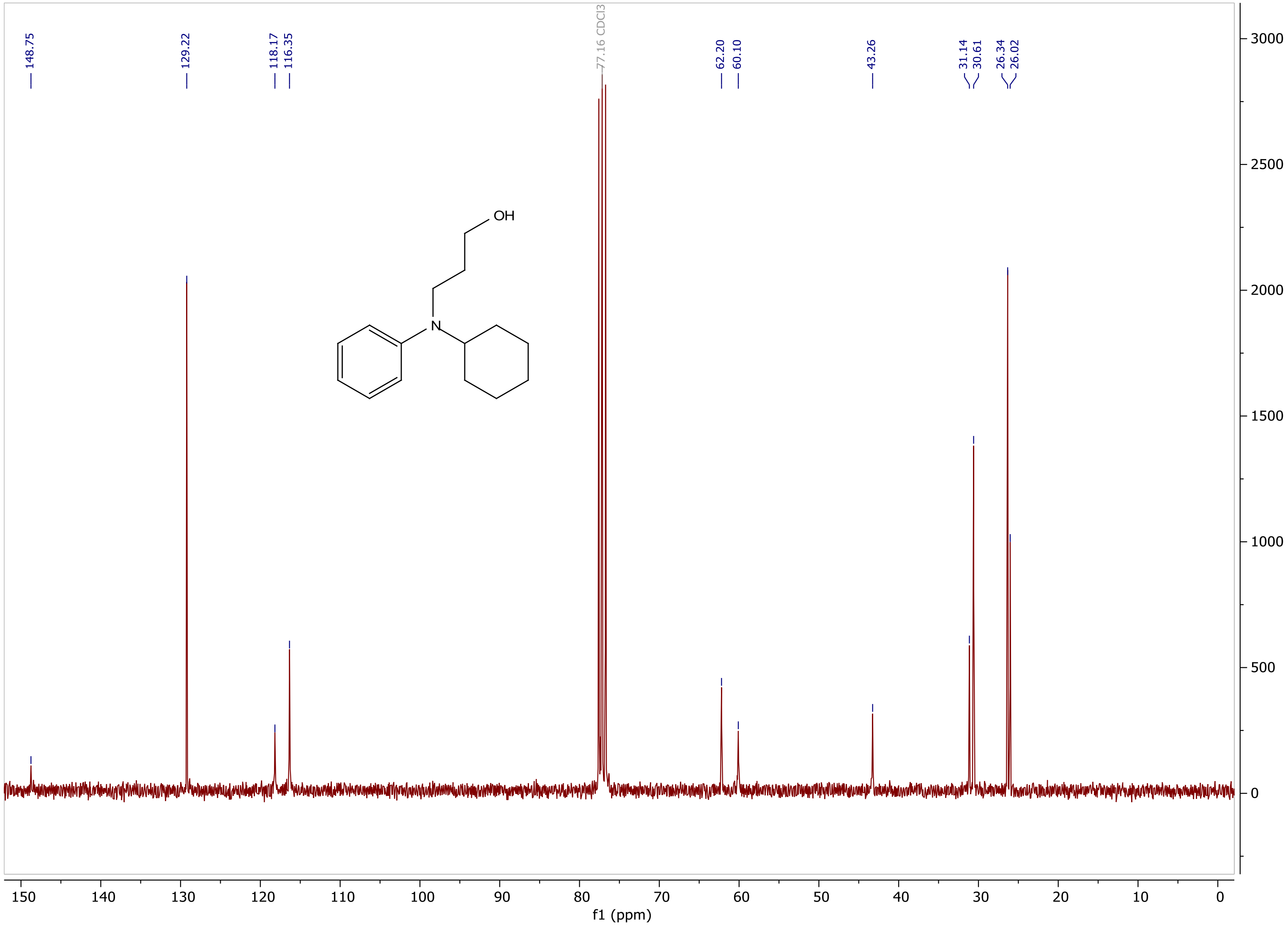

^{13}C NMR (75 MHz, $CDCl_3$) spectrum of compound **3e.**

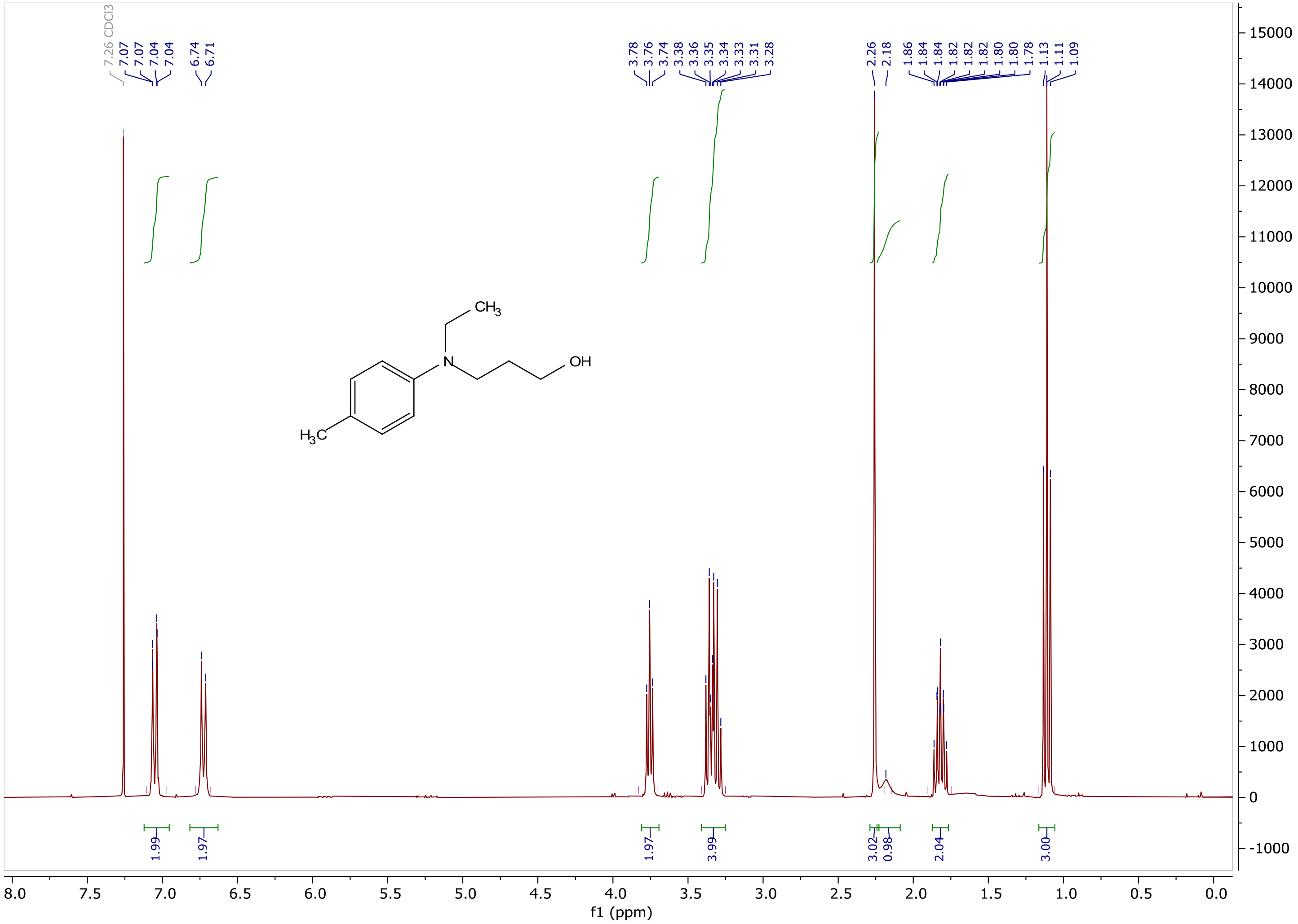

^{1}H NMR (300 MHz, $CDCl_3$) spectrum of compound **3f.**

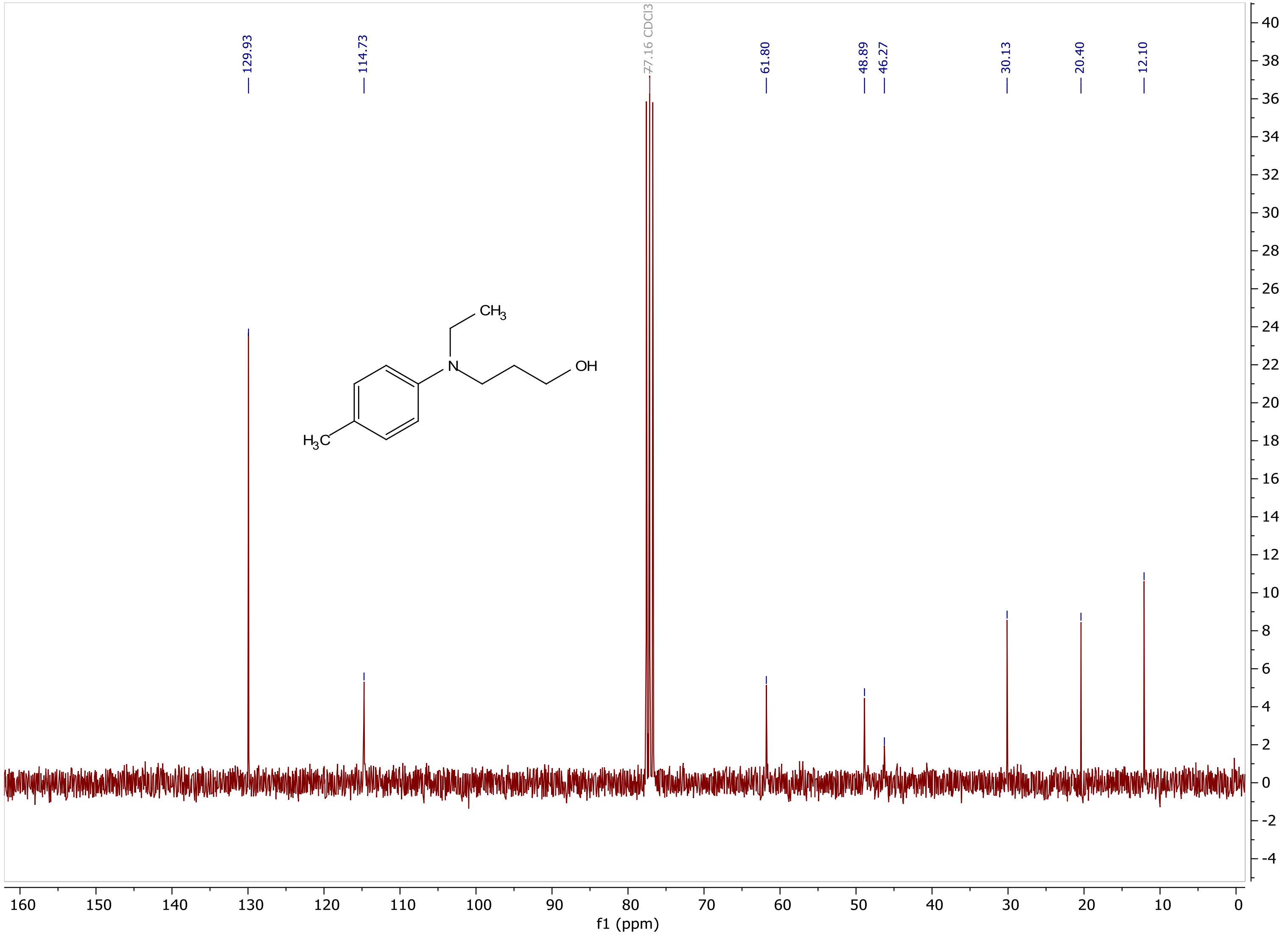

^{13}C NMR (75 MHz, $CDCl_3$) spectrum of compound **3f.**

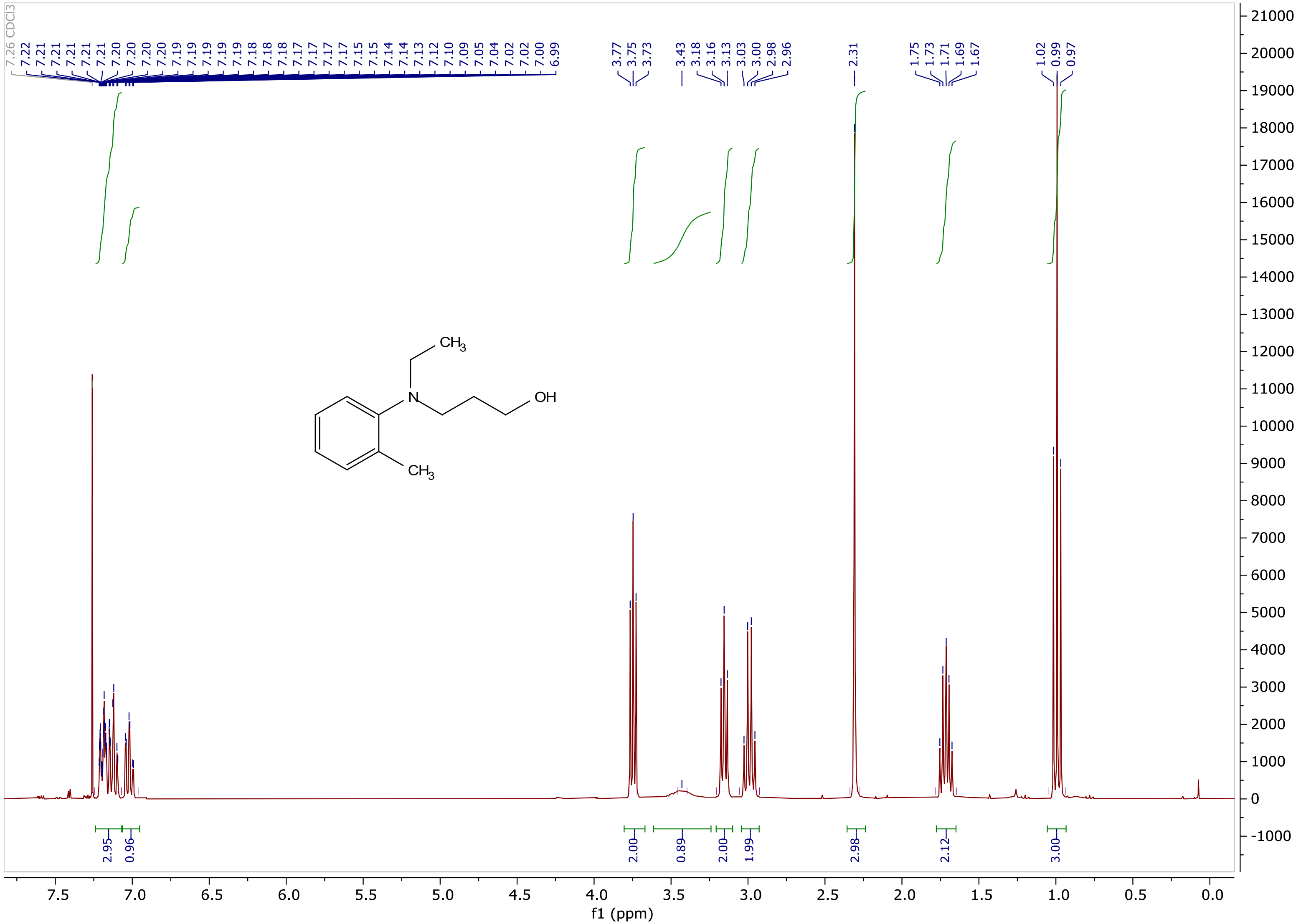

^1H NMR (300 MHz, $CDCl_3$) spectrum of compound **3g.**

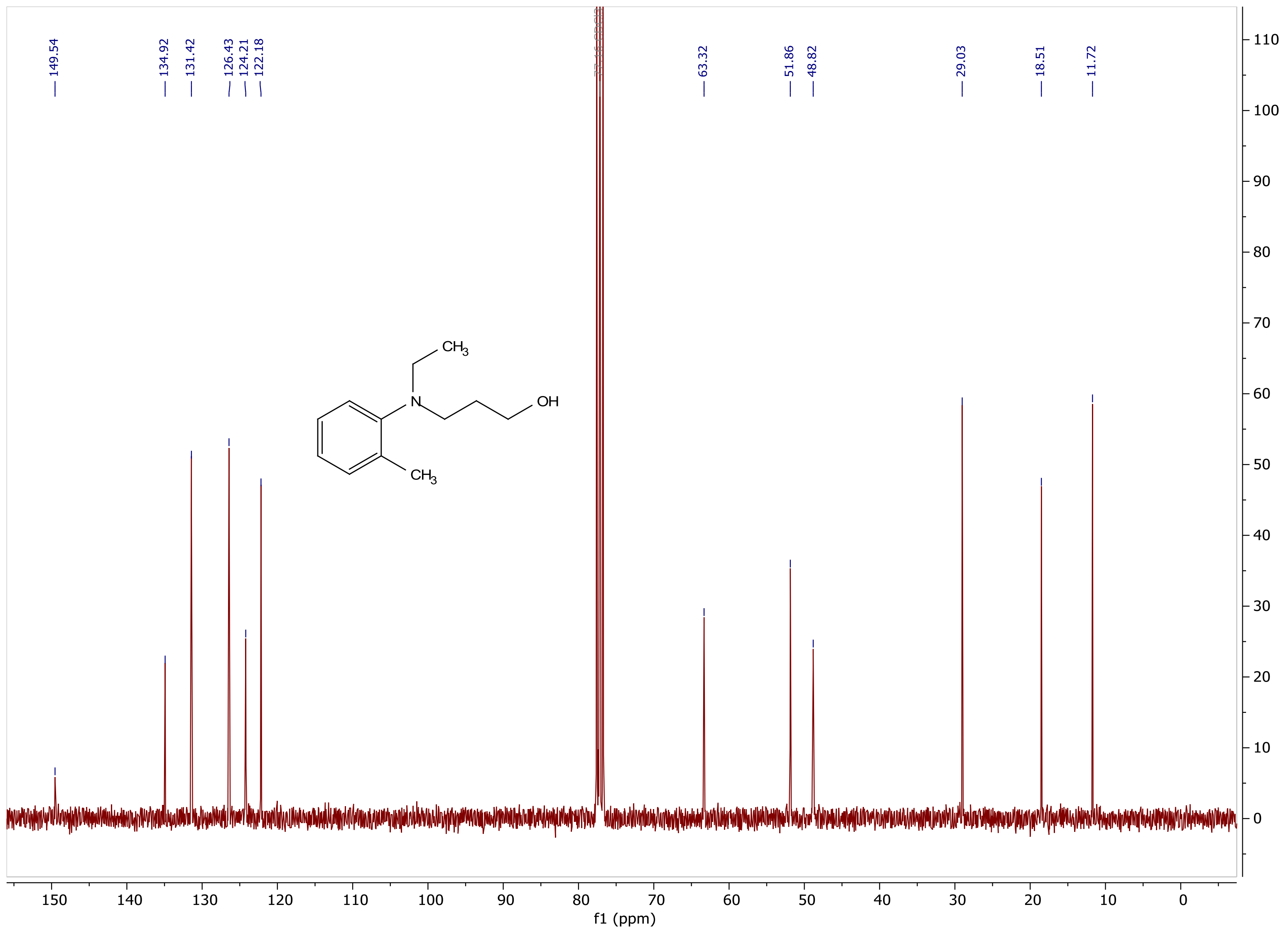

^{13}C NMR (75 MHz, $CDCl_3$) spectrum of compound **3g.**

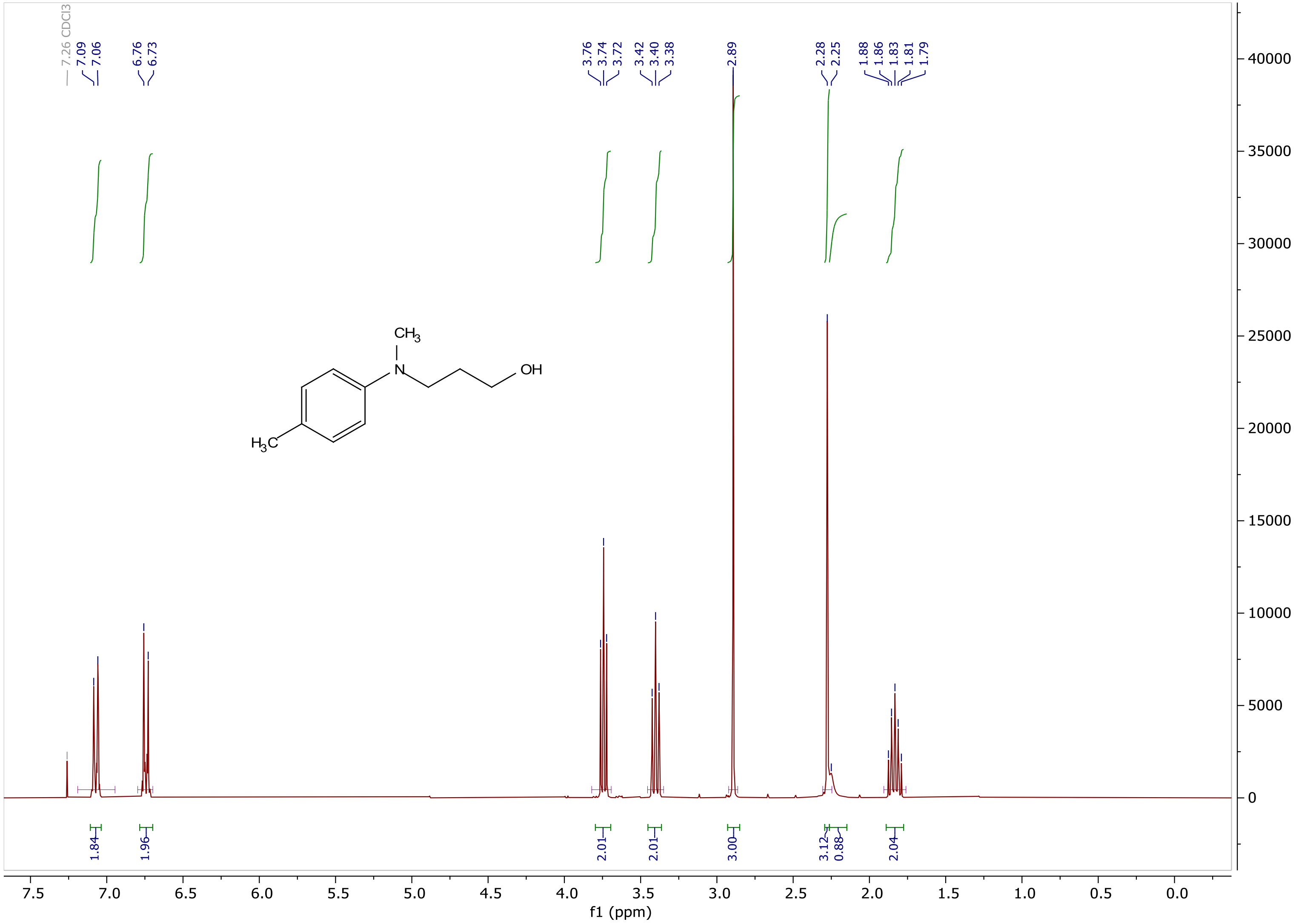

^{1}H NMR (300 MHz, $CDCl_3$) spectrum of compound **3h.**

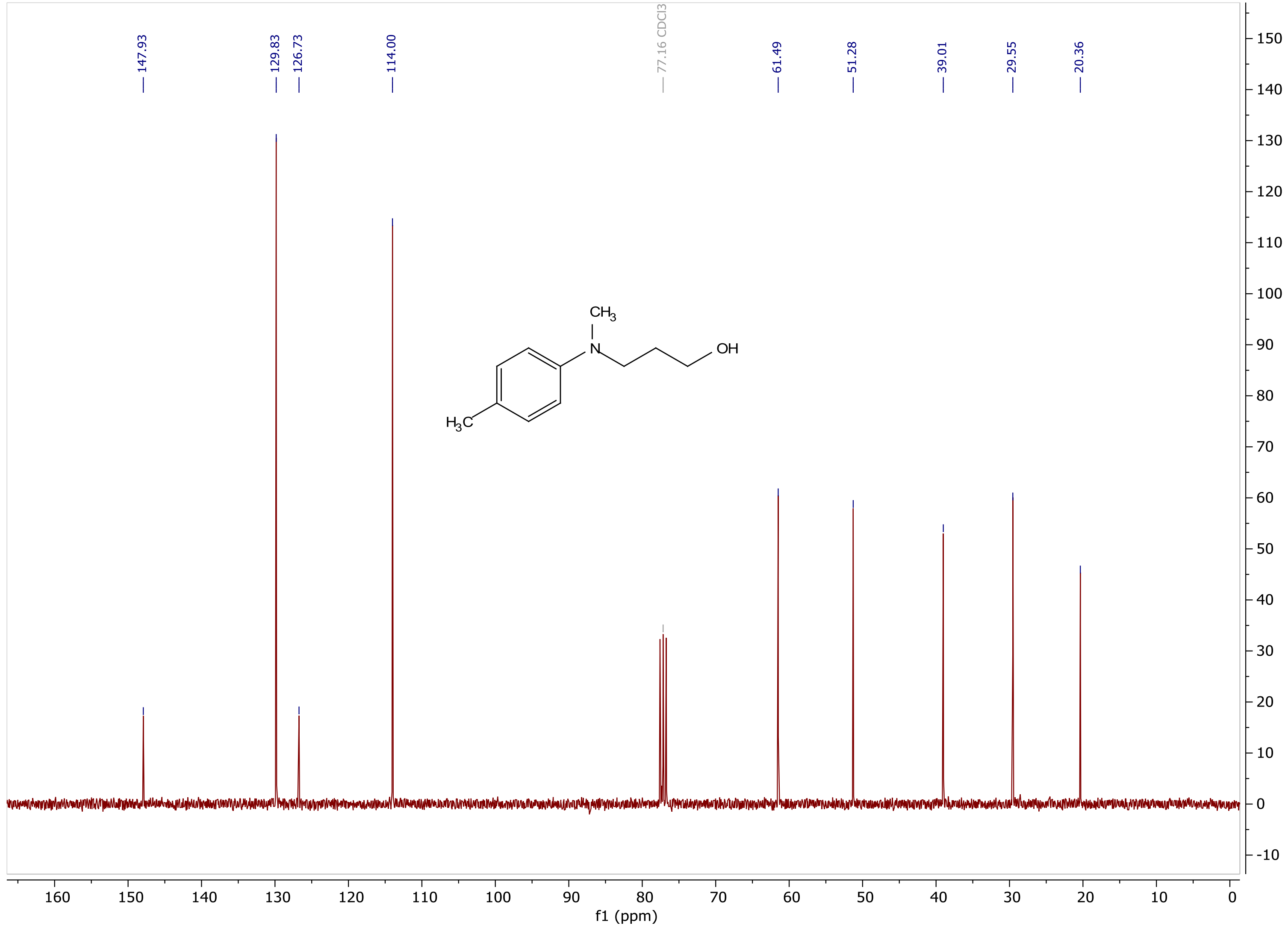

^{13}C NMR (75 MHz, $CDCl_3$) spectrum of compound **3h.**

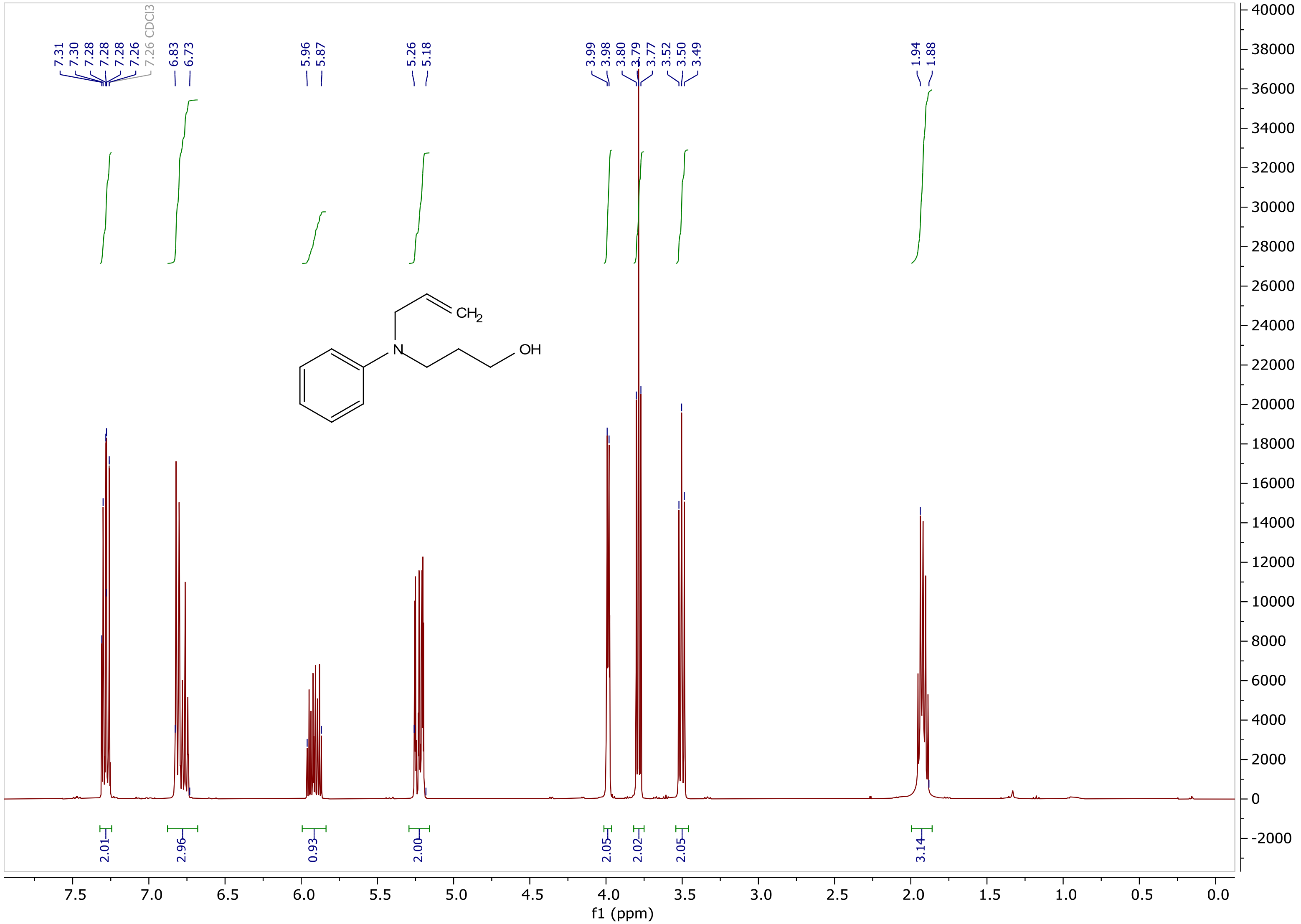

^{1}H NMR (400 MHz, $CDCl_3$) spectrum of compound **3i.**

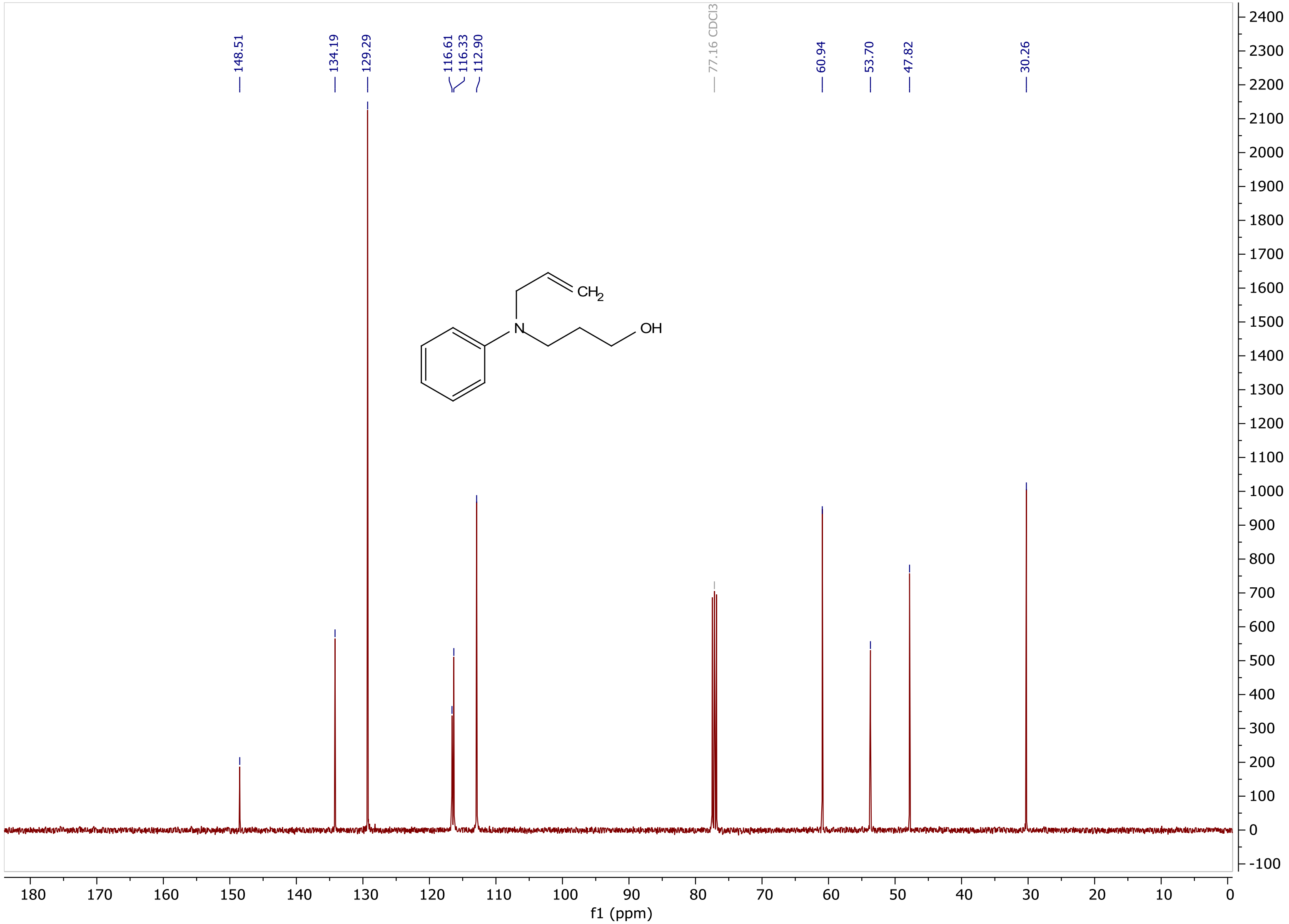

^{13}C NMR (101 MHz, $CDCl_3$) spectrum of compound **3i.**

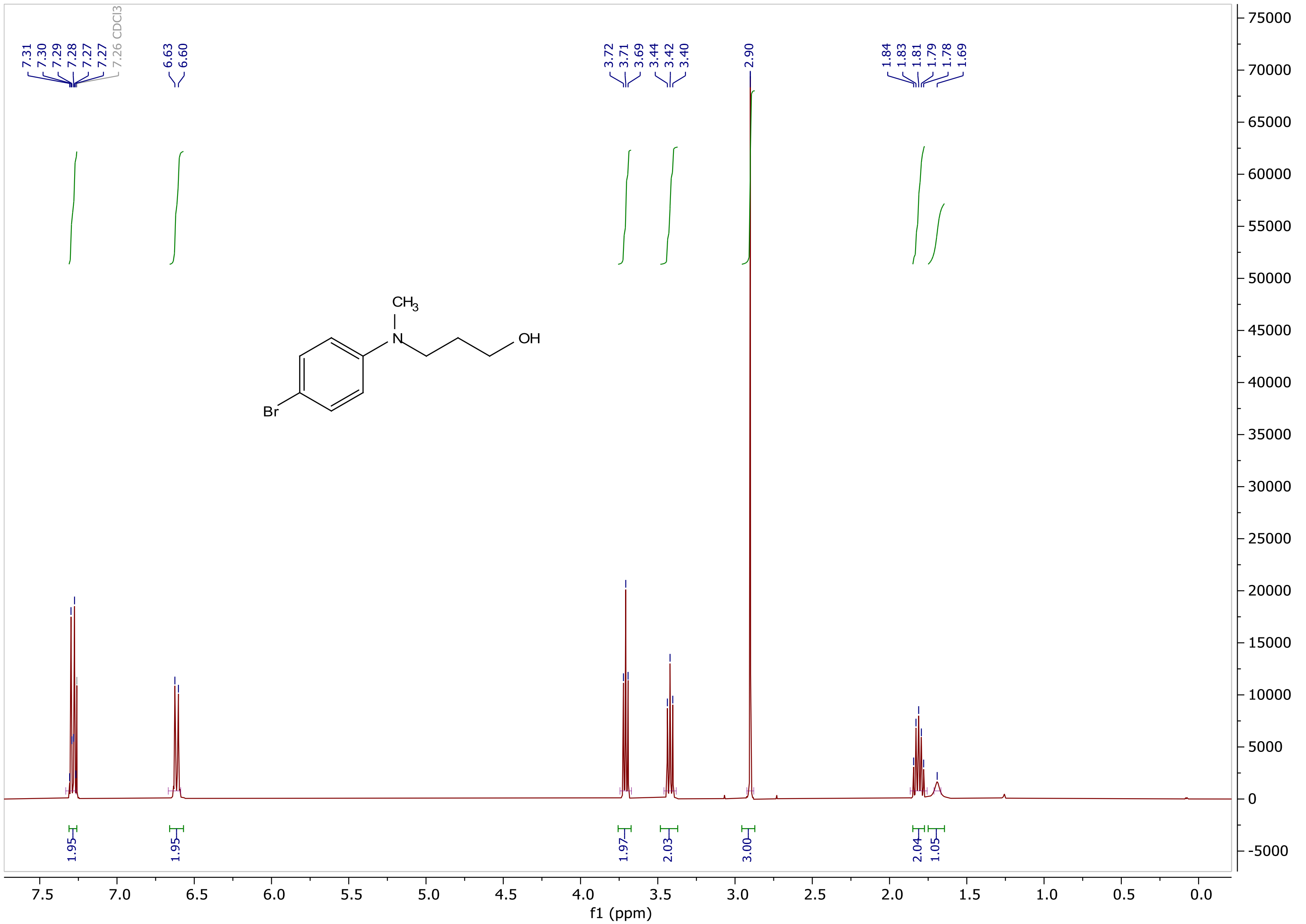

^{1}H NMR (400 MHz, $CDCl_3$) spectrum of compound **3j.**

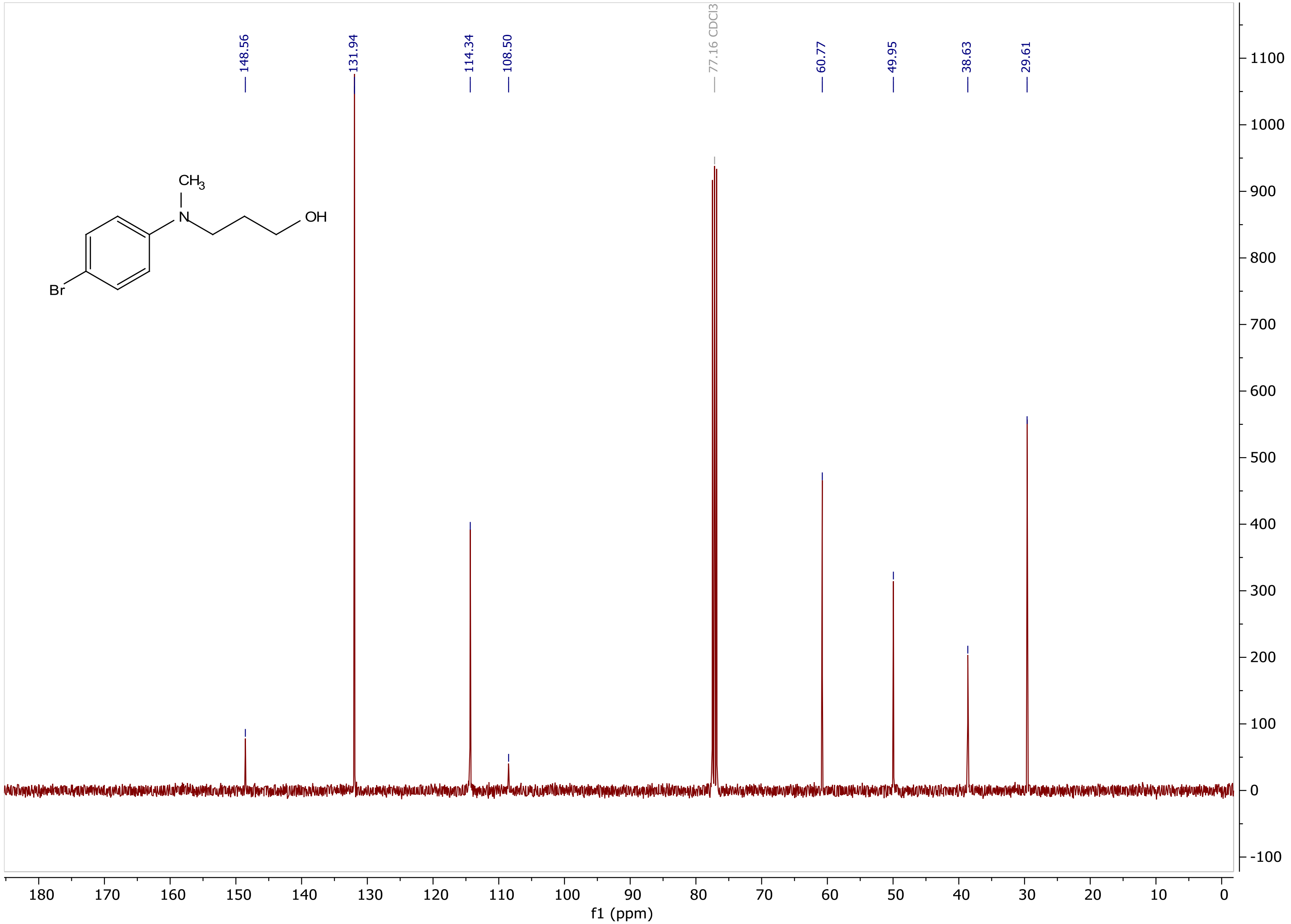

^{13}C NMR (101 MHz, $CDCl_3$) spectrum of compound **3j.**

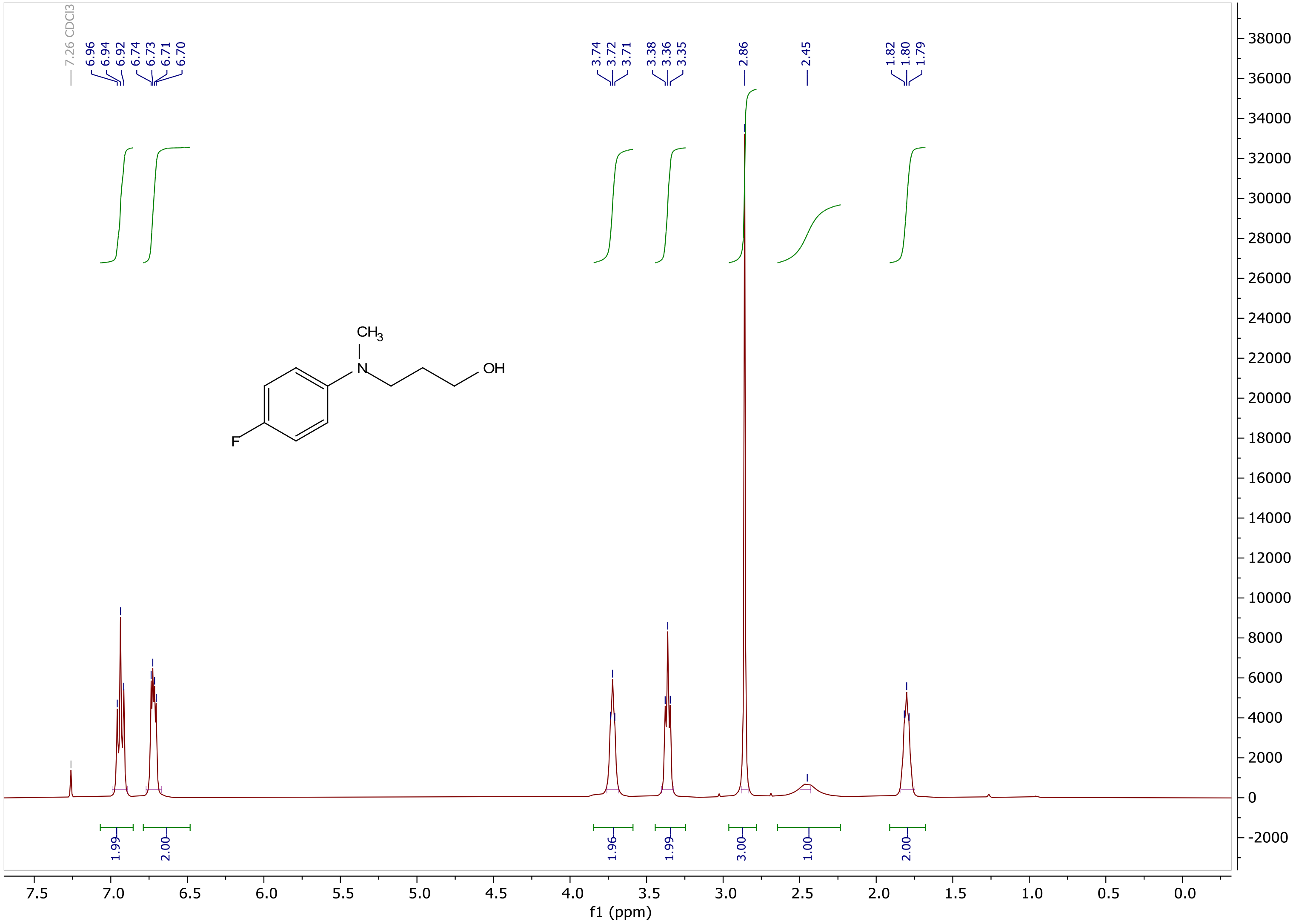

^1H NMR (400 MHz, $CDCl_3$) spectrum of compound **3k.**

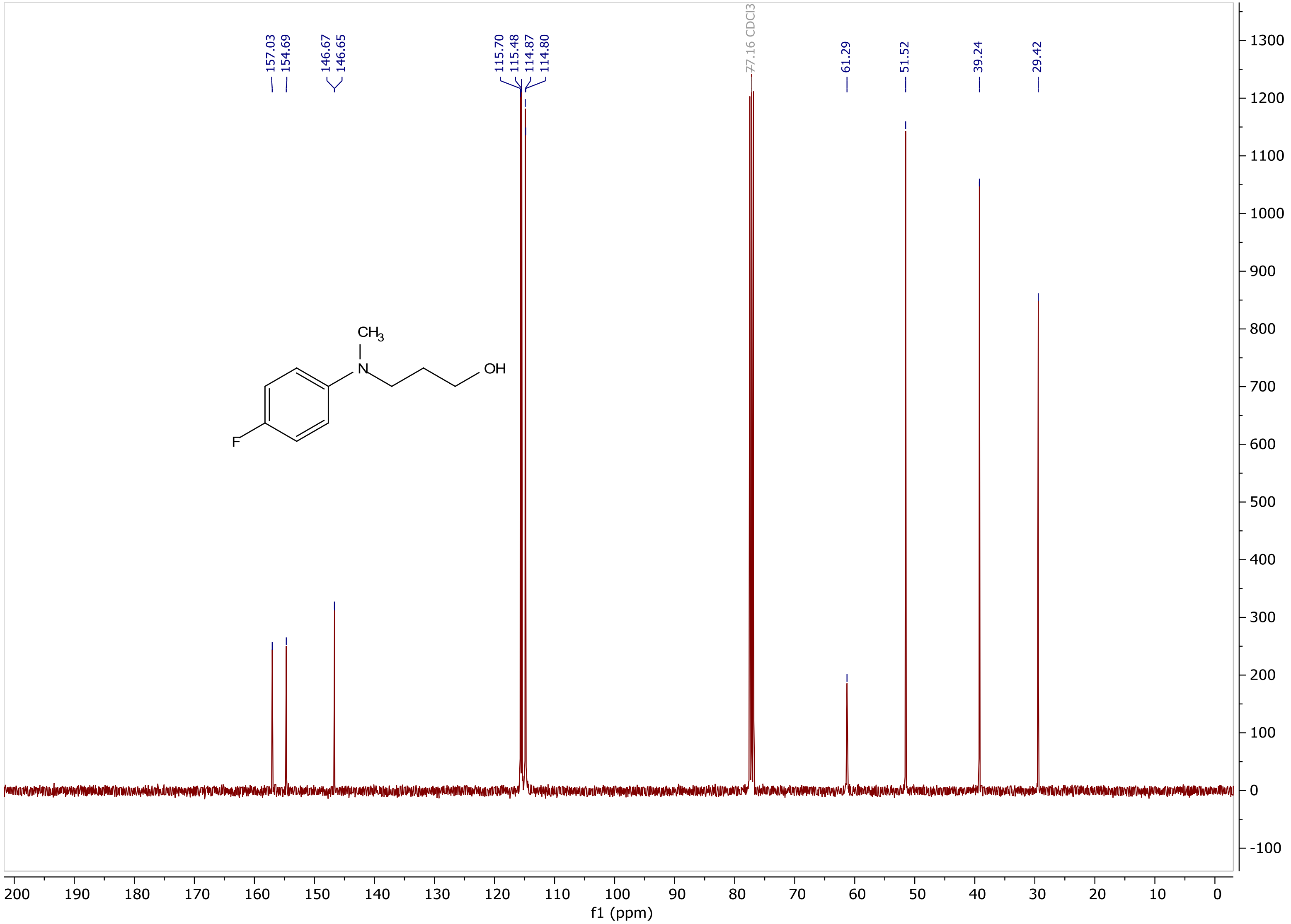

^{13}C NMR (101 MHz, $CDCl_3$) spectrum of compound **3k.**

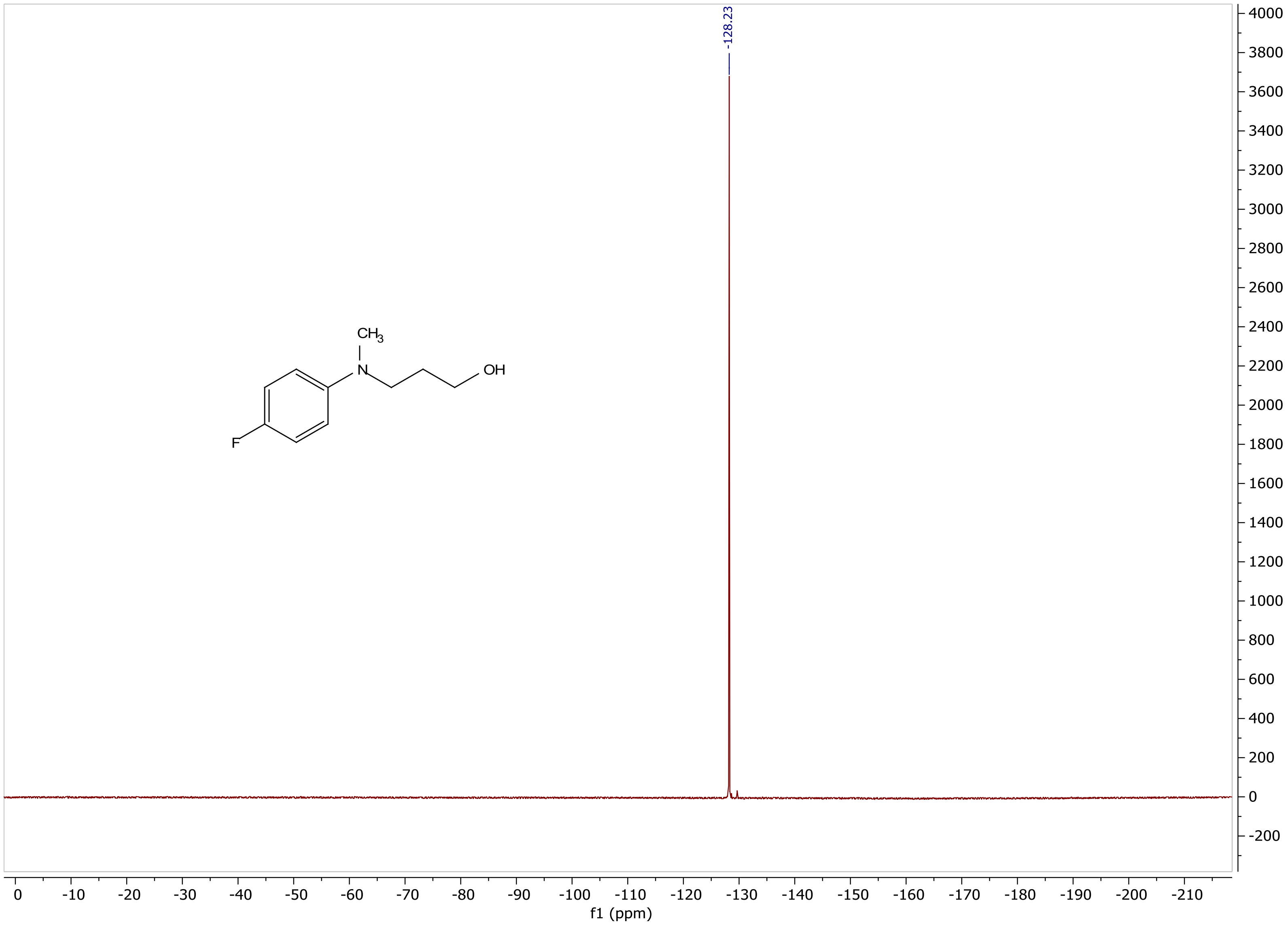

^{19}F NMR (376 MHz, $CDCl_3$) spectrum of compound **3k.**

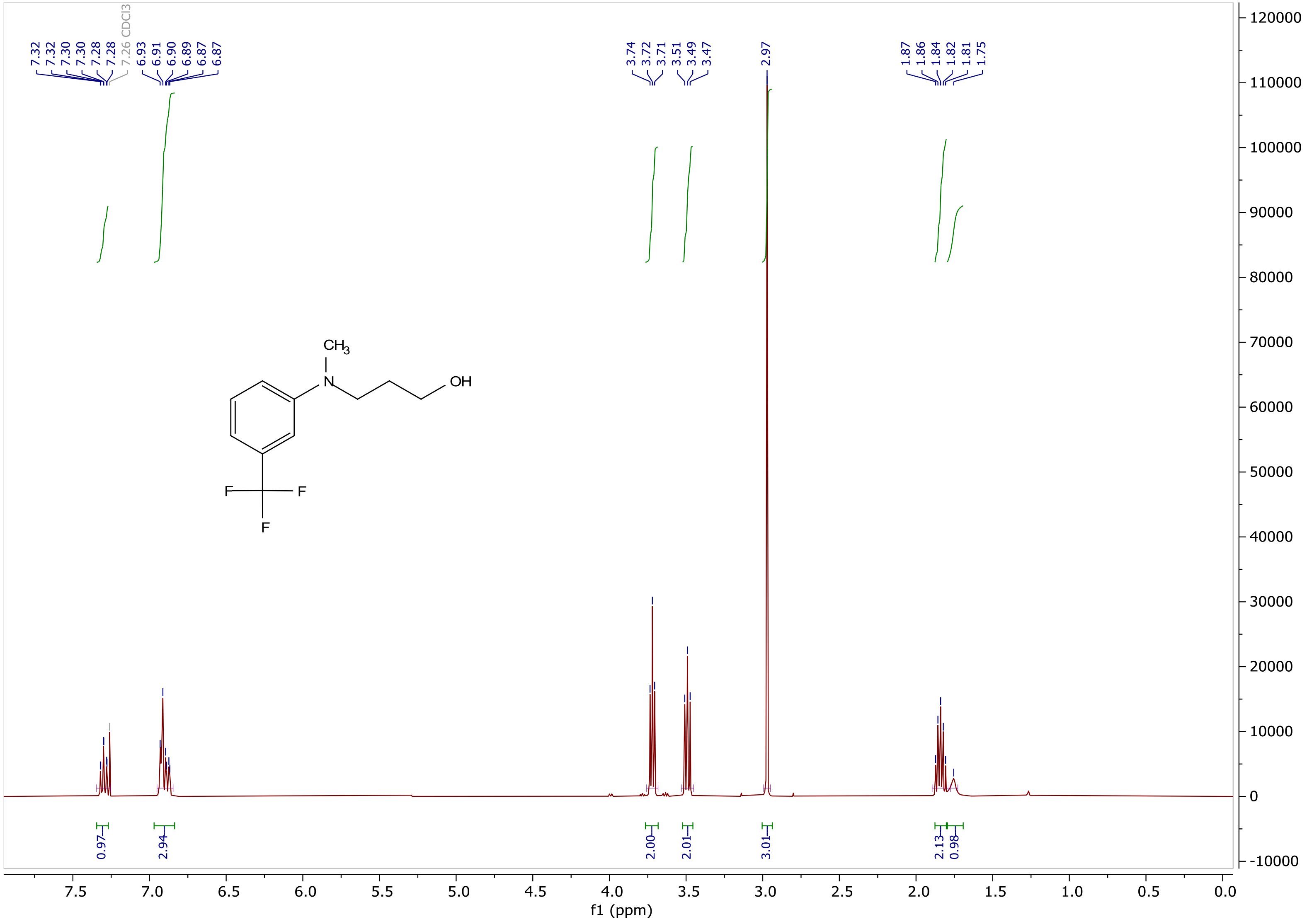

^{1}H NMR (400 MHz, $CDCl_3$) spectrum of compound **3l.**

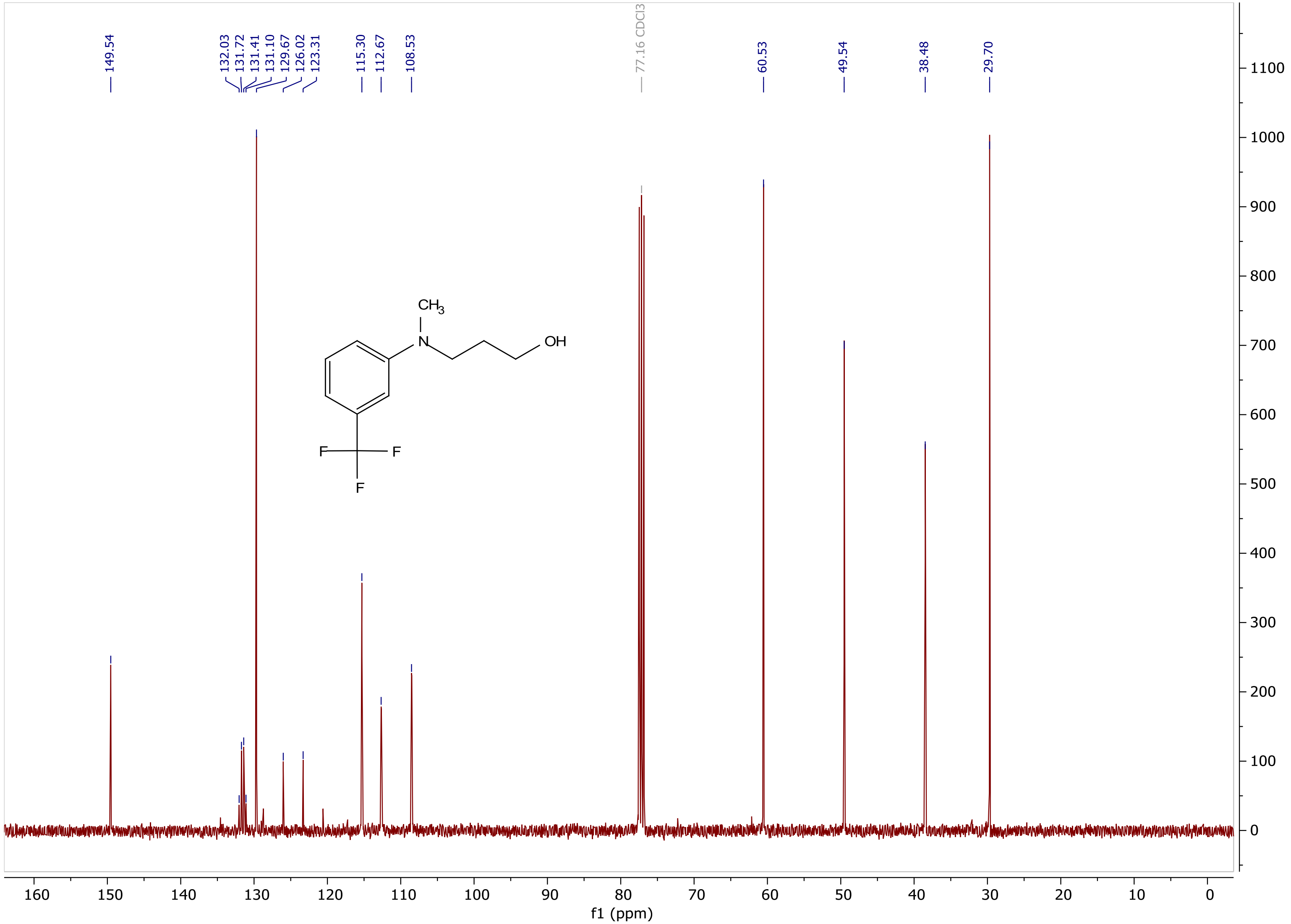

^{13}C NMR (101 MHz, $CDCl_3$) spectrum of compound **3l.**

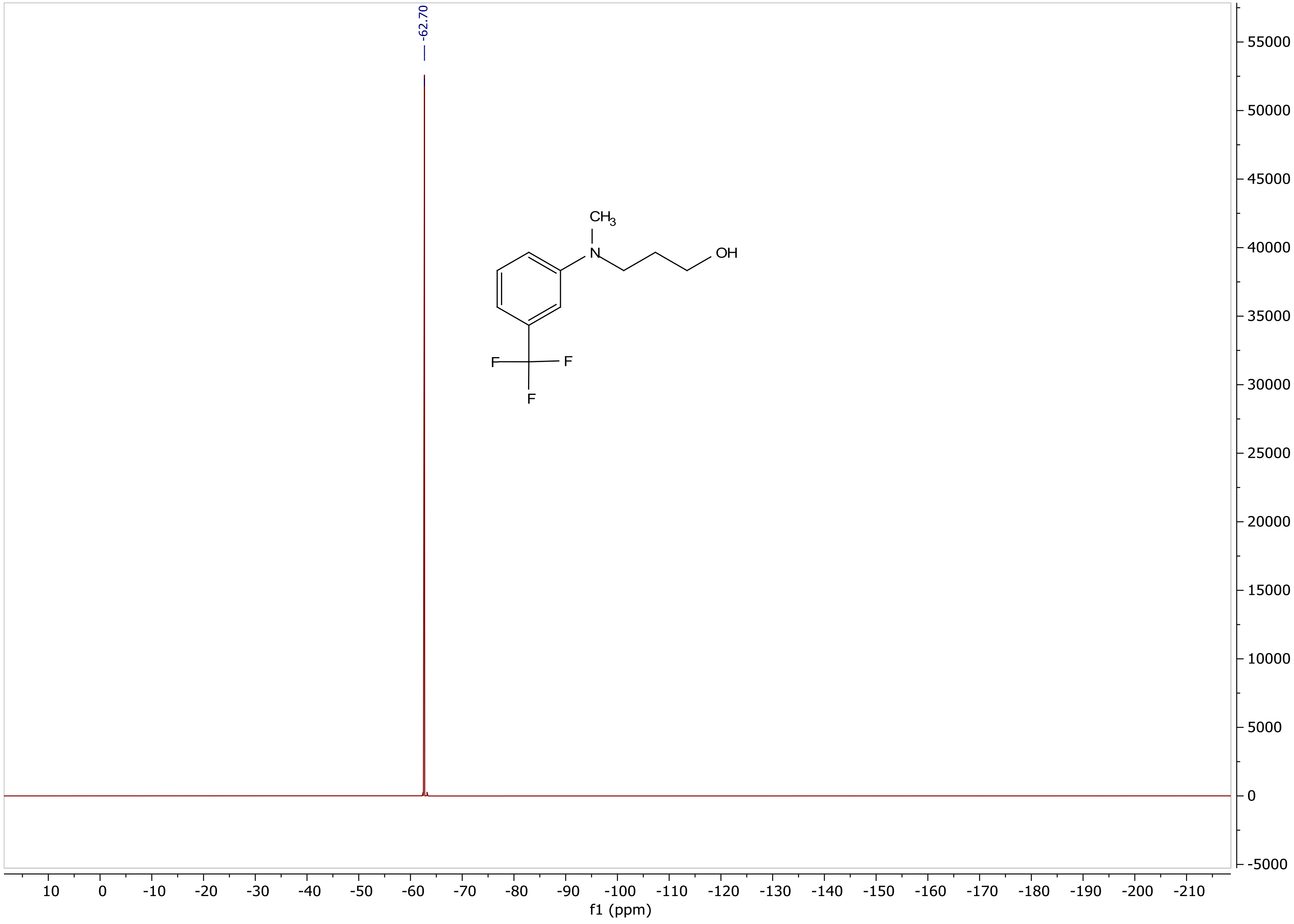

^{19}F NMR (376 MHz, $CDCl_3$) spectrum of compound **3l.**

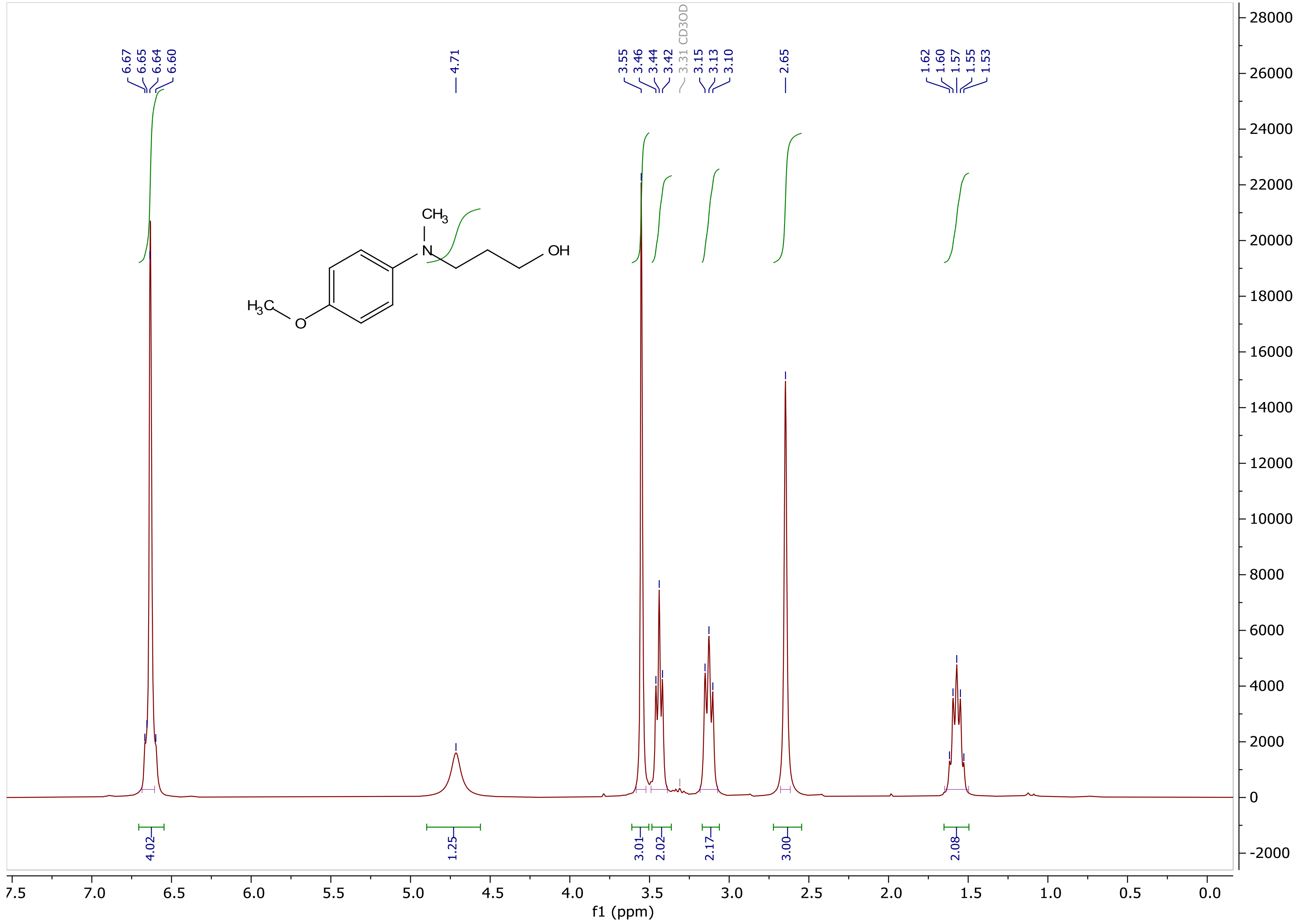

^{1}H NMR (300 MHz, CD_3OD) spectrum of compound **3m.**

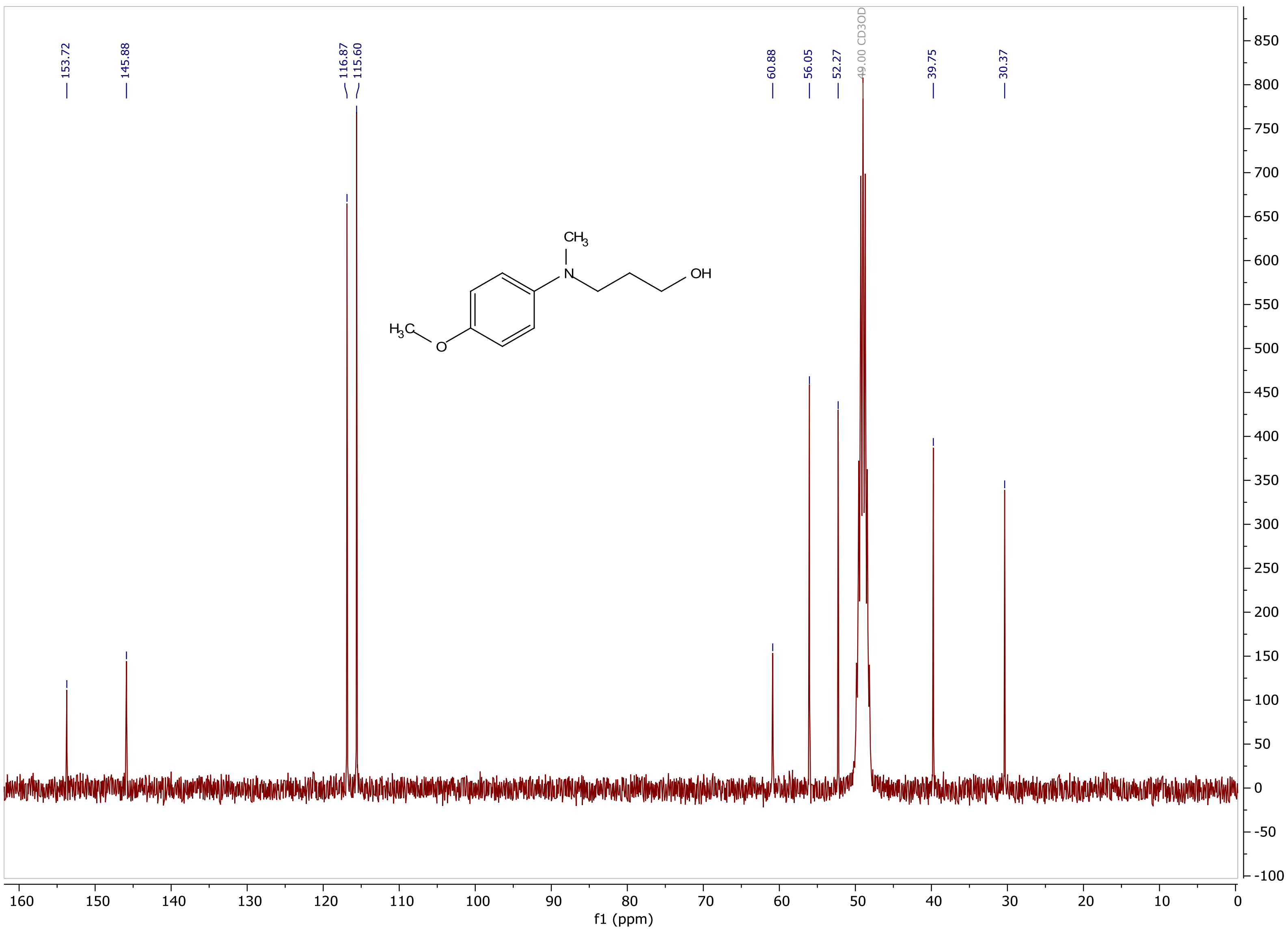

^{13}C NMR (75 MHz, CD_3OD) spectrum of compound **3m.**

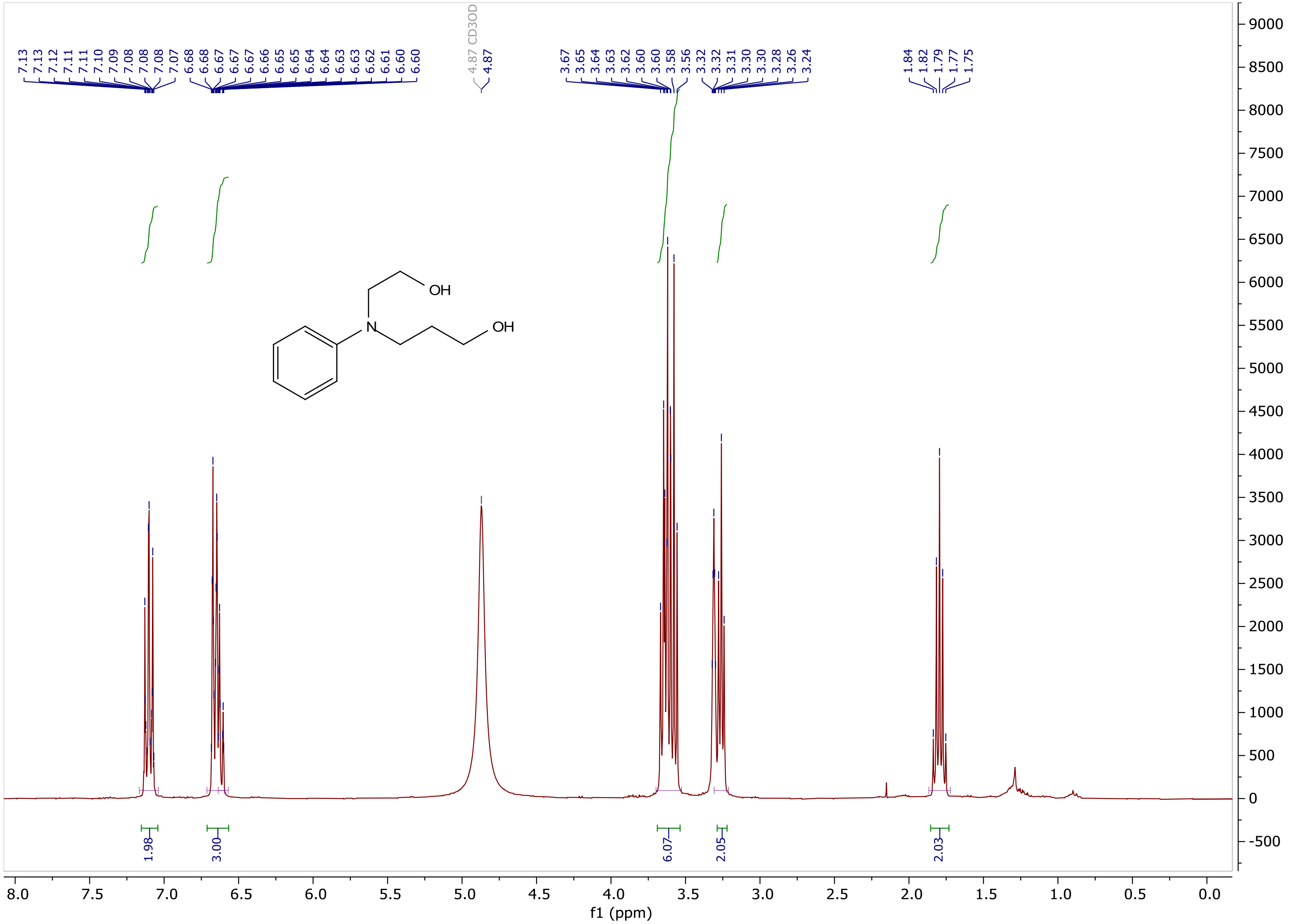

^{1}H NMR (300 MHz, CD_3OD) spectrum of compound **3n.**

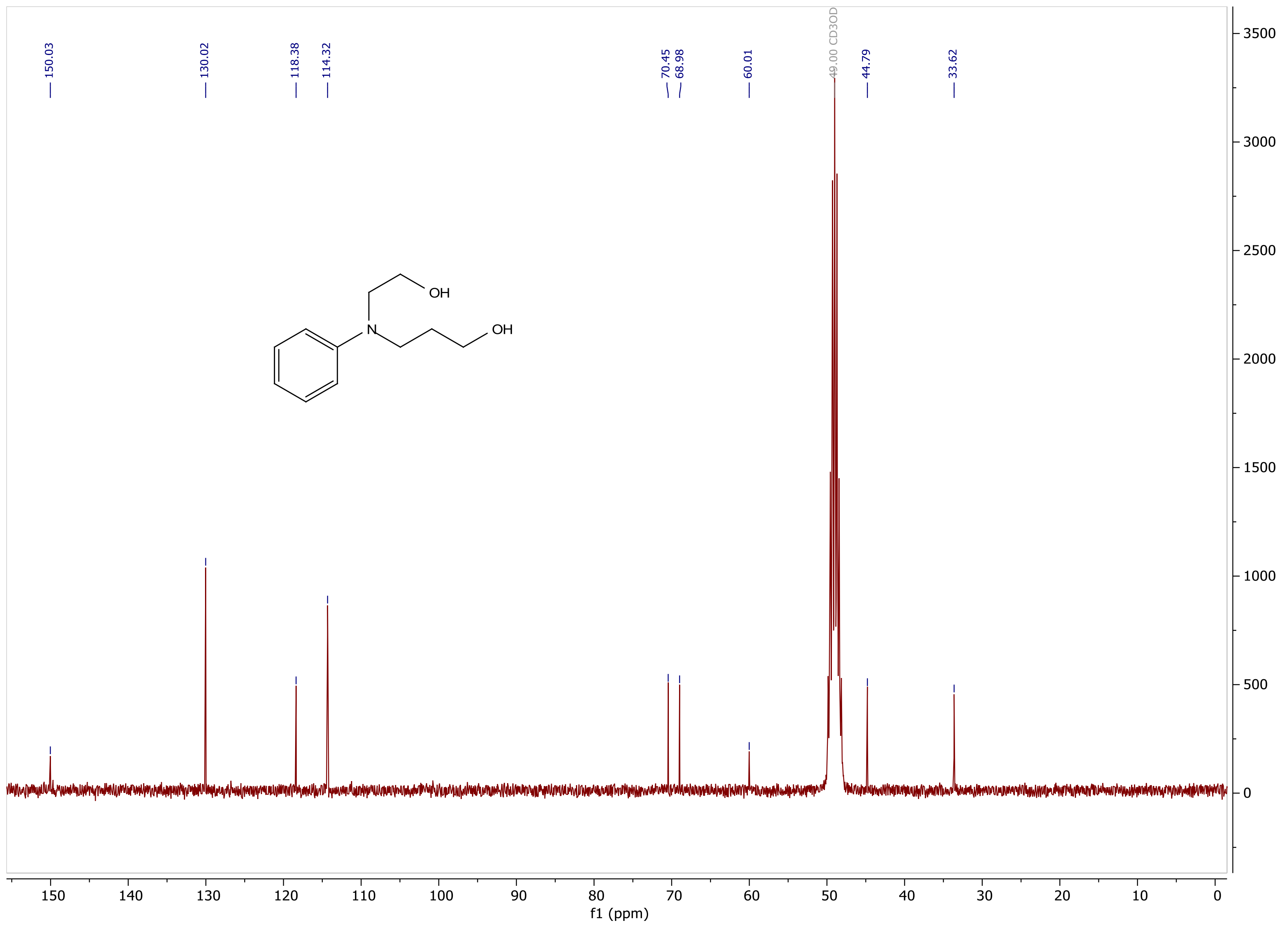

^{13}C NMR (75 MHz, CD_3OD) spectrum of compound **3n.**

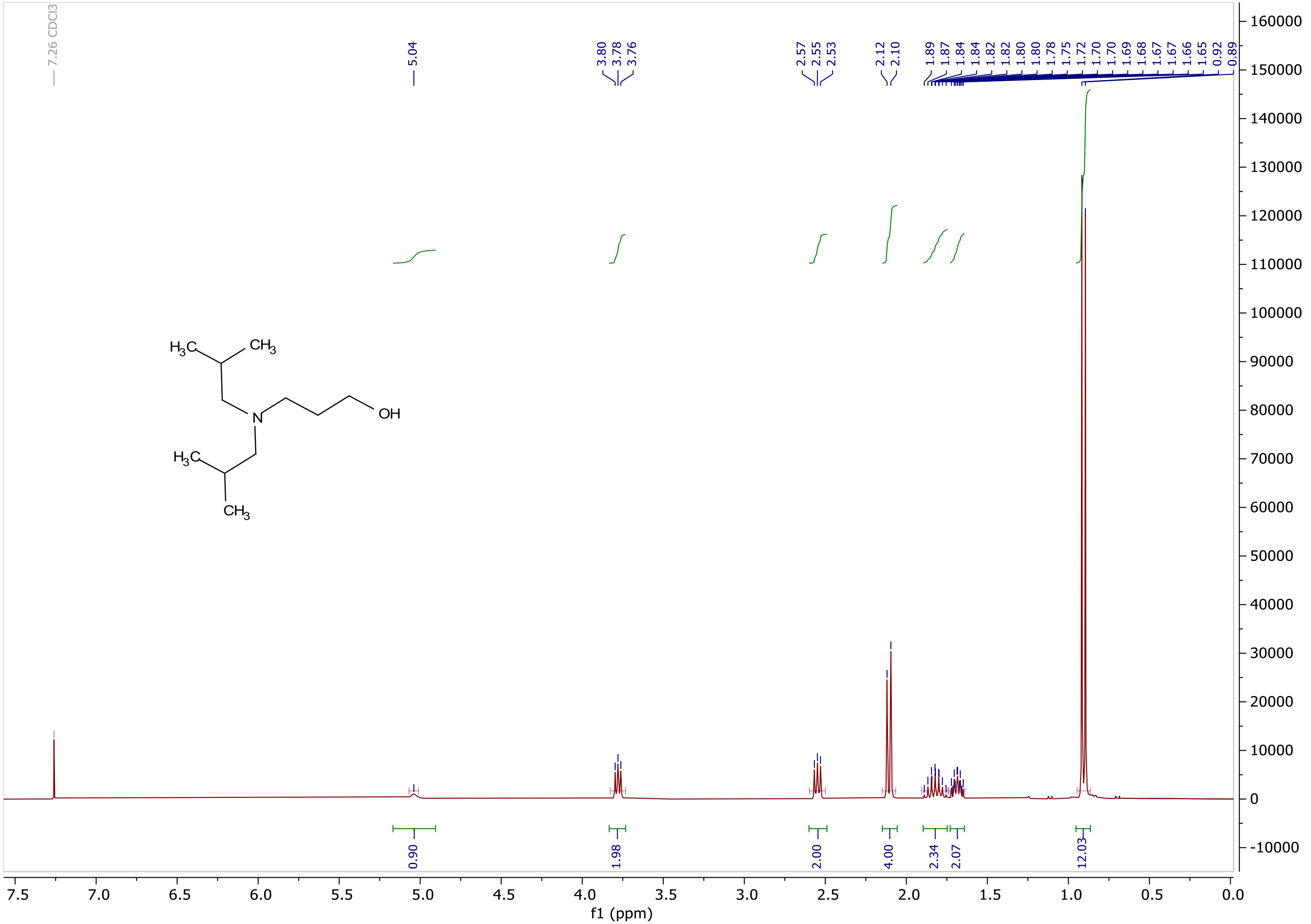

^{1}H NMR (300 MHz, $CDCl_3$) spectrum of compound **3o.**

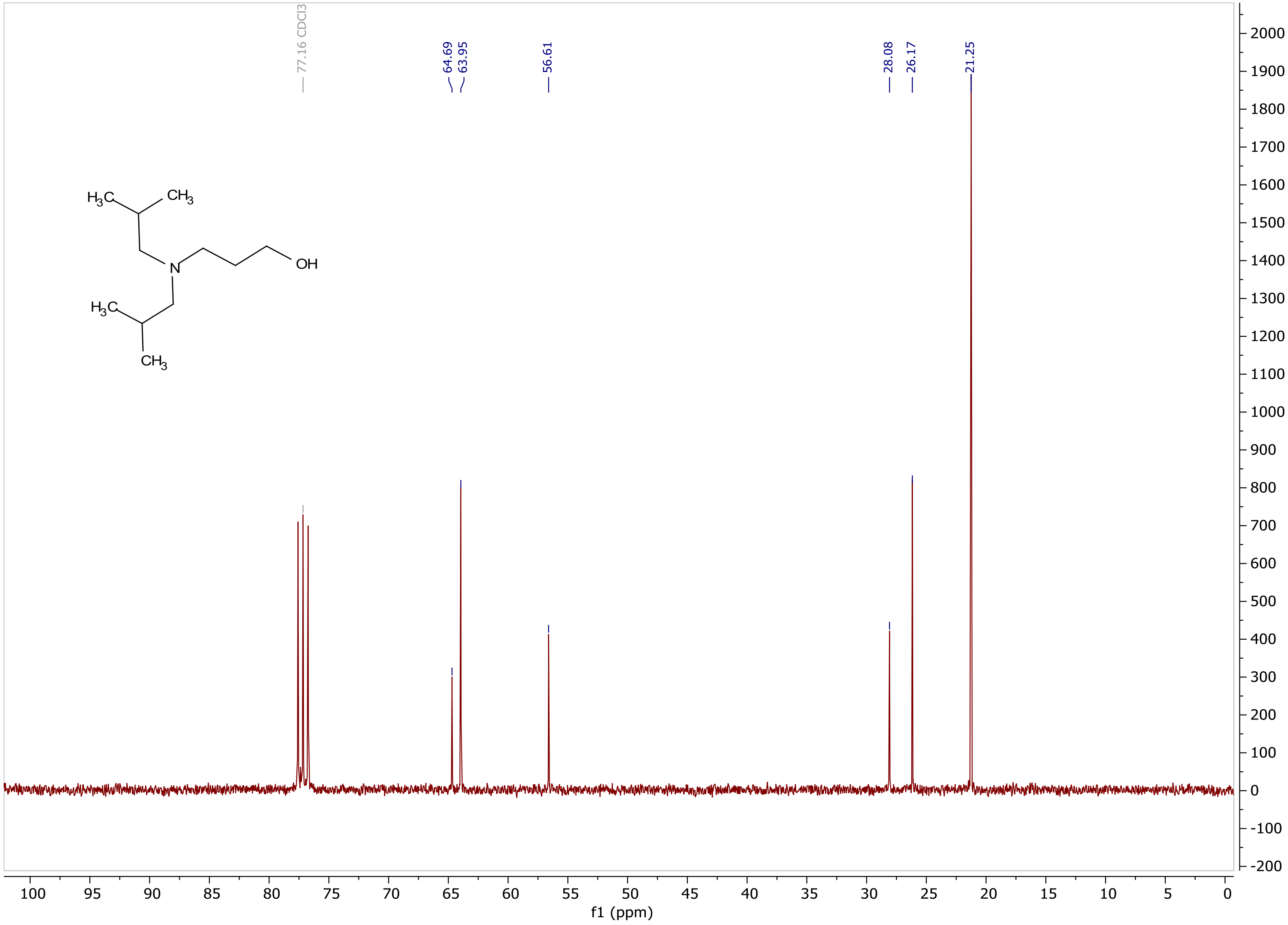

^{13}C NMR (75 MHz, $CDCl_3$) spectrum of compound **3o.**

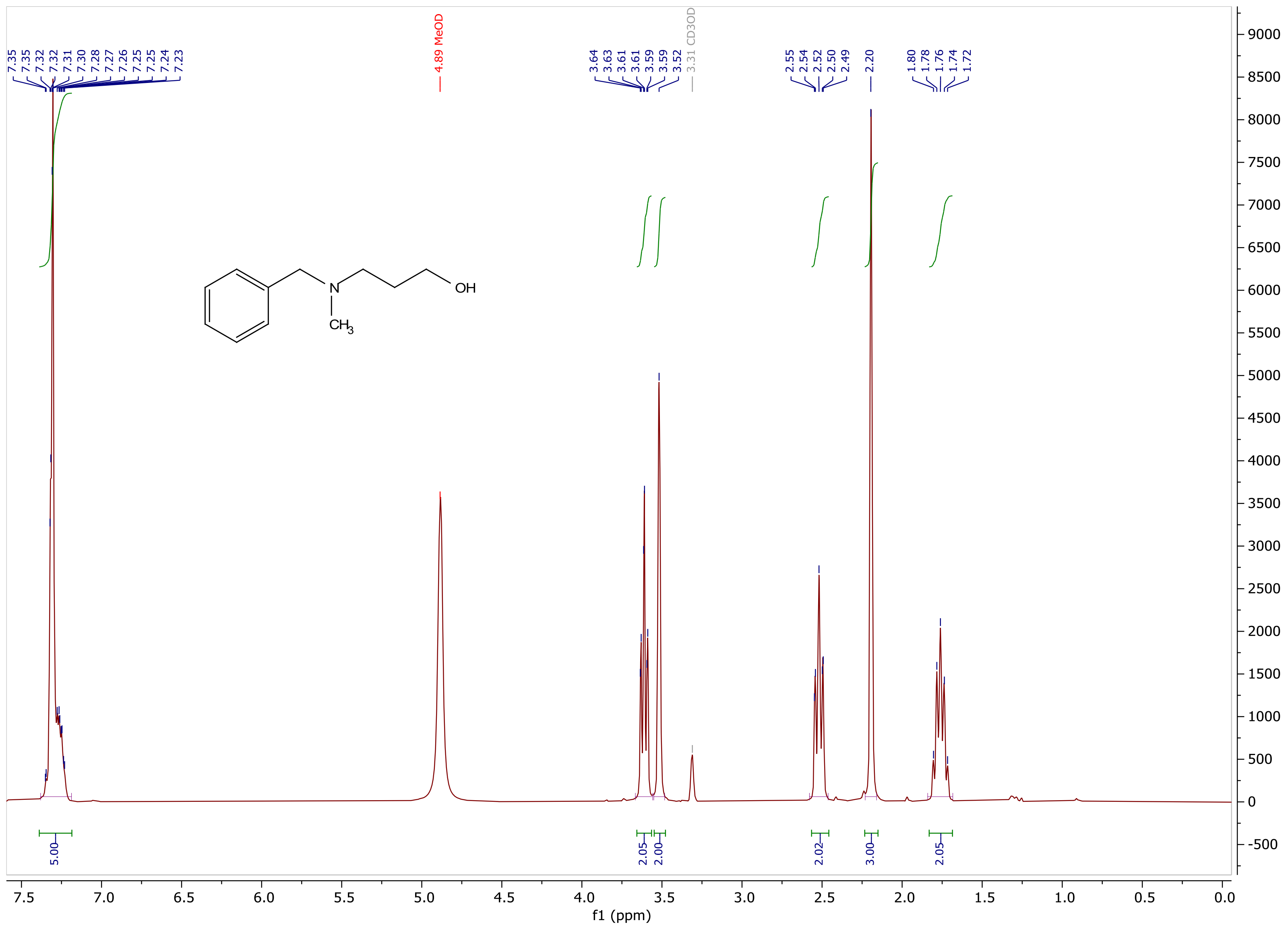

^{1}H NMR (300 MHz, CD_3OD) spectrum of compound **3p.**

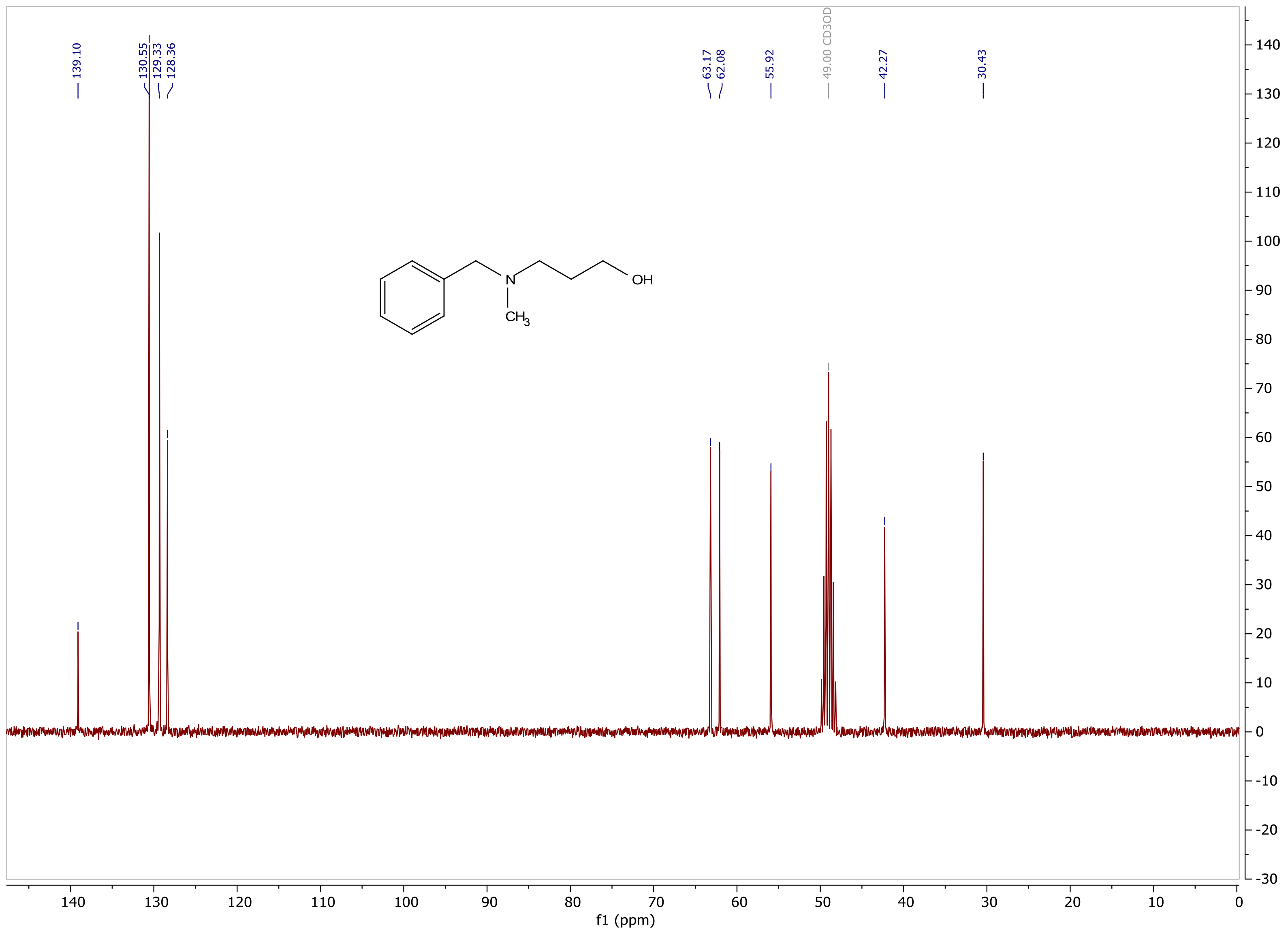

^{13}C NMR (75 MHz, CD_3OD) spectrum of compound **3p.**

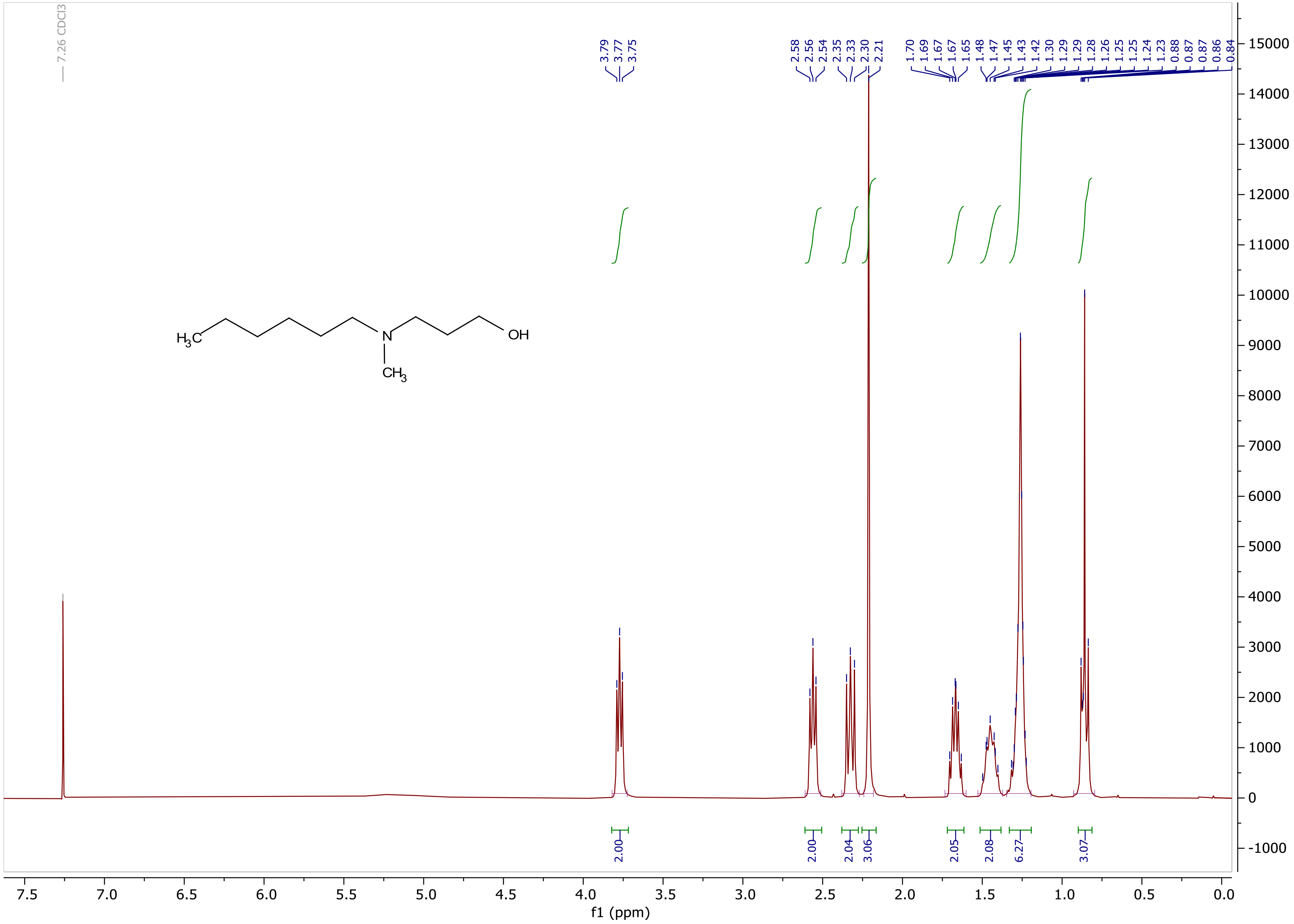

^1H NMR (300 MHz, $CDCl_3$) spectrum of compound **3q.**

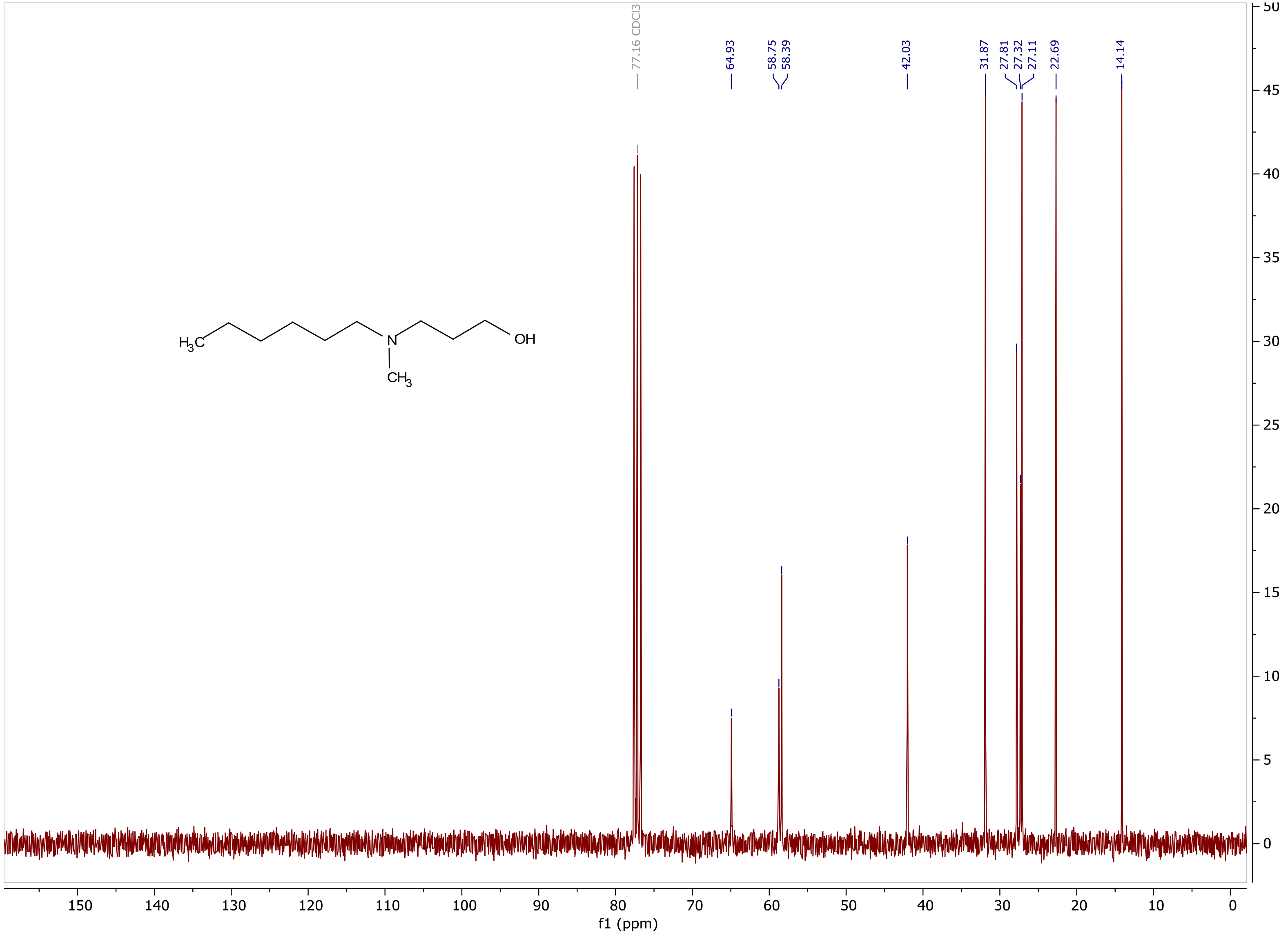

^{13}C NMR (75 MHz, $CDCl_3$) spectrum of compound **3q.**

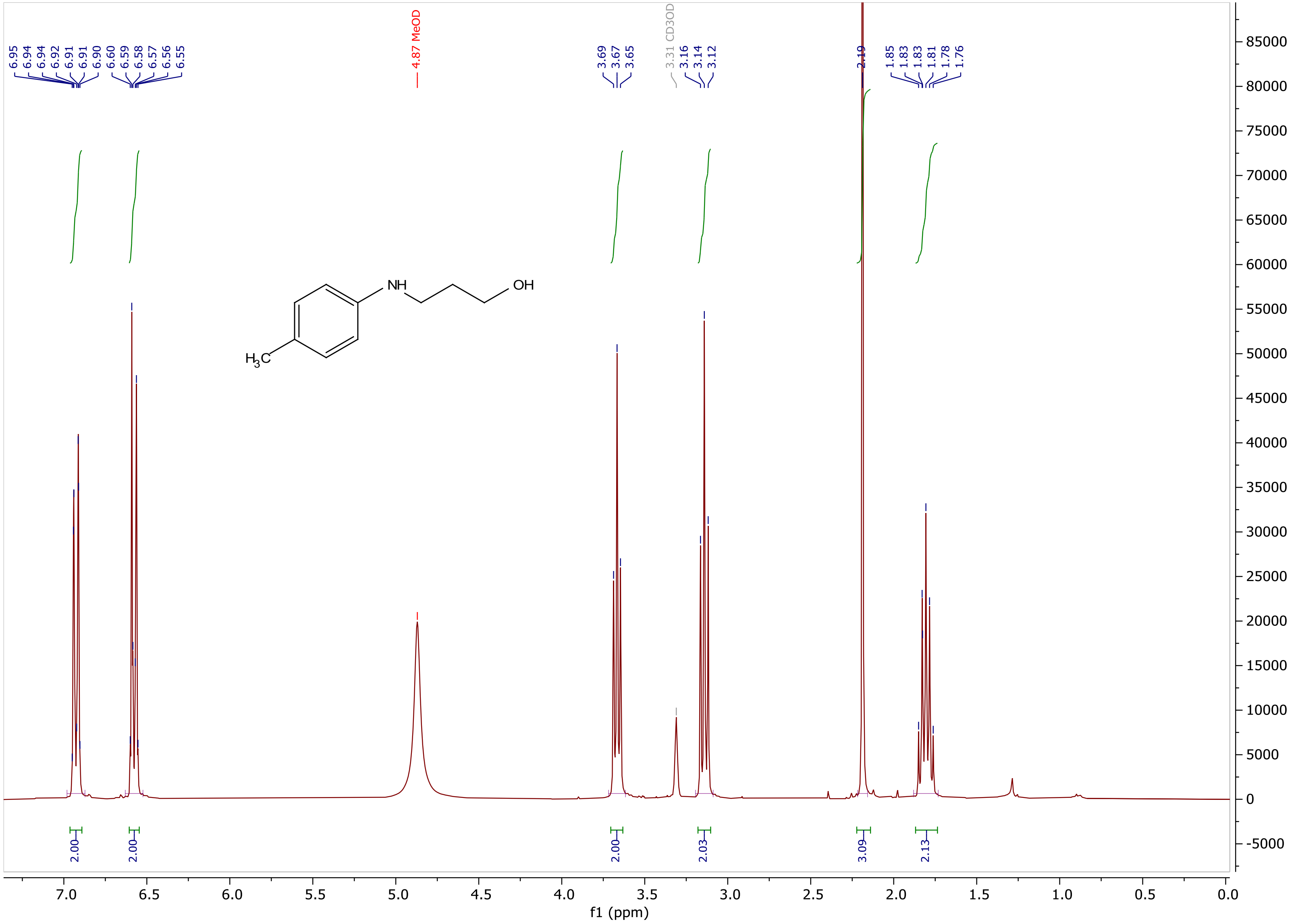

^{1}H NMR (300 MHz, CD_3OD) spectrum of compound **3r.**

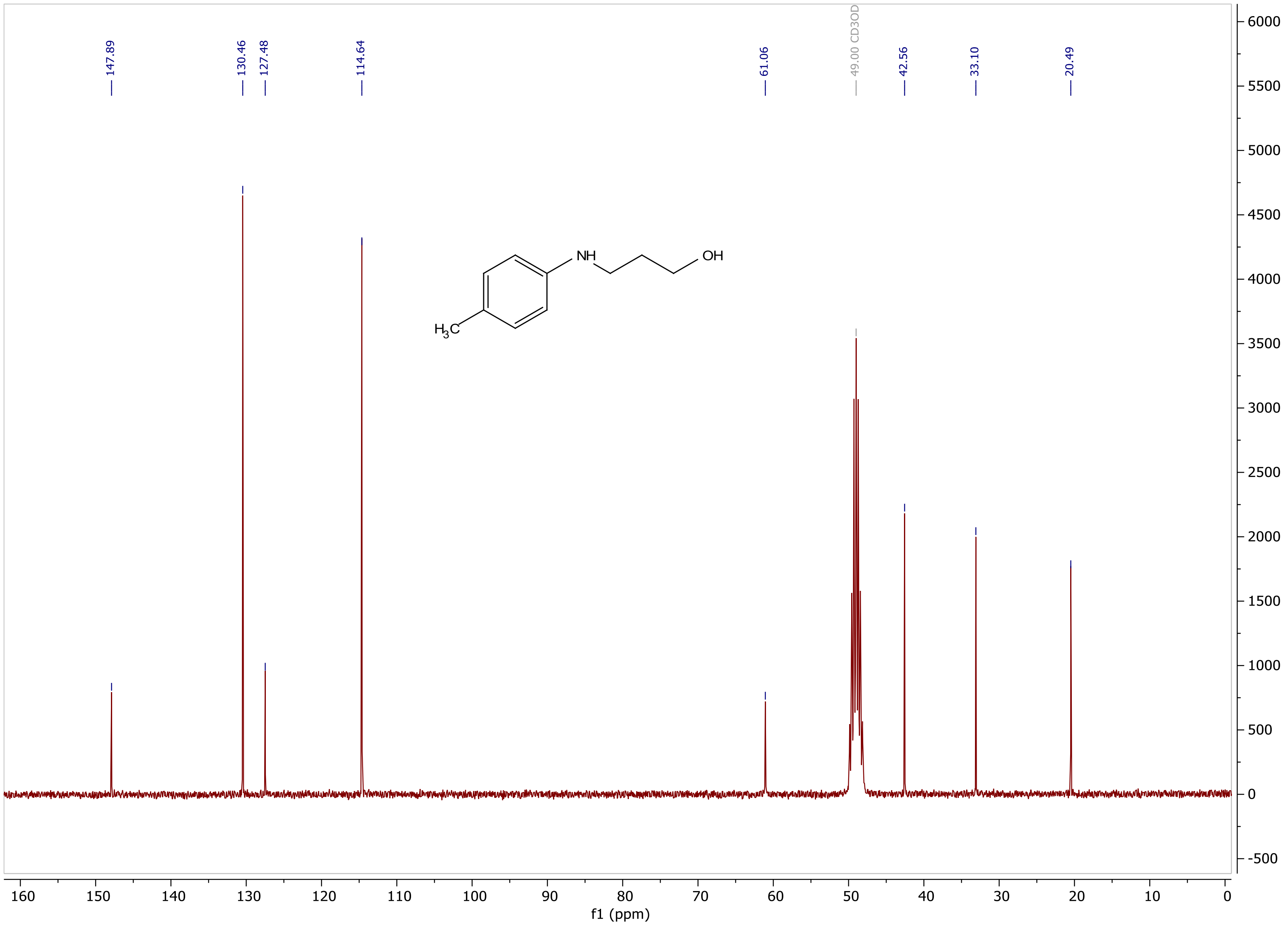

^{13}C NMR (75 MHz, CD_3OD) spectrum of compound **3r.**

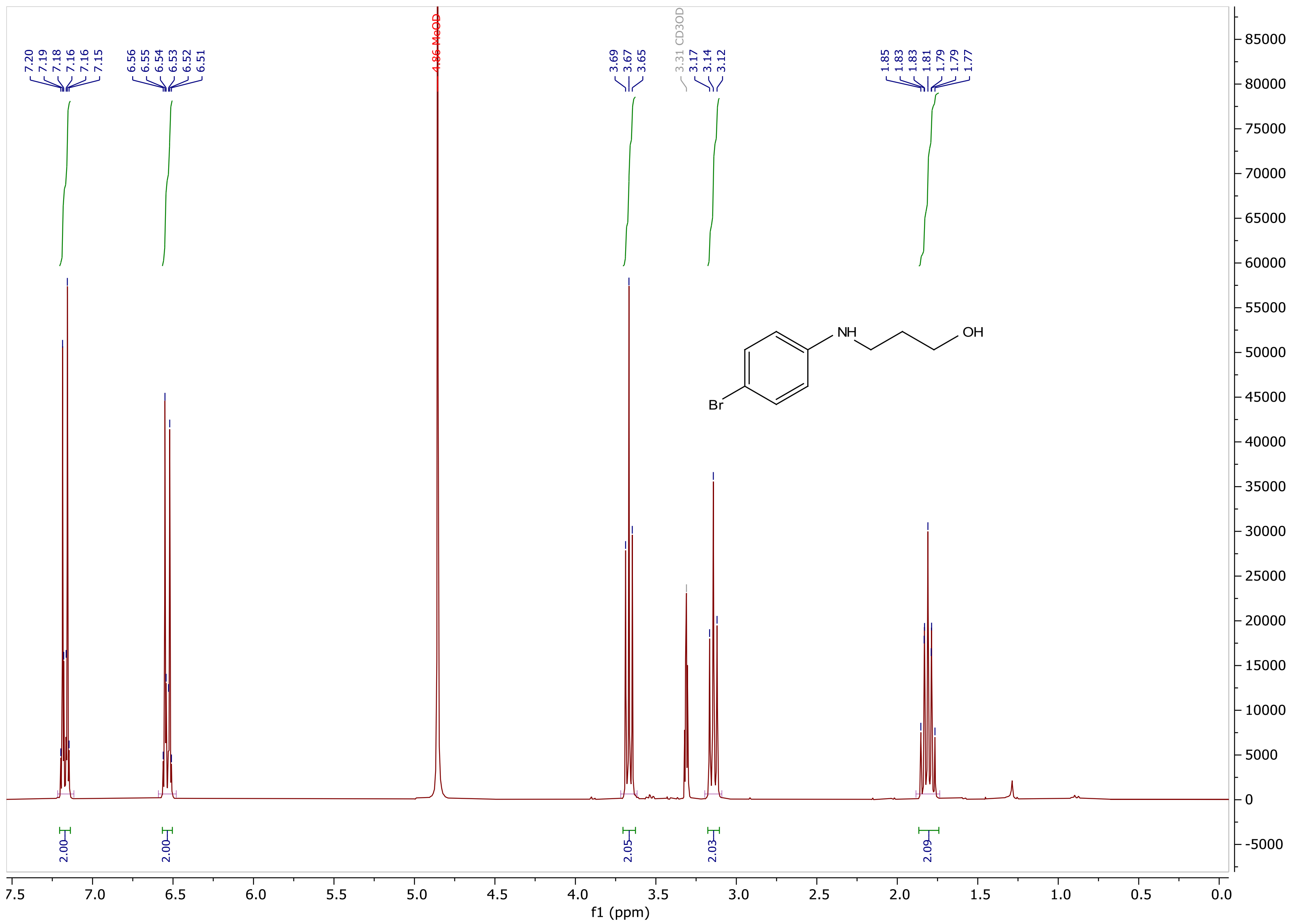

^{1}H NMR (300 MHz, CD_3OD) spectrum of compound **3s.**

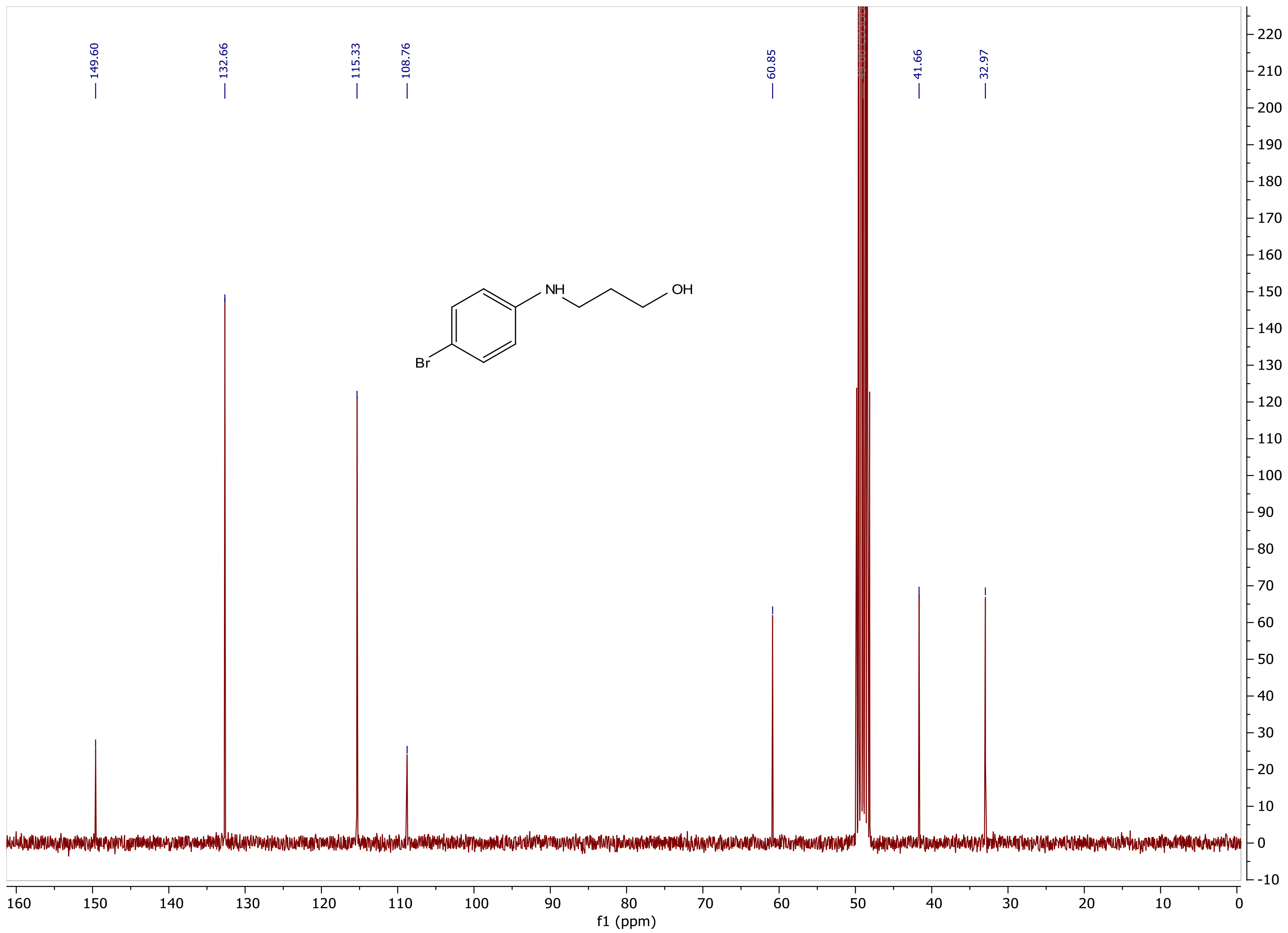

^{13}C NMR (75 MHz, CD_3OD) spectrum of compound **3s.**

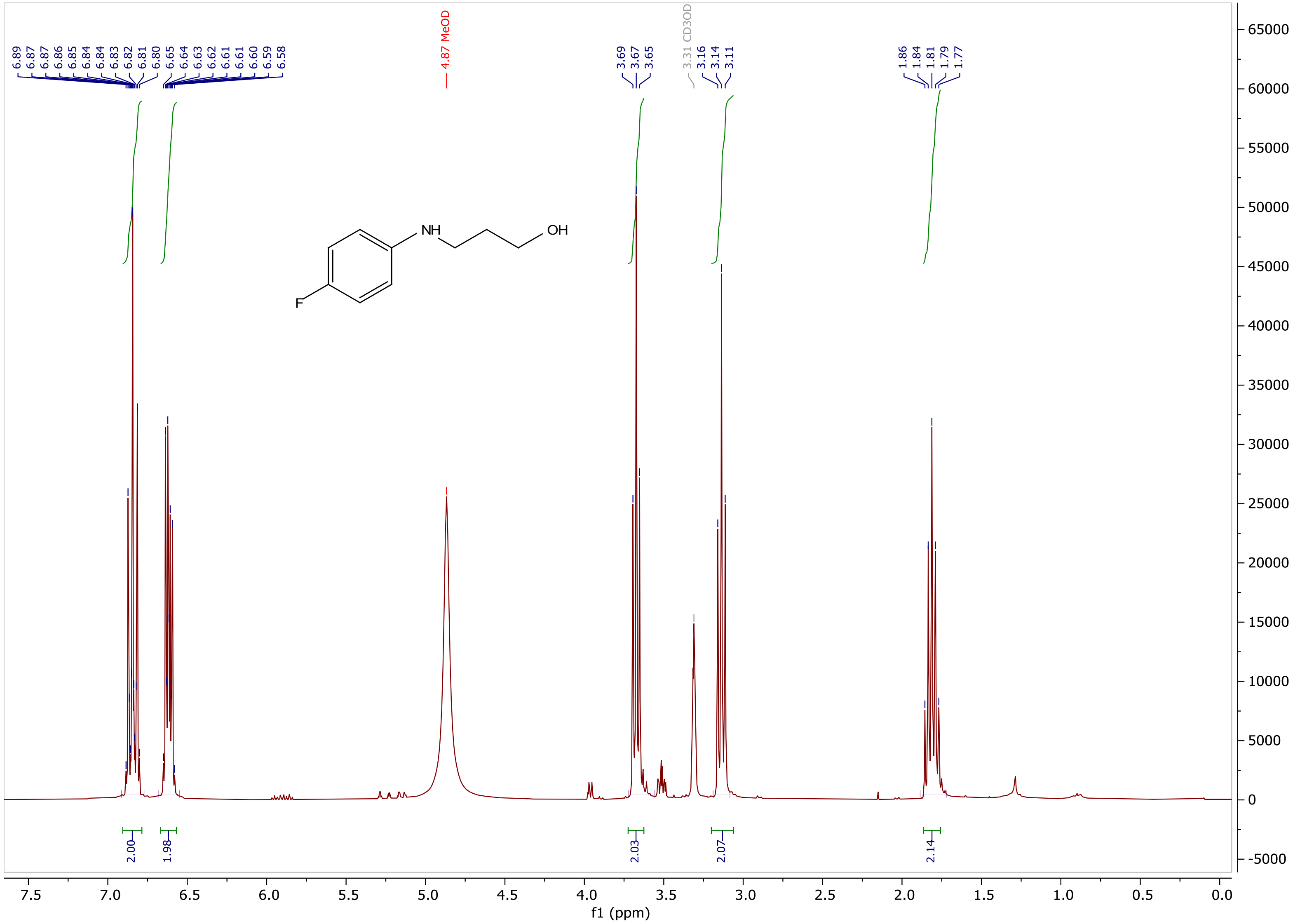

^{1}H NMR (300 MHz, CD_3OD) spectrum of compound **3t.**

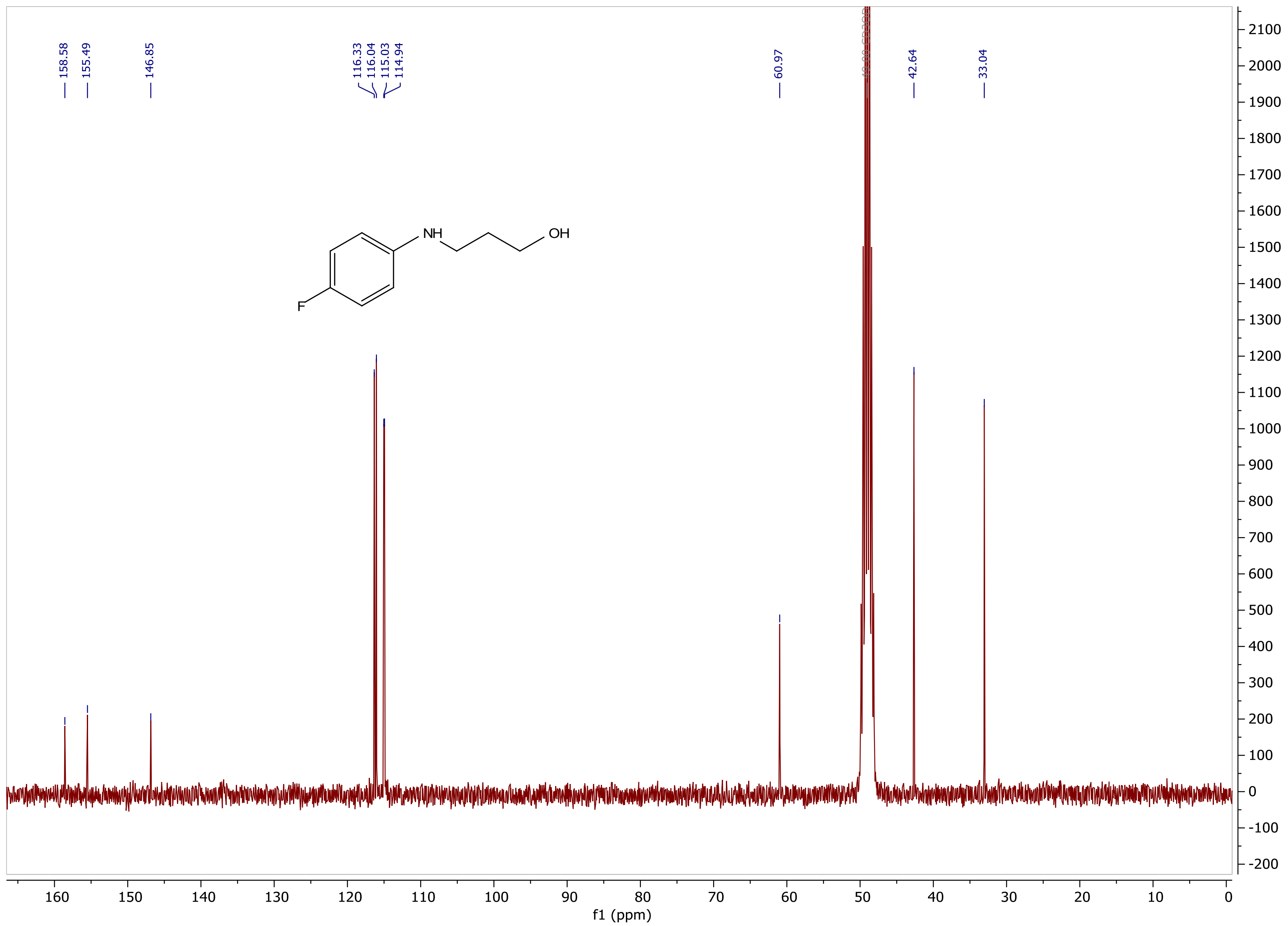

^{13}C NMR (75 MHz, CD_3OD) spectrum of compound **3t.**

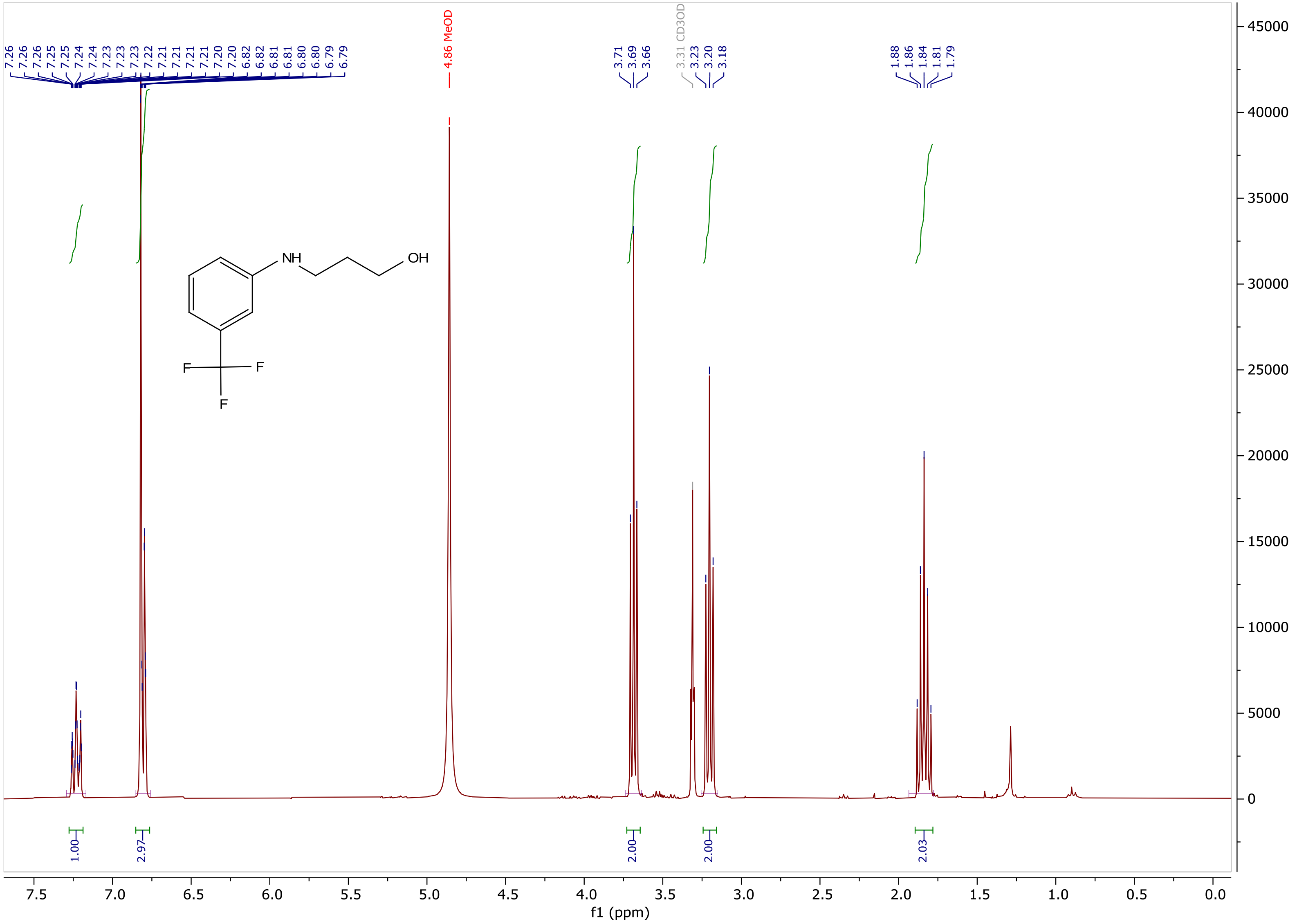

^{1}H NMR (300 MHz, CD_3OD) spectrum of compound **3u.**

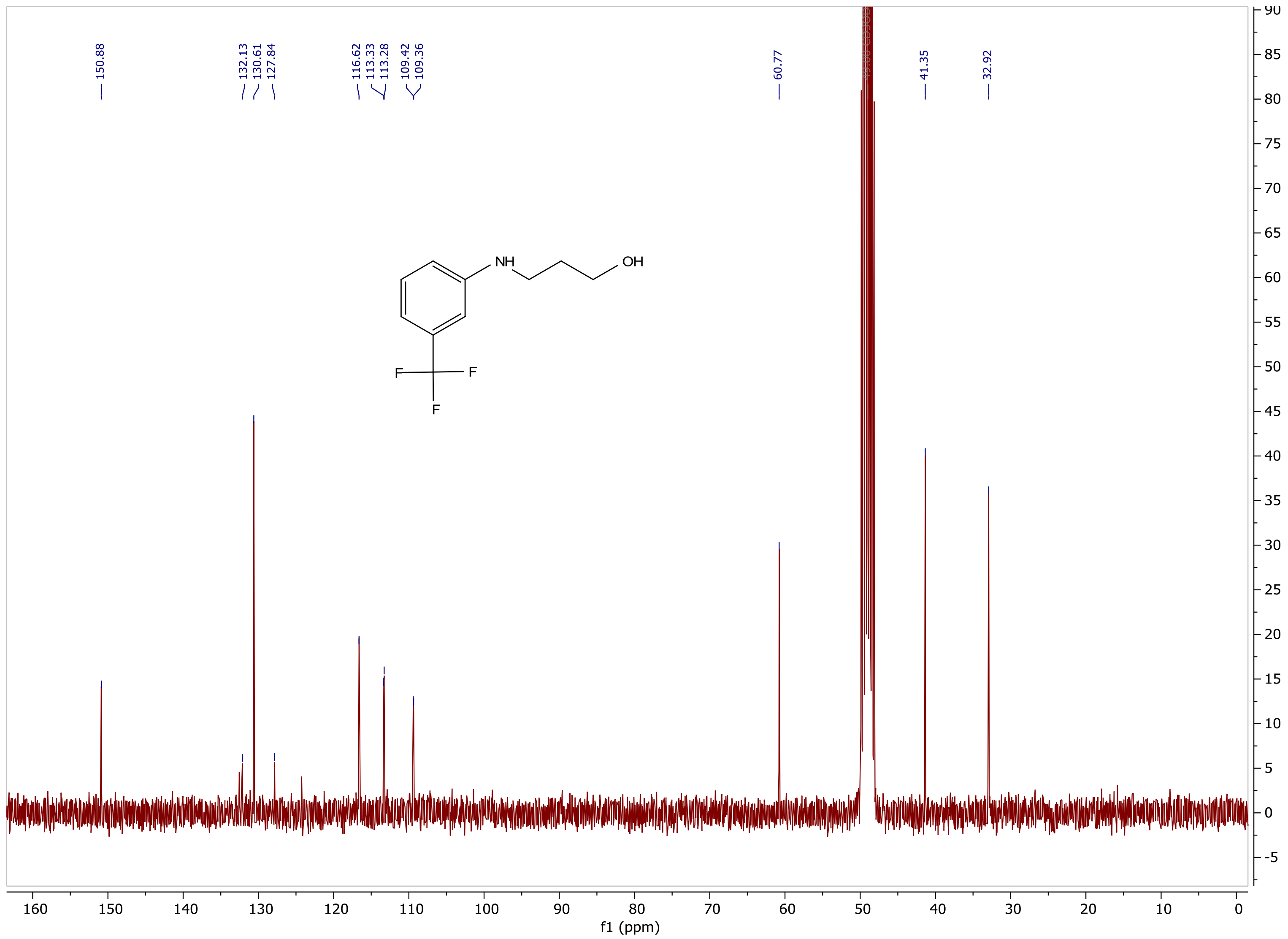

^{13}C NMR (75 MHz, CD_3OD) spectrum of compound **3u.**

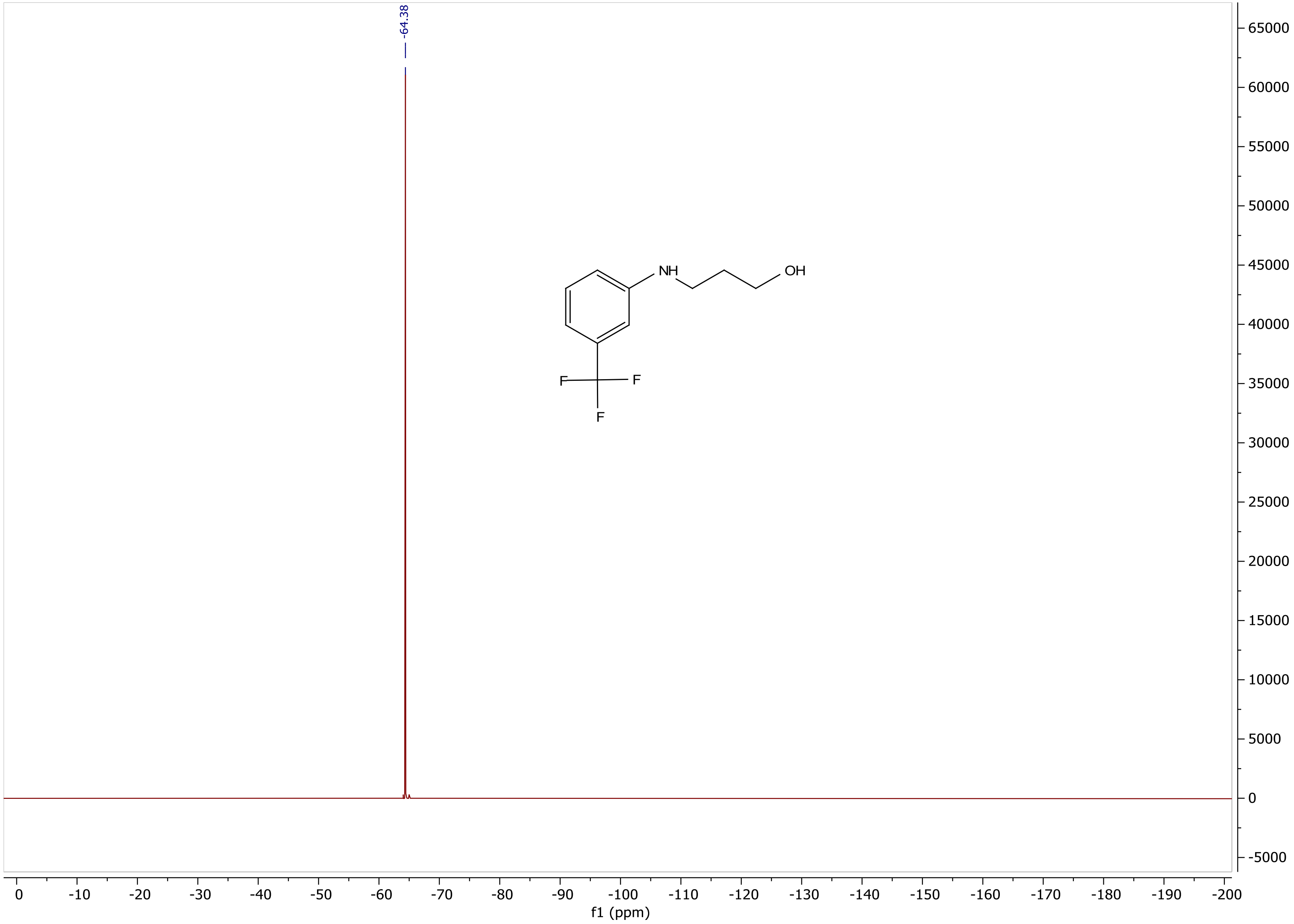

^{19}F NMR (282 MHz, CD_3OD) spectrum of compound **3u.**

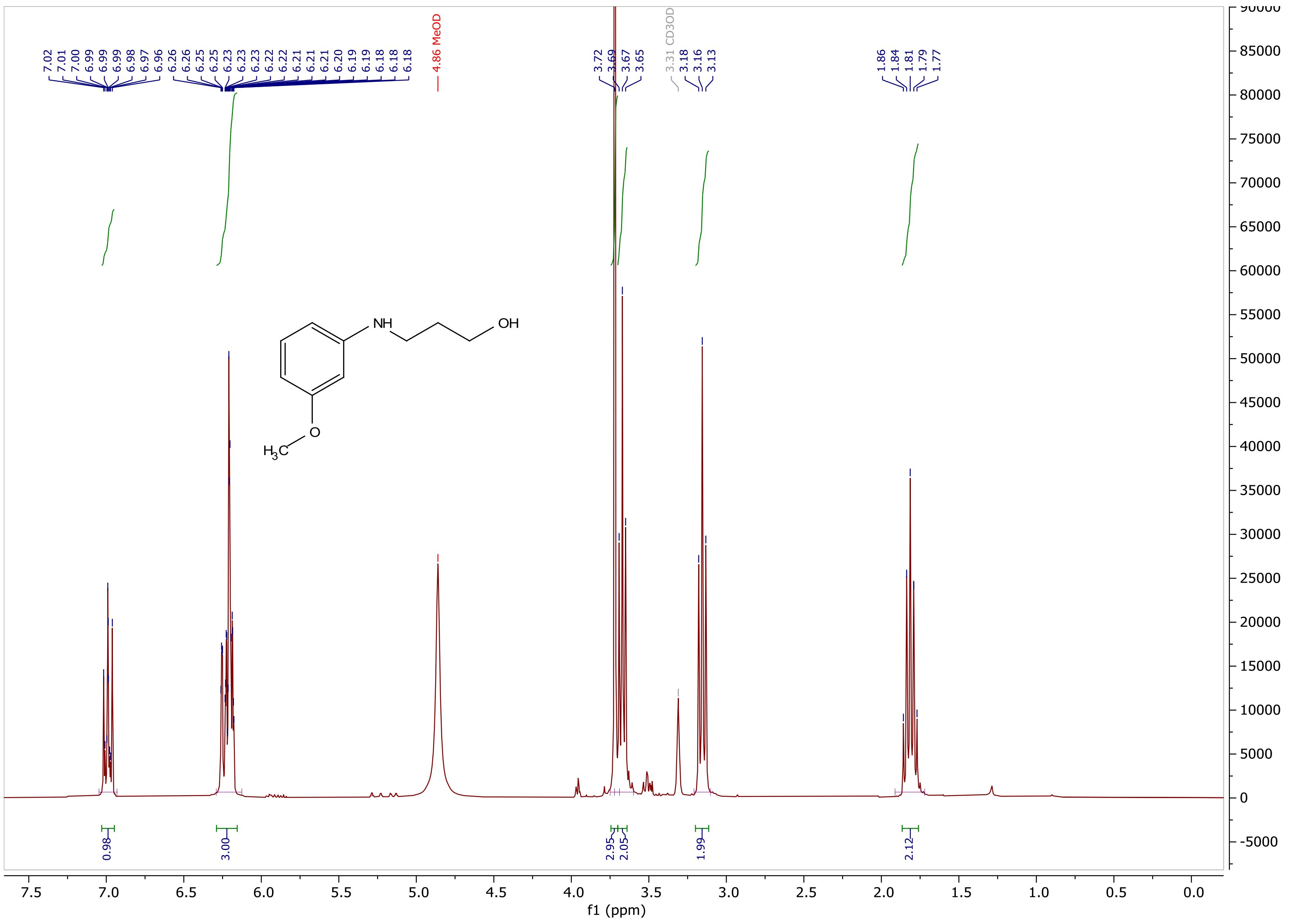

^{1}H NMR (300 MHz, CD_3OD) spectrum of compound **3v.**

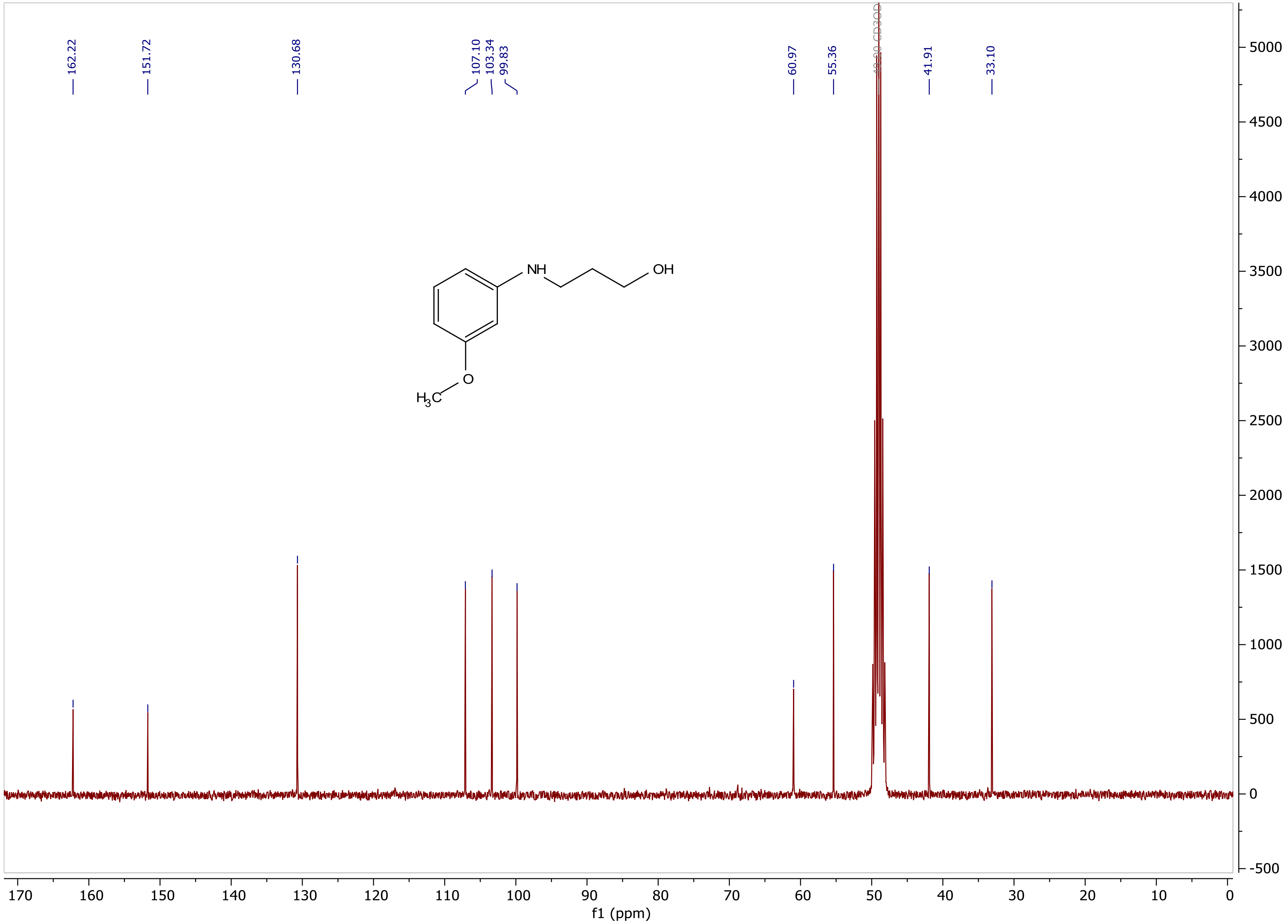

^{13}C NMR (75 MHz, CD_3OD) spectrum of compound **3v.**

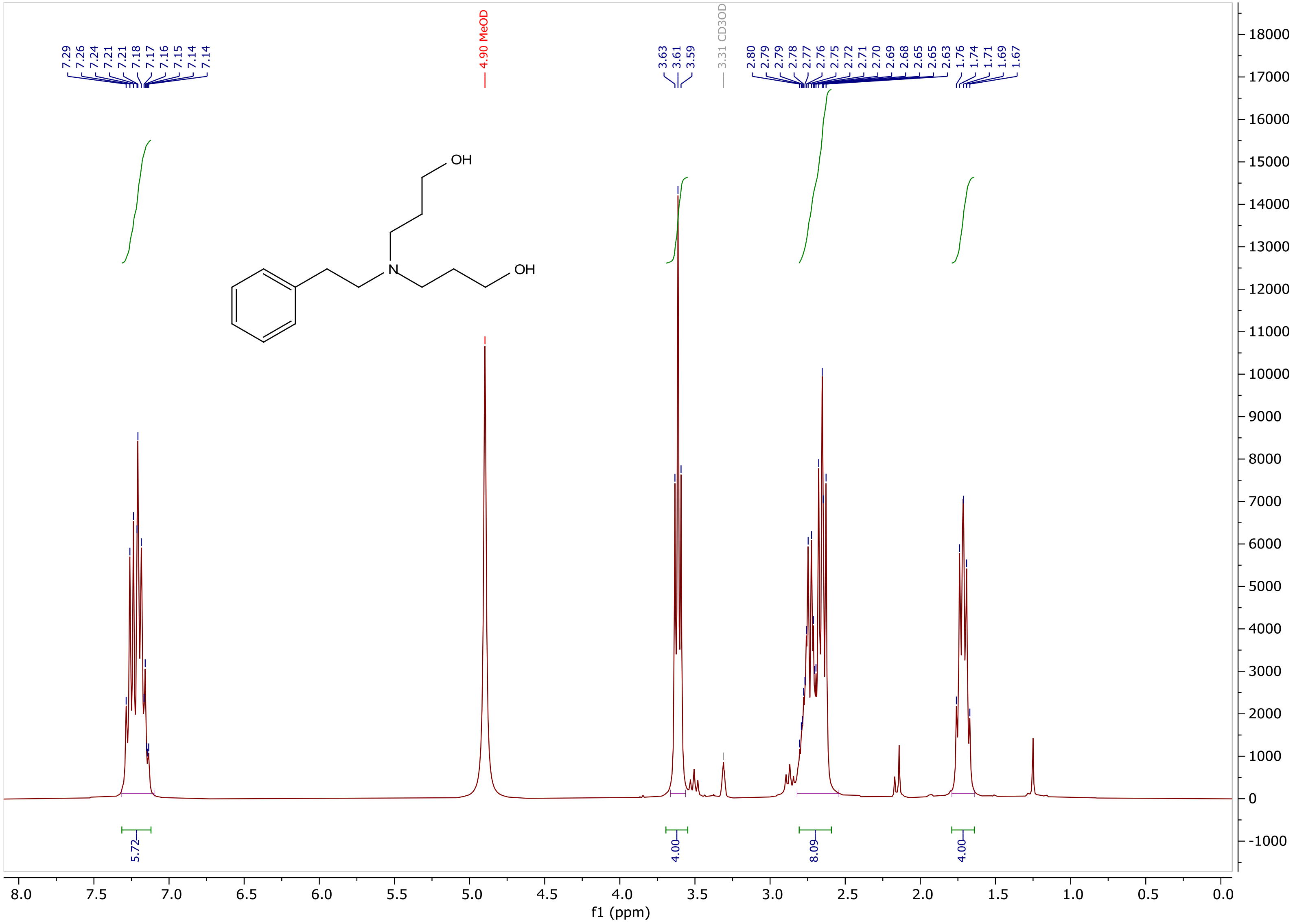

^{1}H NMR (300 MHz, CD_3OD) spectrum of compound **3w.**

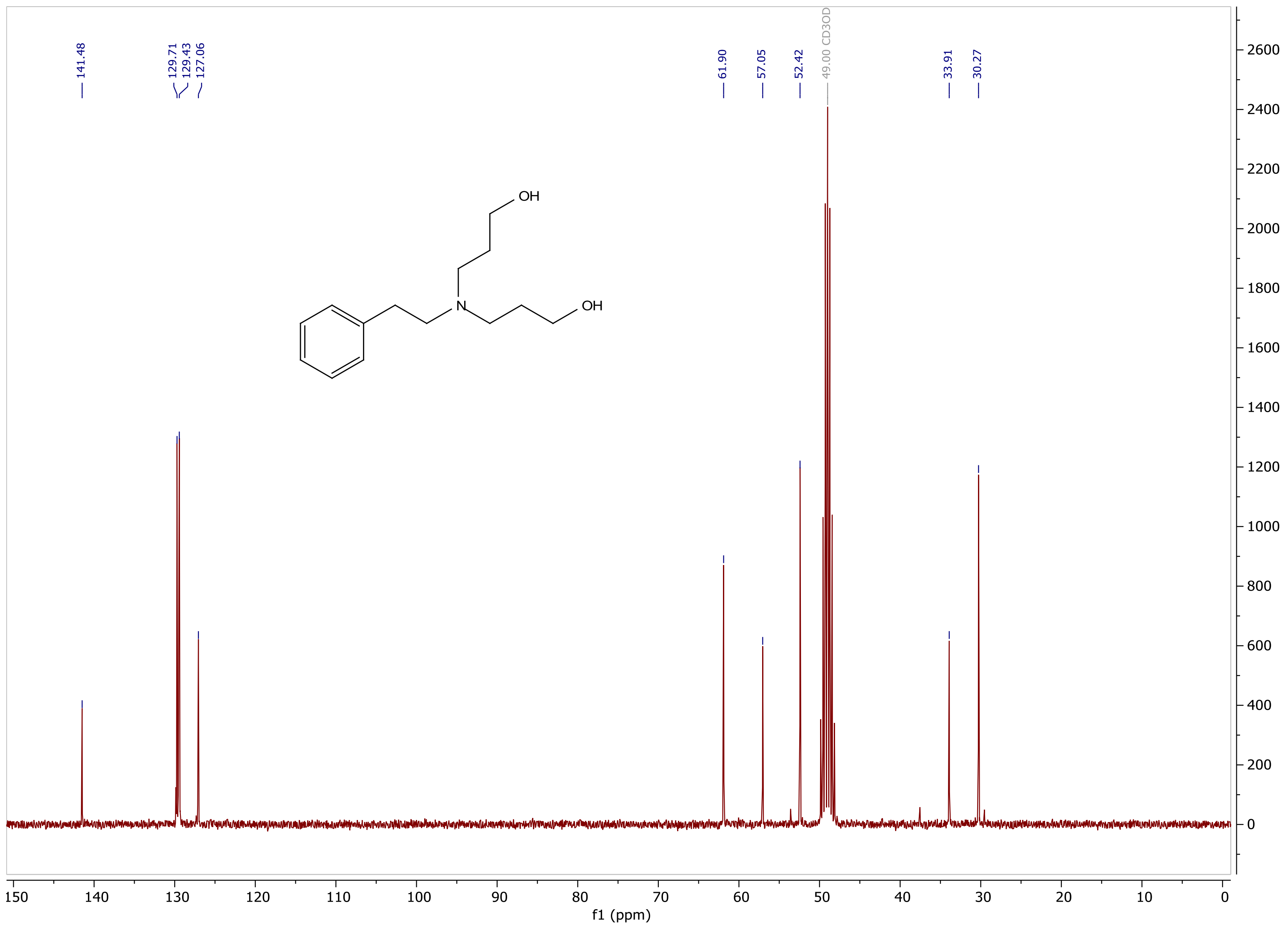

^{13}C NMR (75 MHz, CD_3OD) spectrum of compound **3w.**

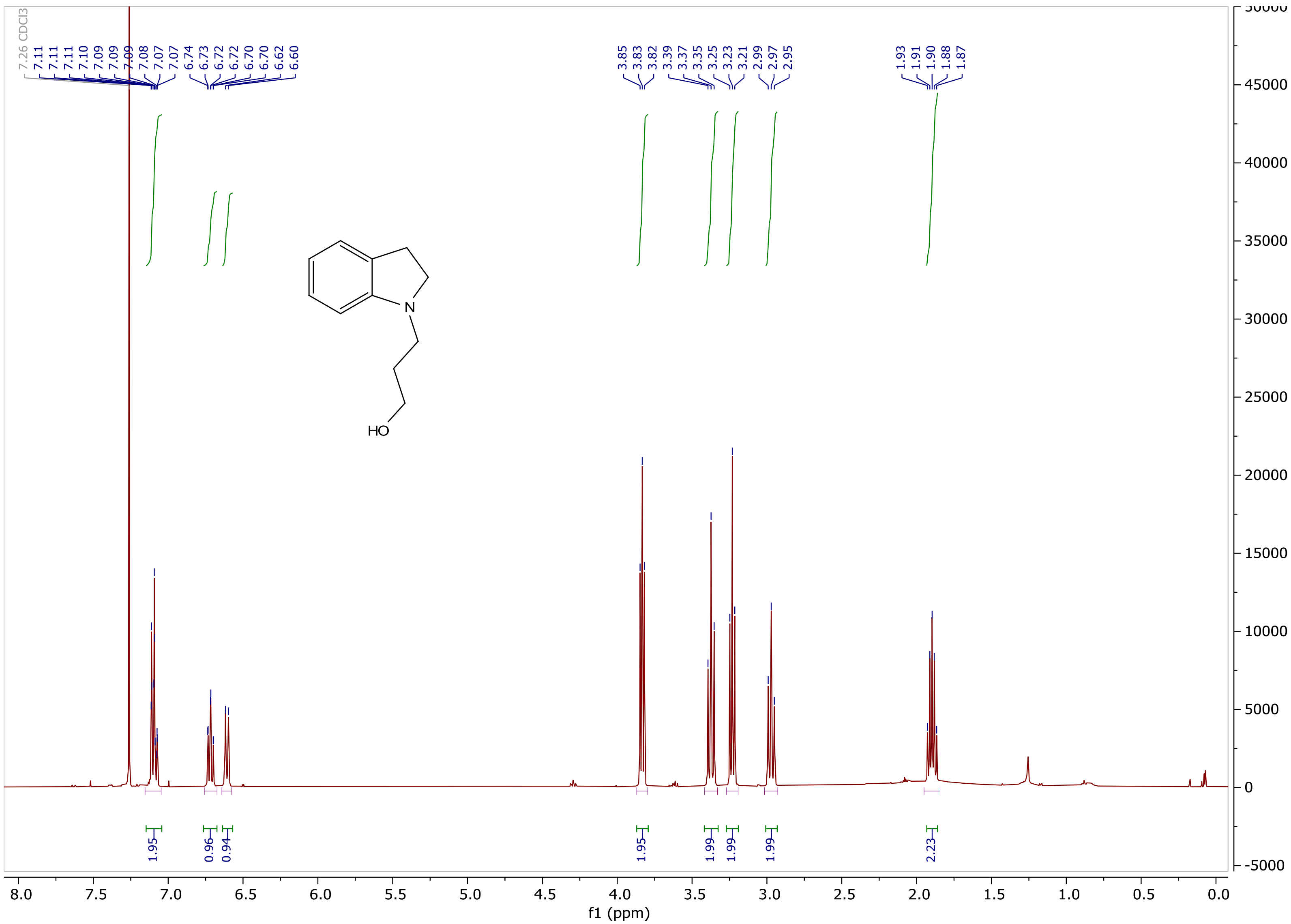

^{1}H NMR (300 MHz, $CDCl_3$) spectrum of compound **3x.**

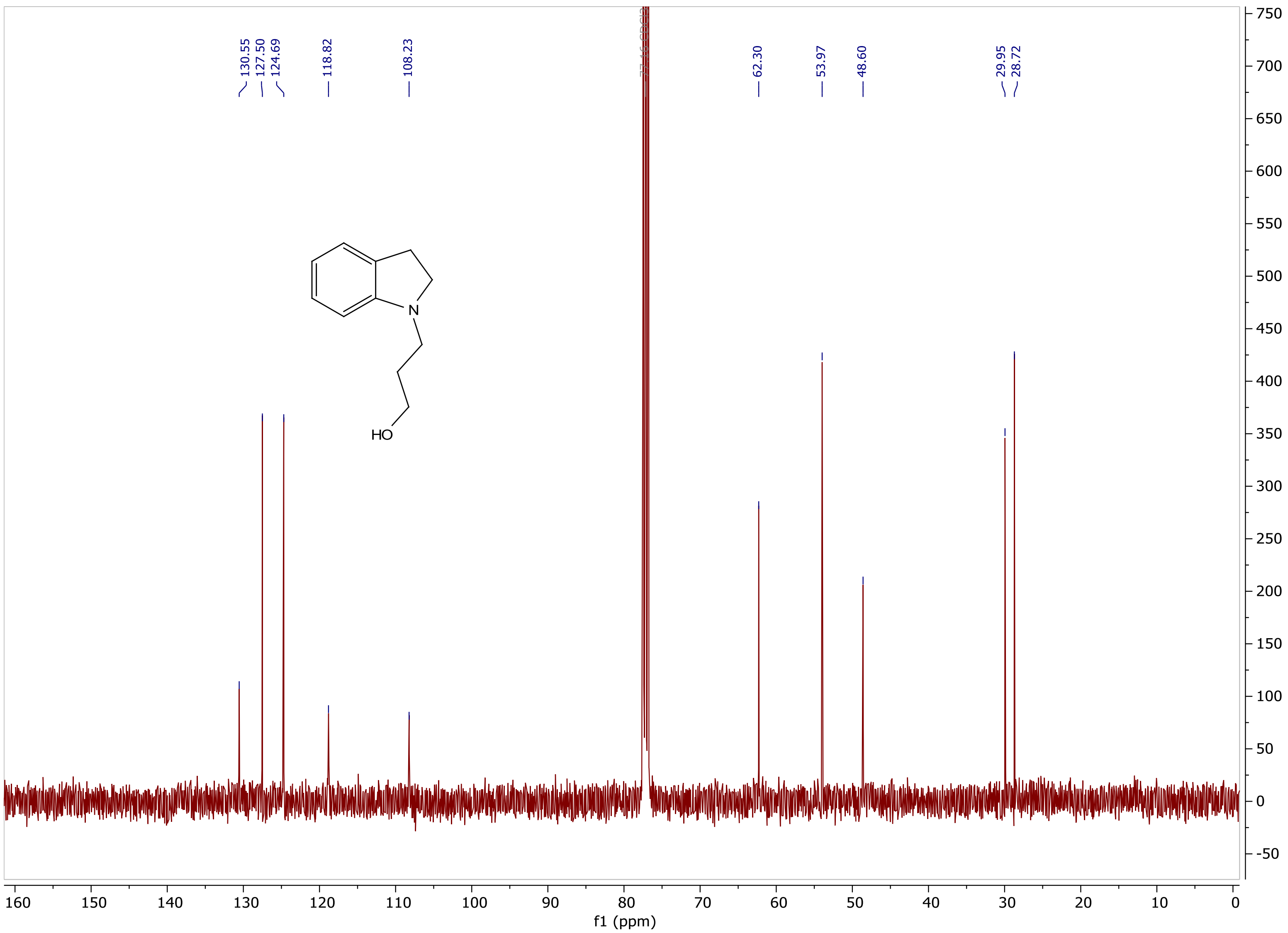

^{13}C NMR (75 MHz, CD_3OD) spectrum of compound **3x.**

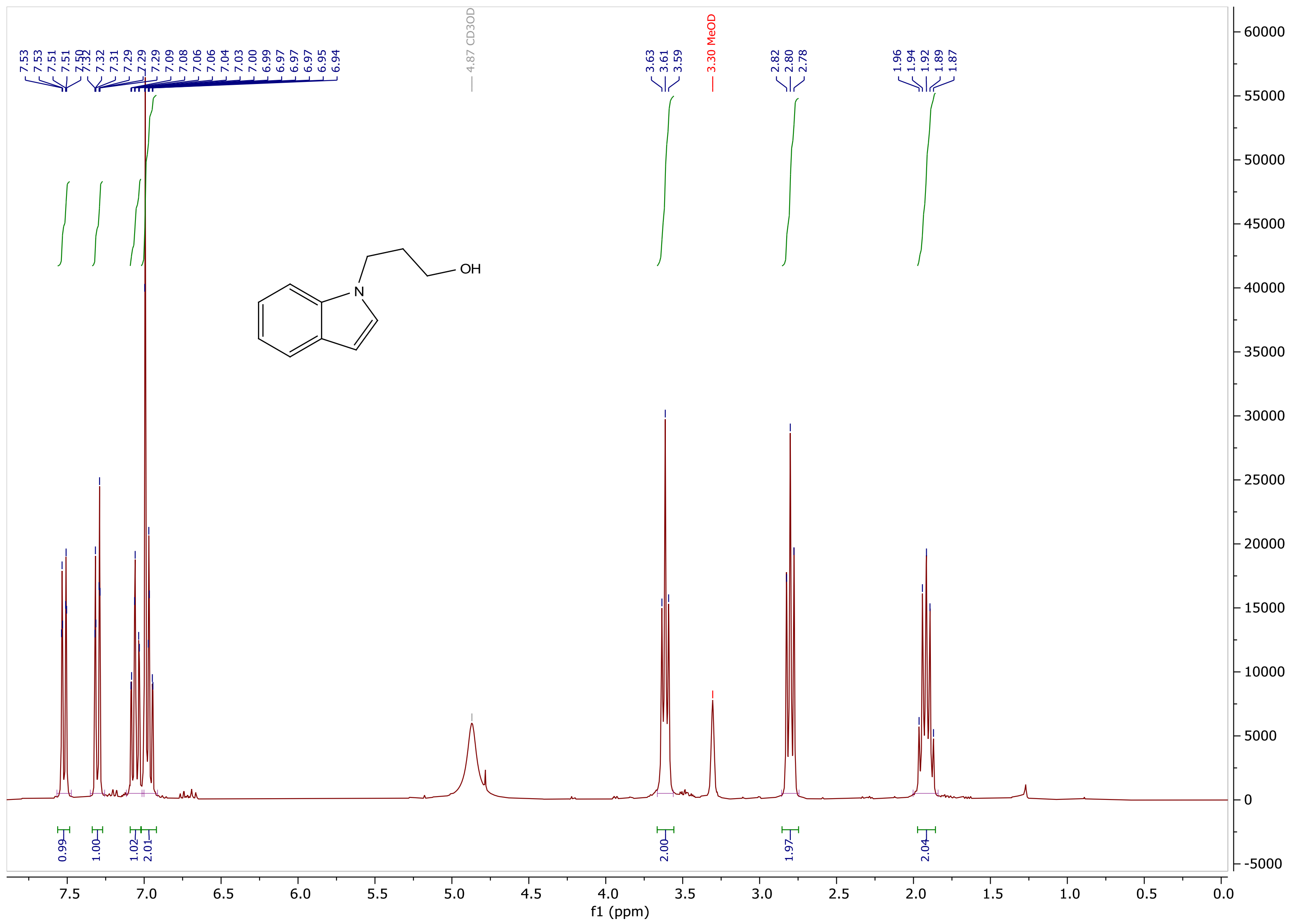

^{1}H NMR (300 MHz, CD_3OD) spectrum of compound **3y.**

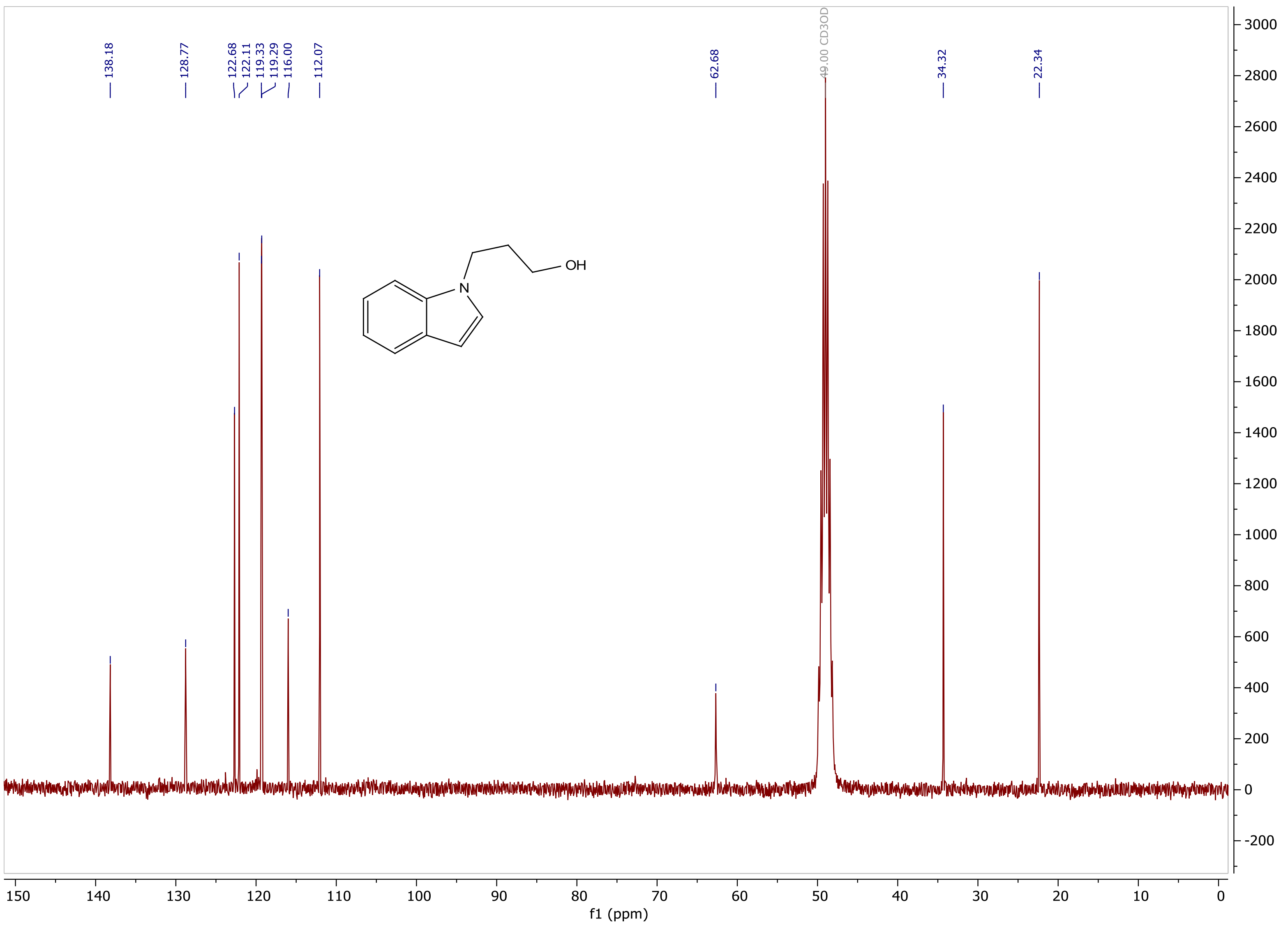

^{13}C NMR (75 MHz, CD_3OD) spectrum of compound **3y.**

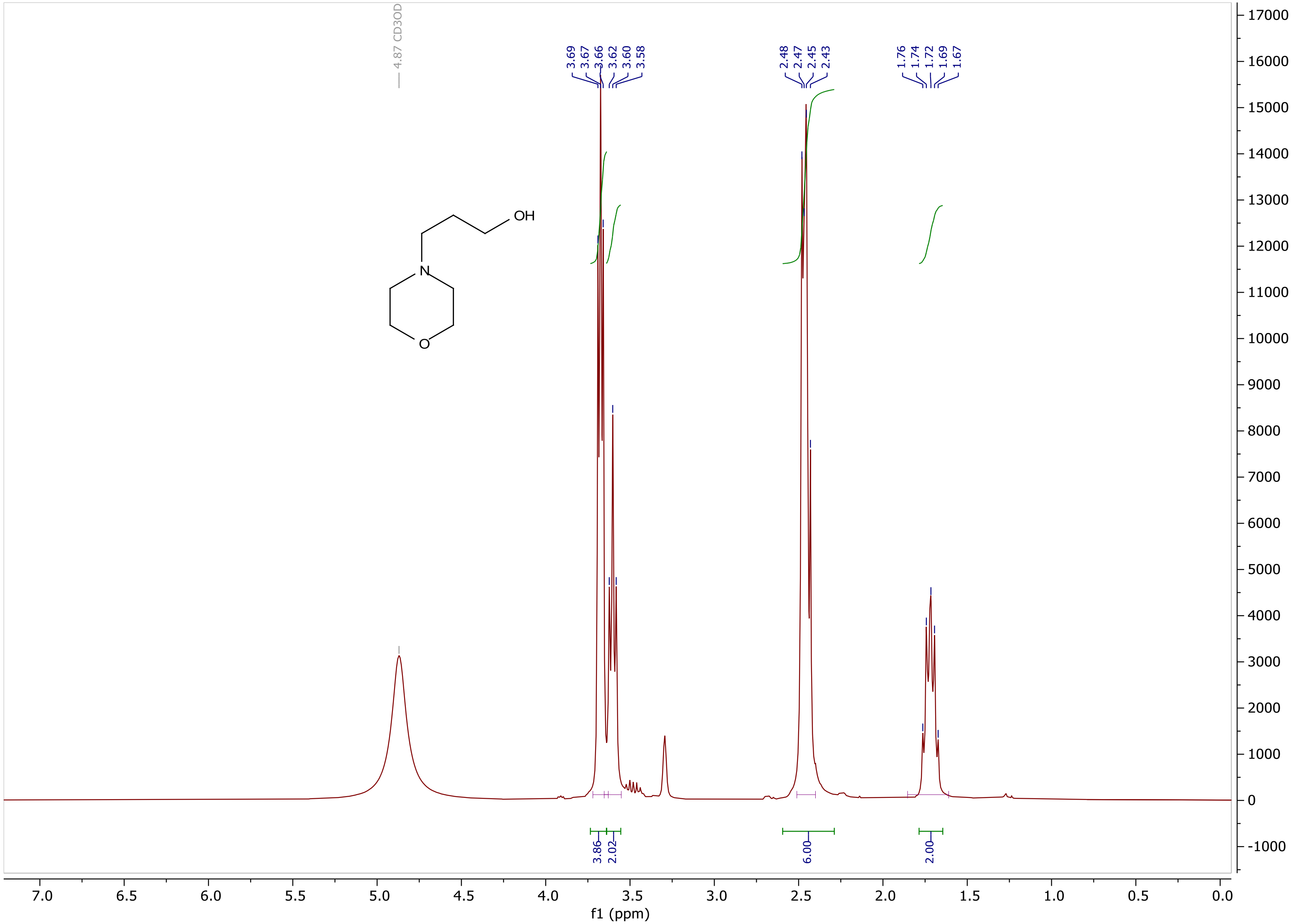

^{1}H NMR (300 MHz, CD_3OD) spectrum of compound **3z.**

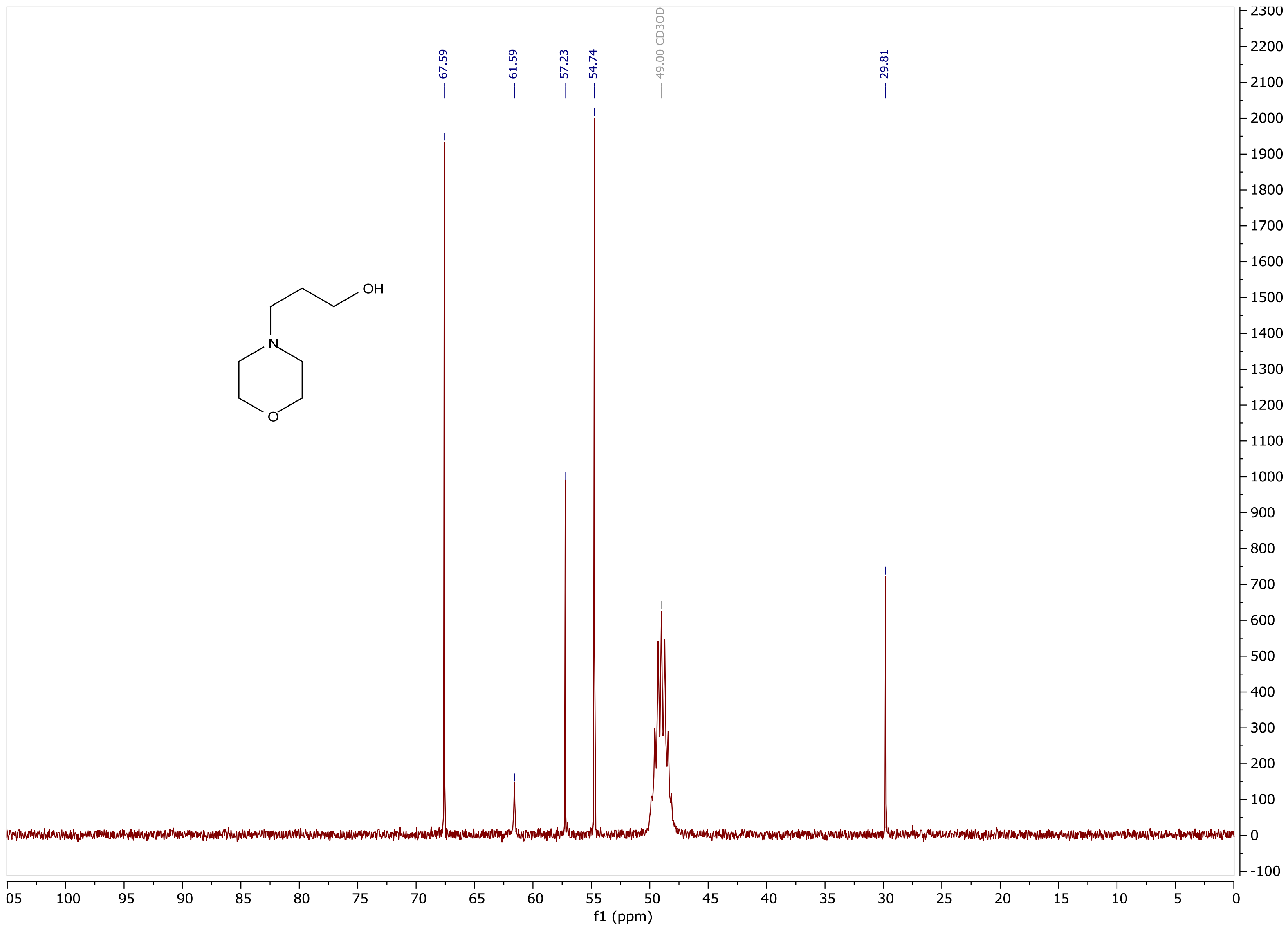

^{13}C NMR (75 MHz, CD_3OD) spectrum of compound **3z.**

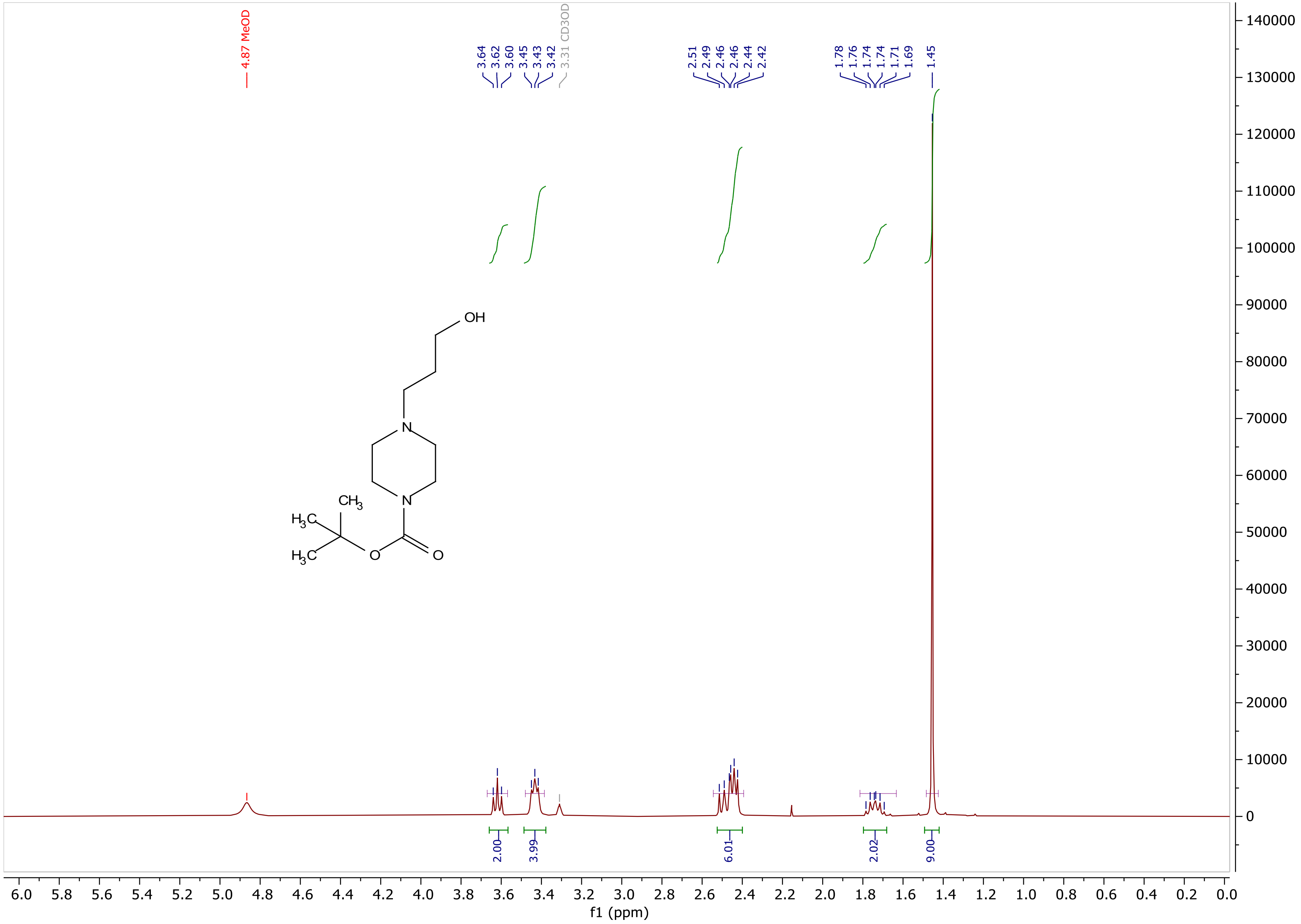

^{1}H NMR (300 MHz, CD_3OD) spectrum of compound **3aa.**

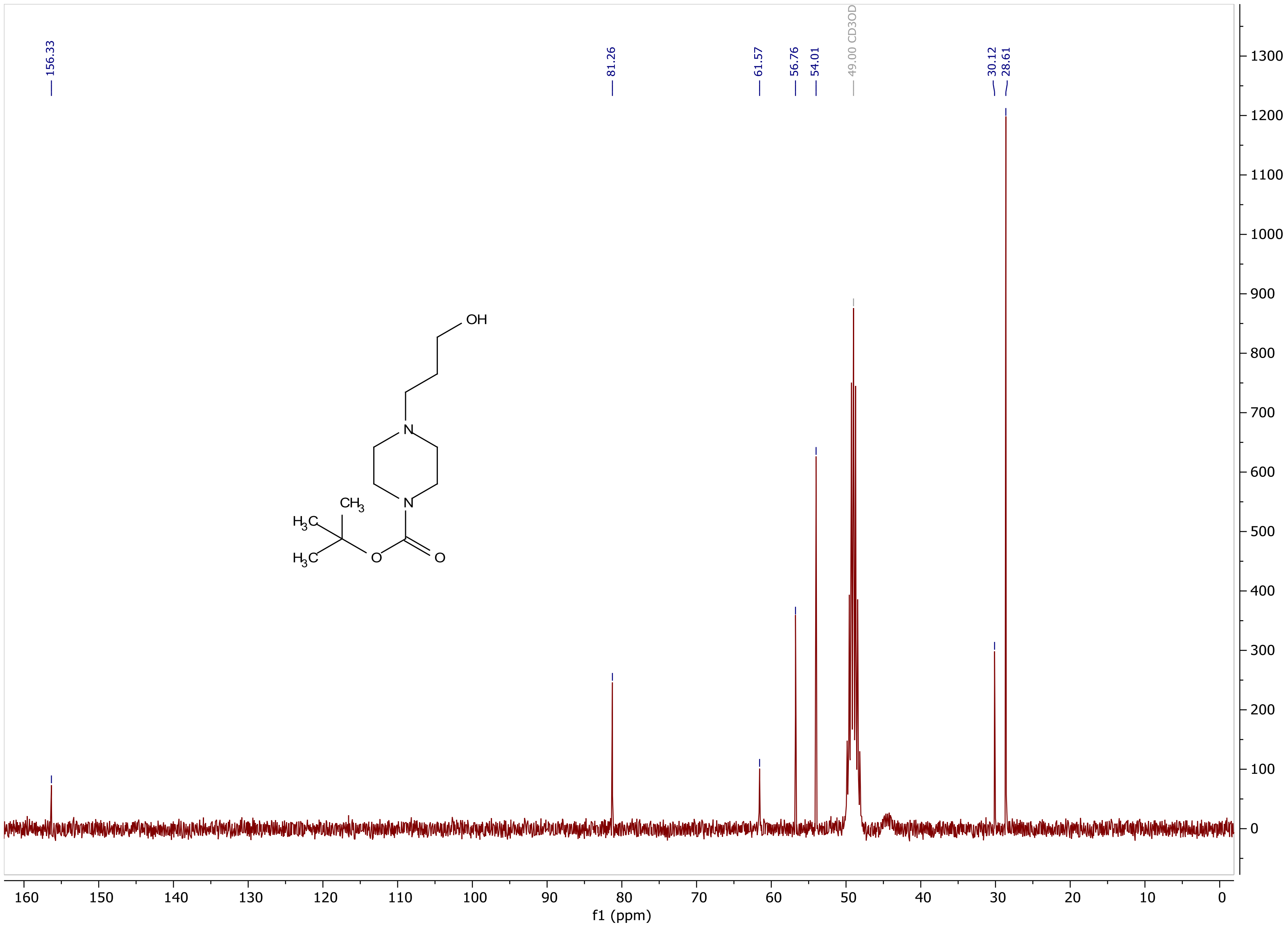

^{13}C NMR (75 MHz, CD_3OD) spectrum of compound **3aa.**

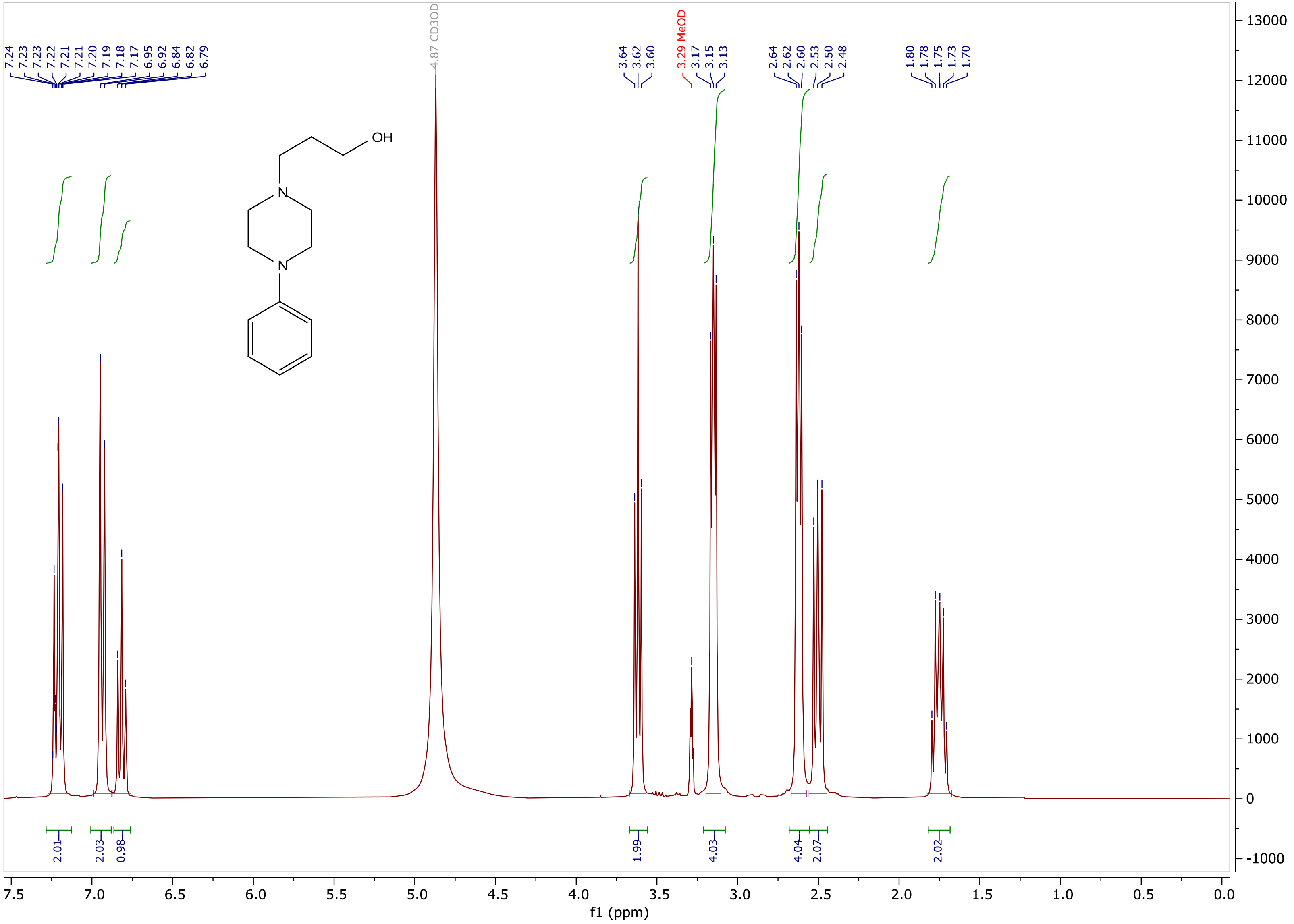

^{1}H NMR (300 MHz, CD_3OD) spectrum of compound **3ab.**

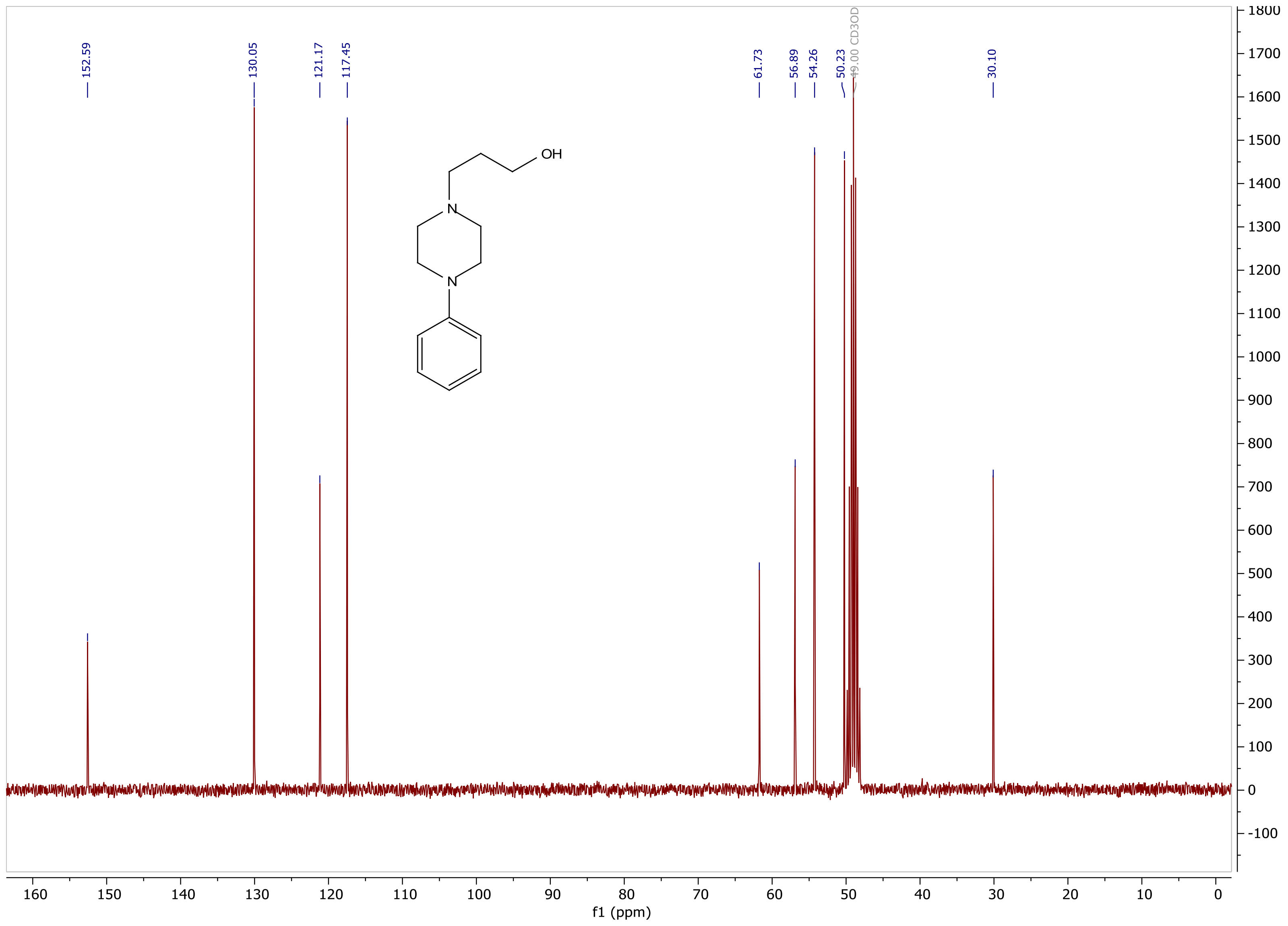

^{13}C NMR (75 MHz, CD_3OD) spectrum of compound **3ab.**

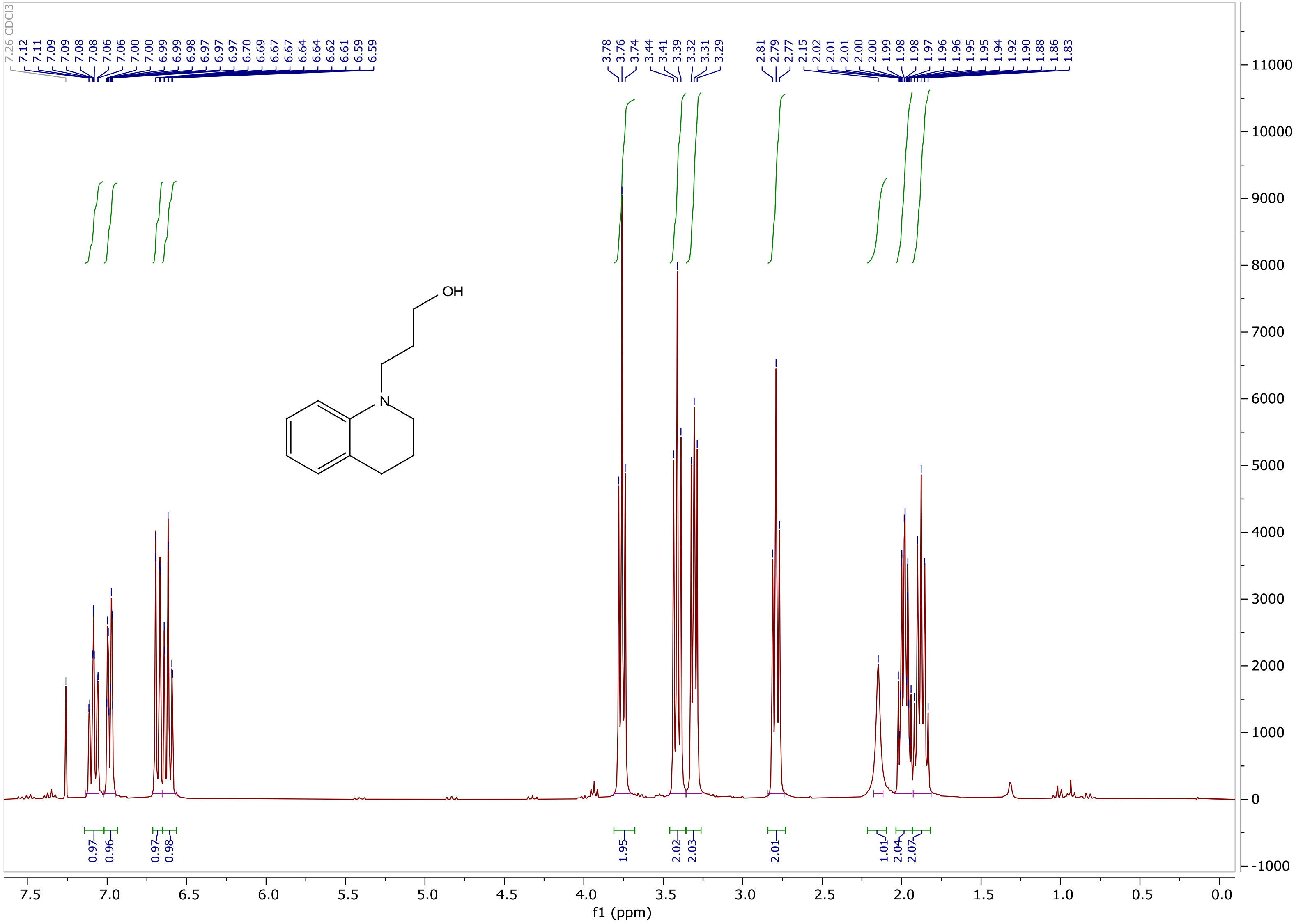

^{1}H NMR (300 MHz, $CDCl_3$) spectrum of compound **3ac.**

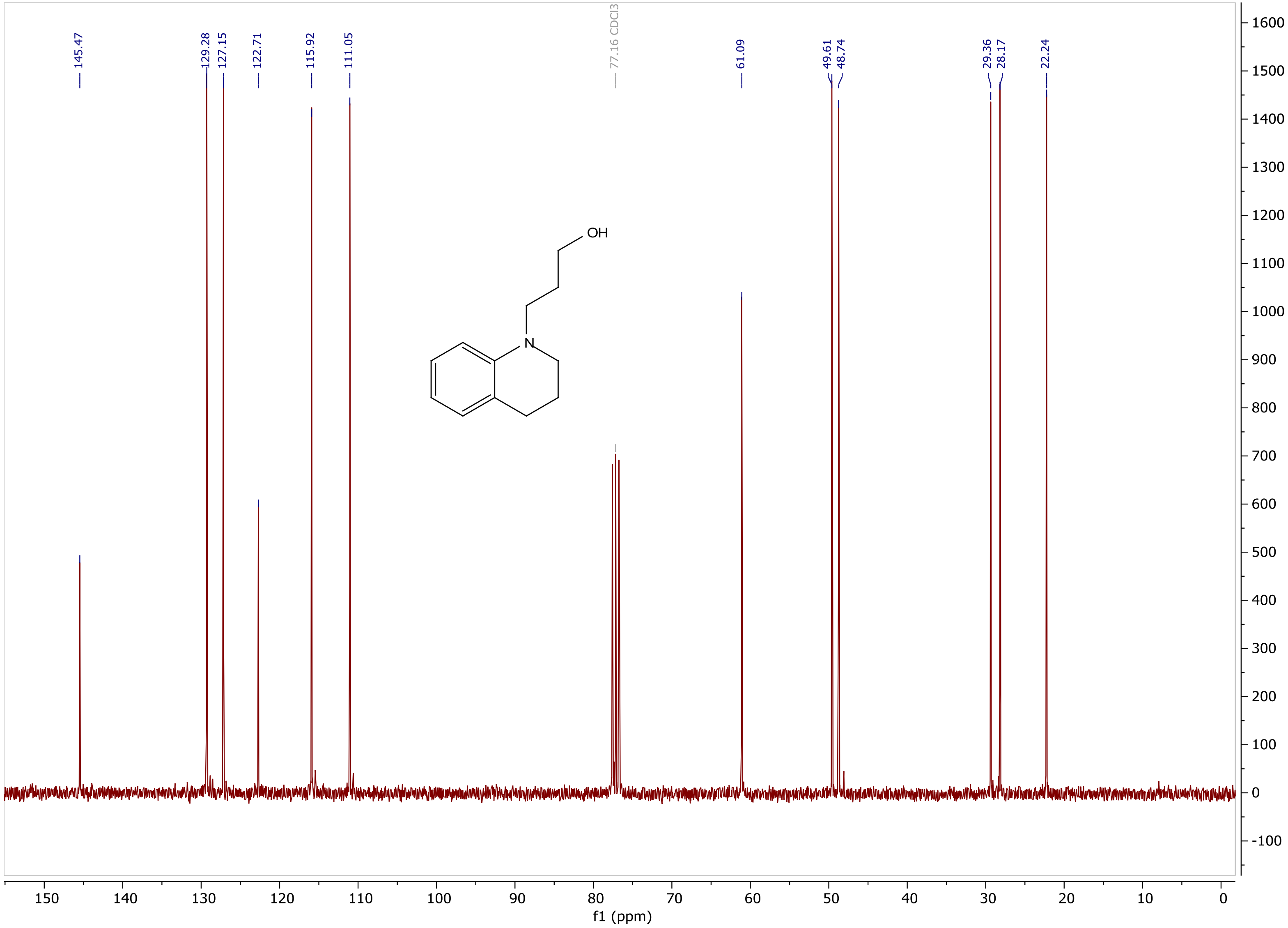

^{13}C NMR (75 MHz, $CDCl_3$) spectrum of compound **3ac.**

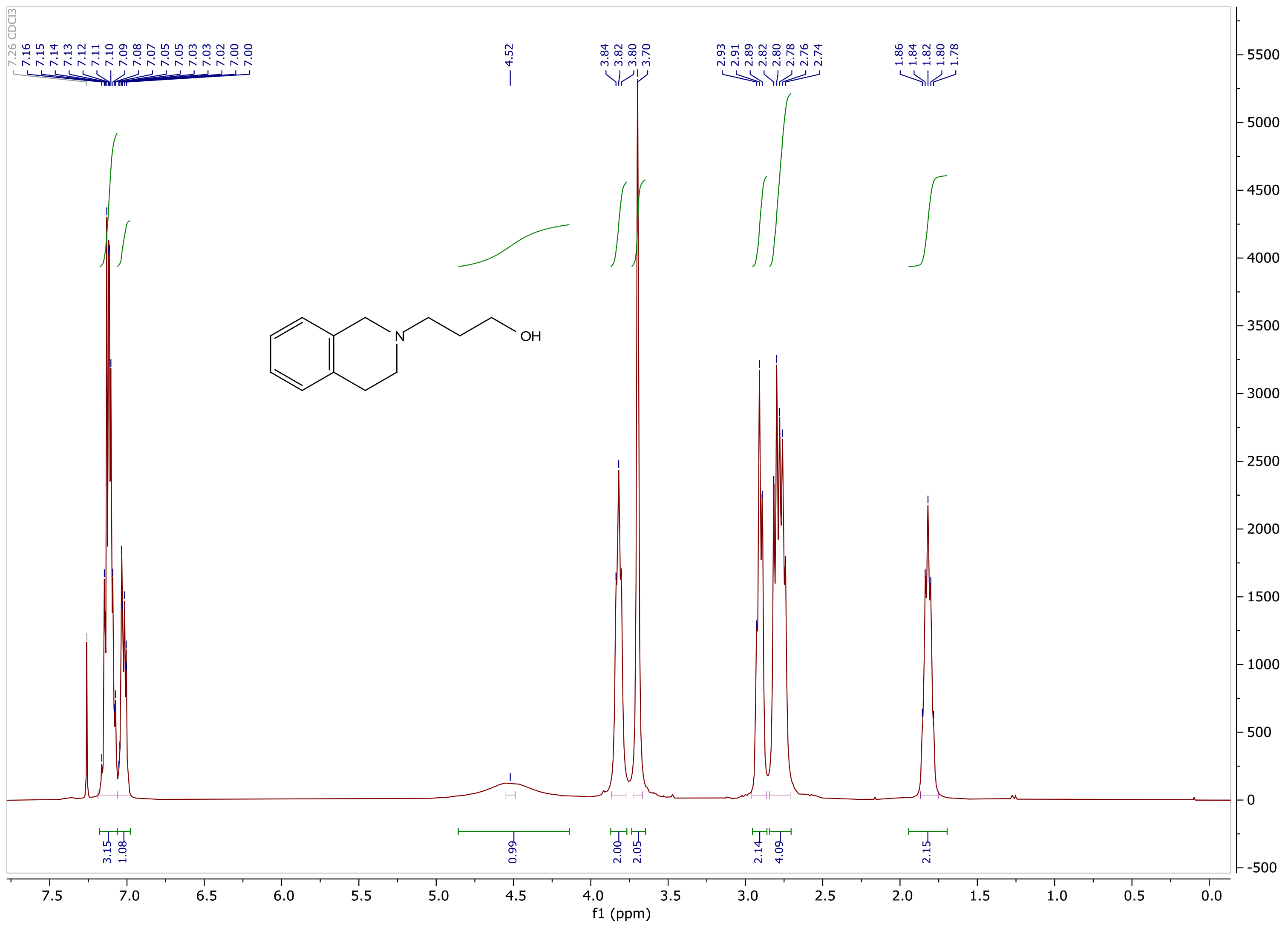

^{1}H NMR (300 MHz, $CDCl_3$) spectrum of compound **3ad.**

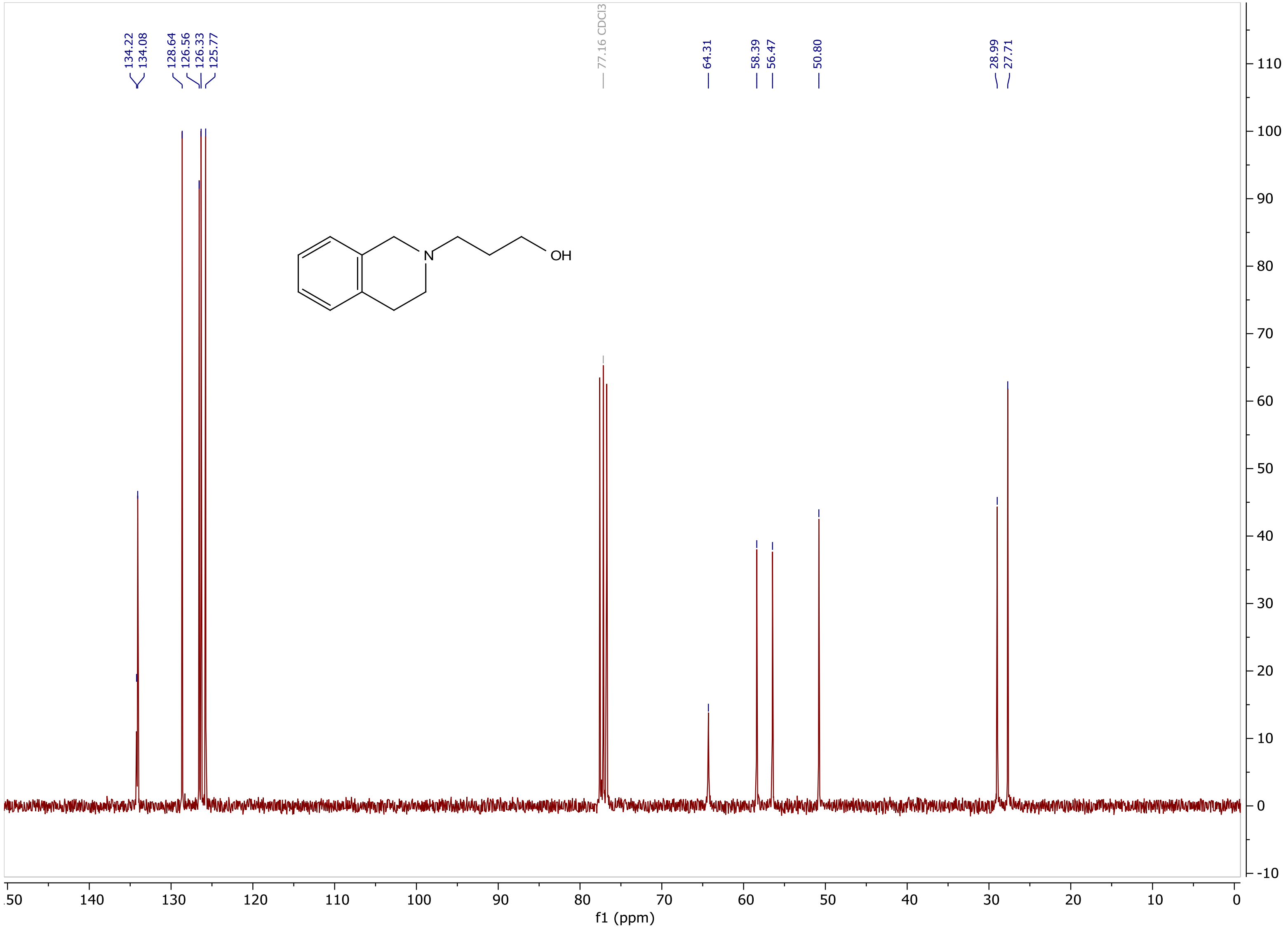

^{13}C NMR (75 MHz, $CDCl_3$) spectrum of compound **3ad.**

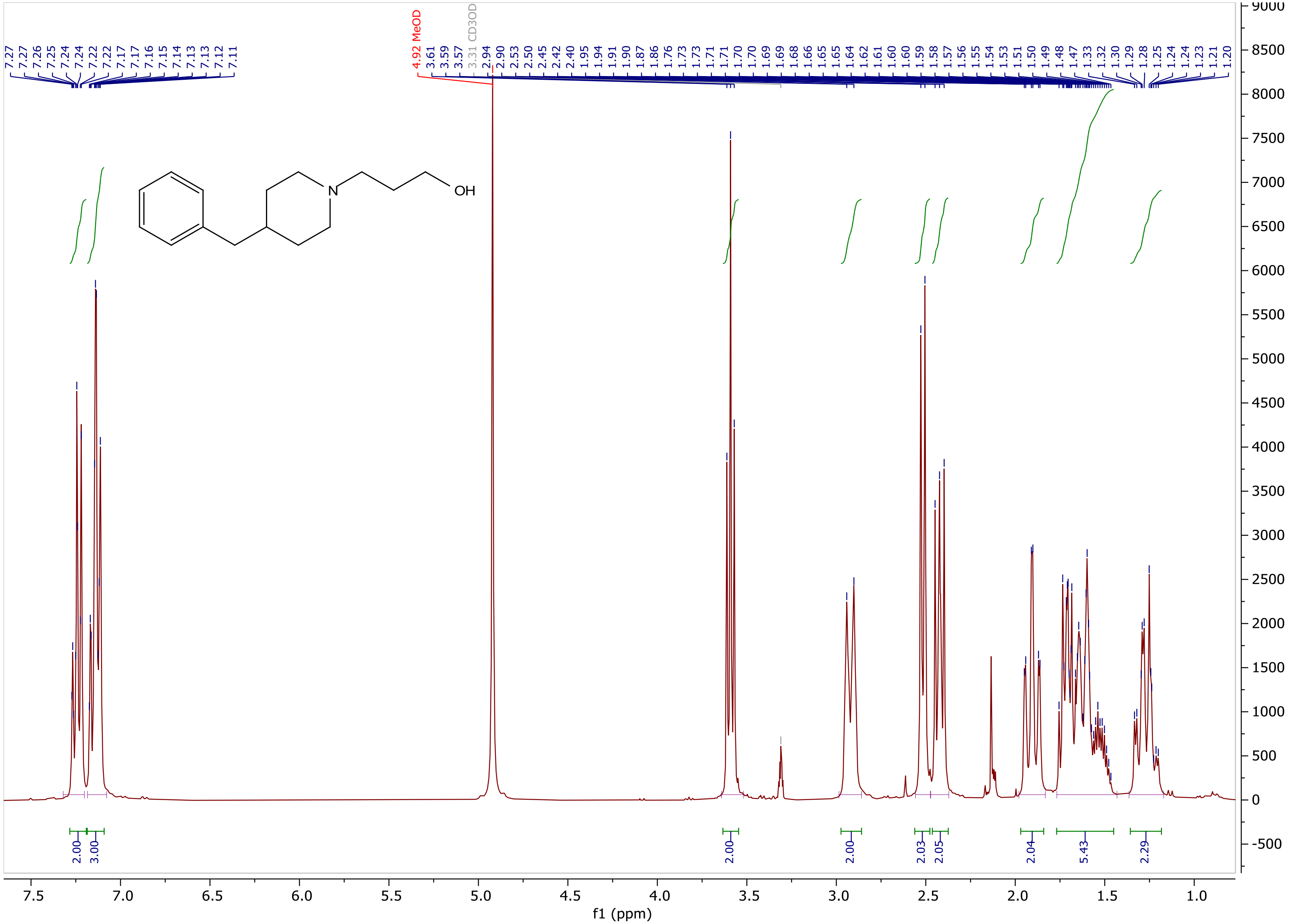

^{1}H NMR (300 MHz, CD_3OD) spectrum of **3ae**

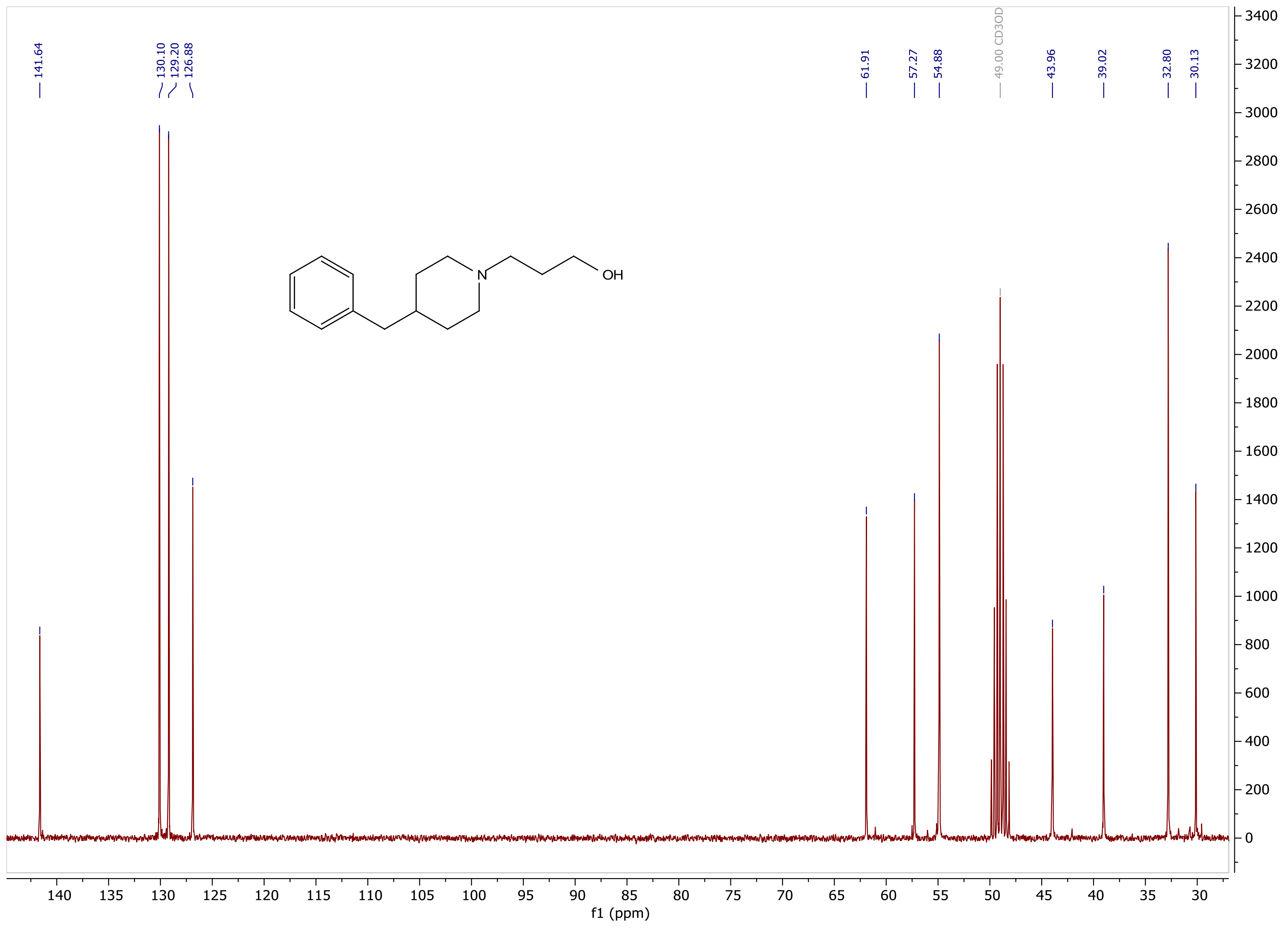

^{13}C NMR (75 MHz, CD_3OD) spectrum of compound **3ae.**

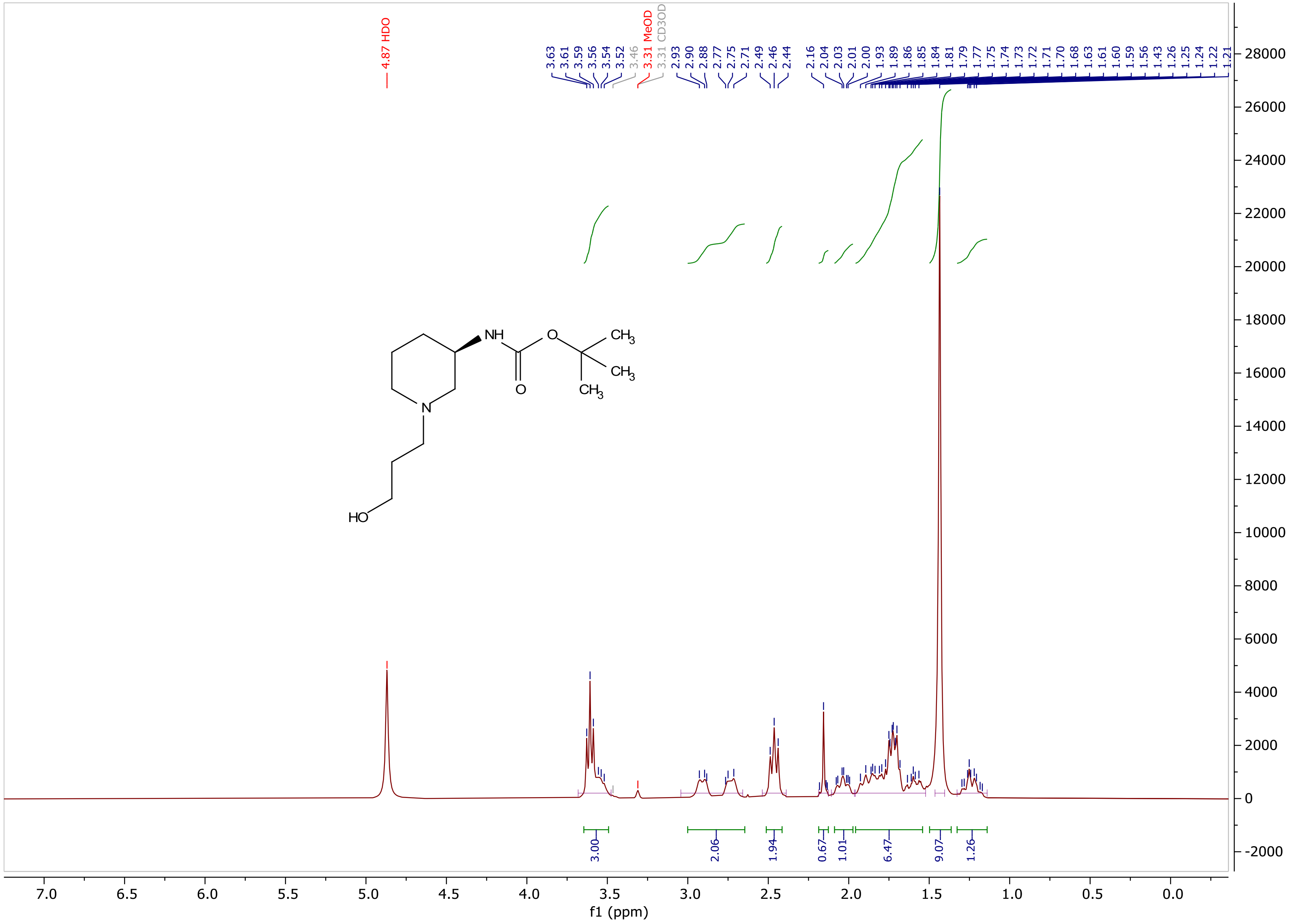

^{1}H NMR (300 MHz, CD_3OD) spectrum of compound **3af.**

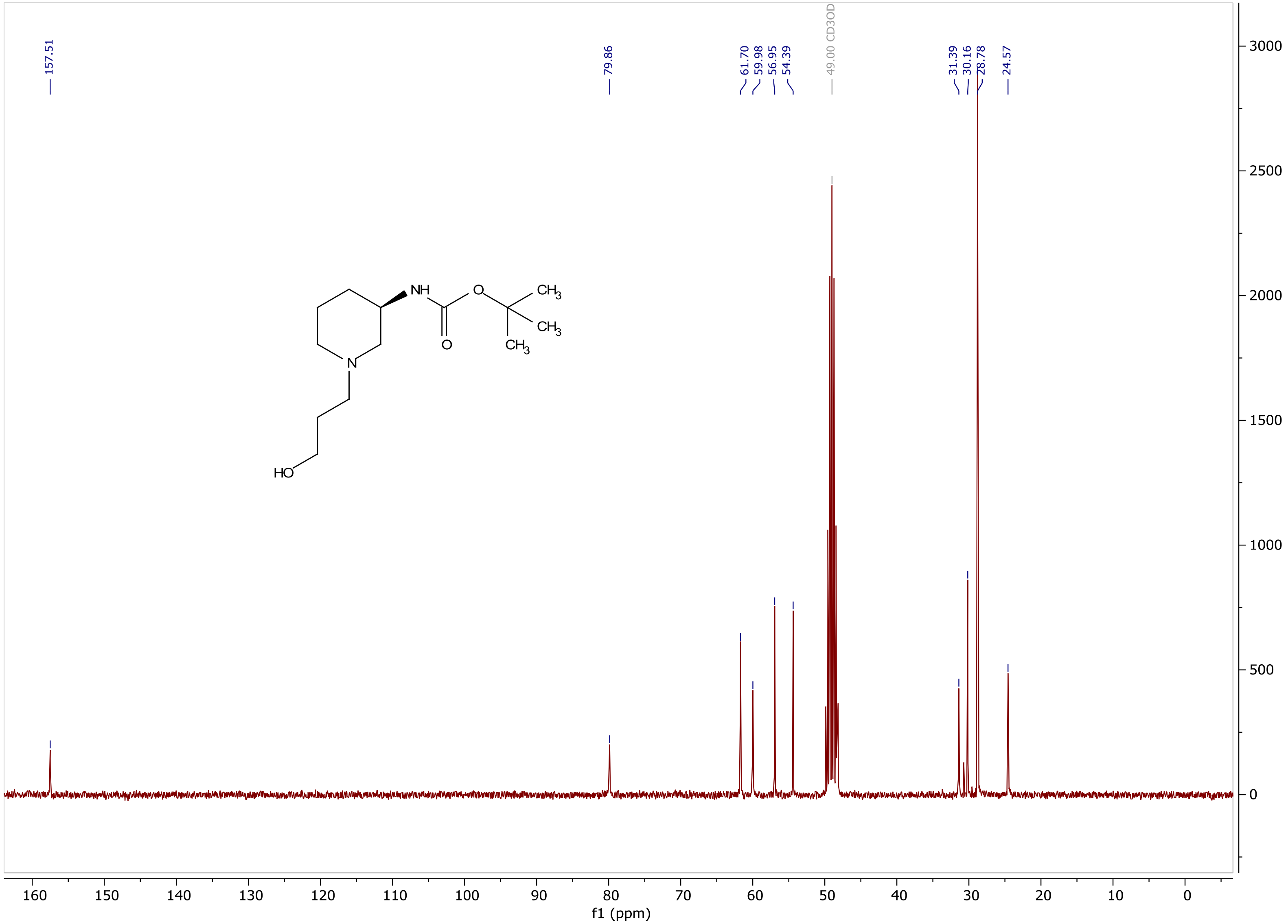

^{13}C NMR (75 MHz, CD_3OD) spectrum of compound **3af**

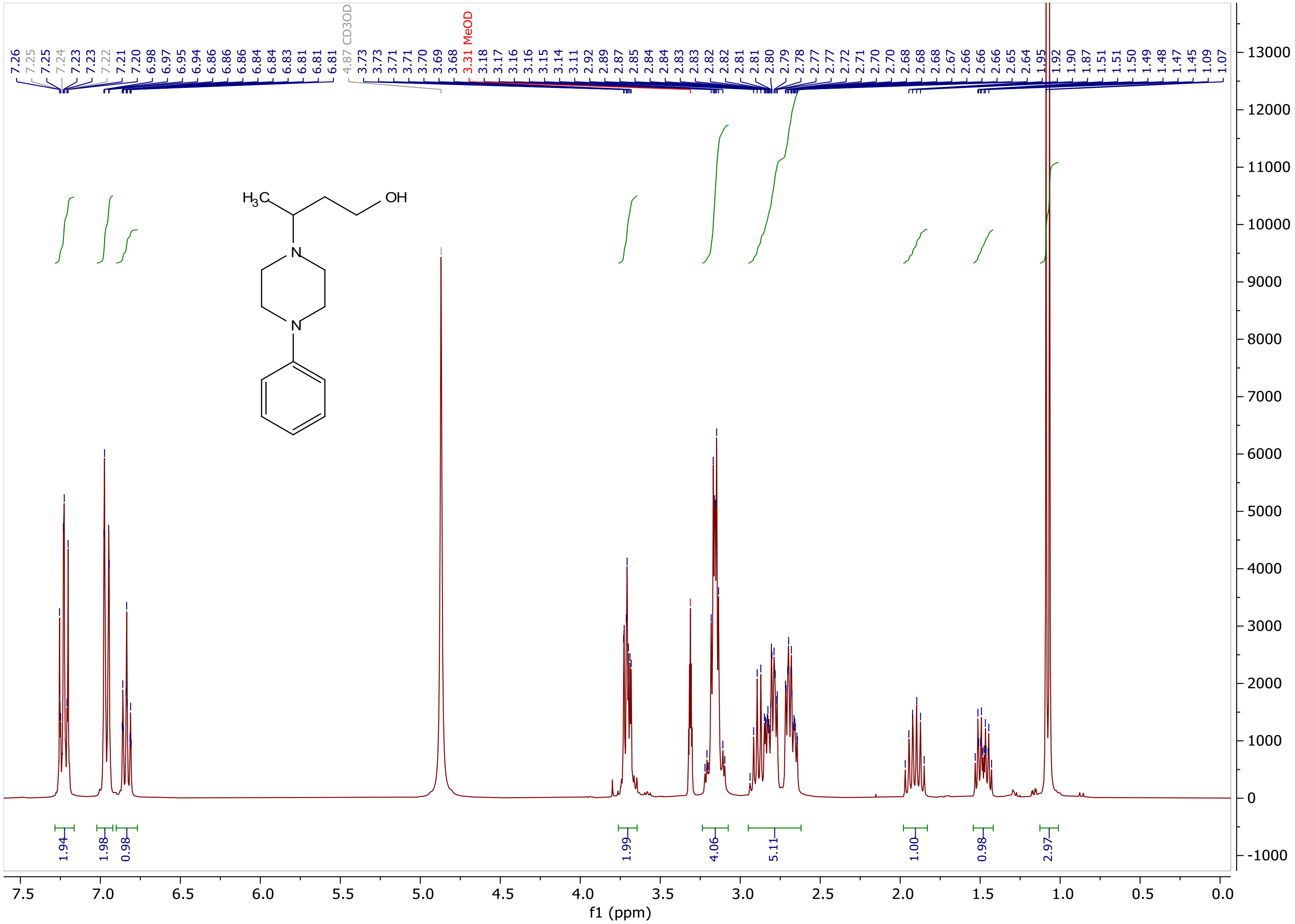

^{1}H NMR (300 MHz, $CDCl_3$) spectrum of compound **5a**.

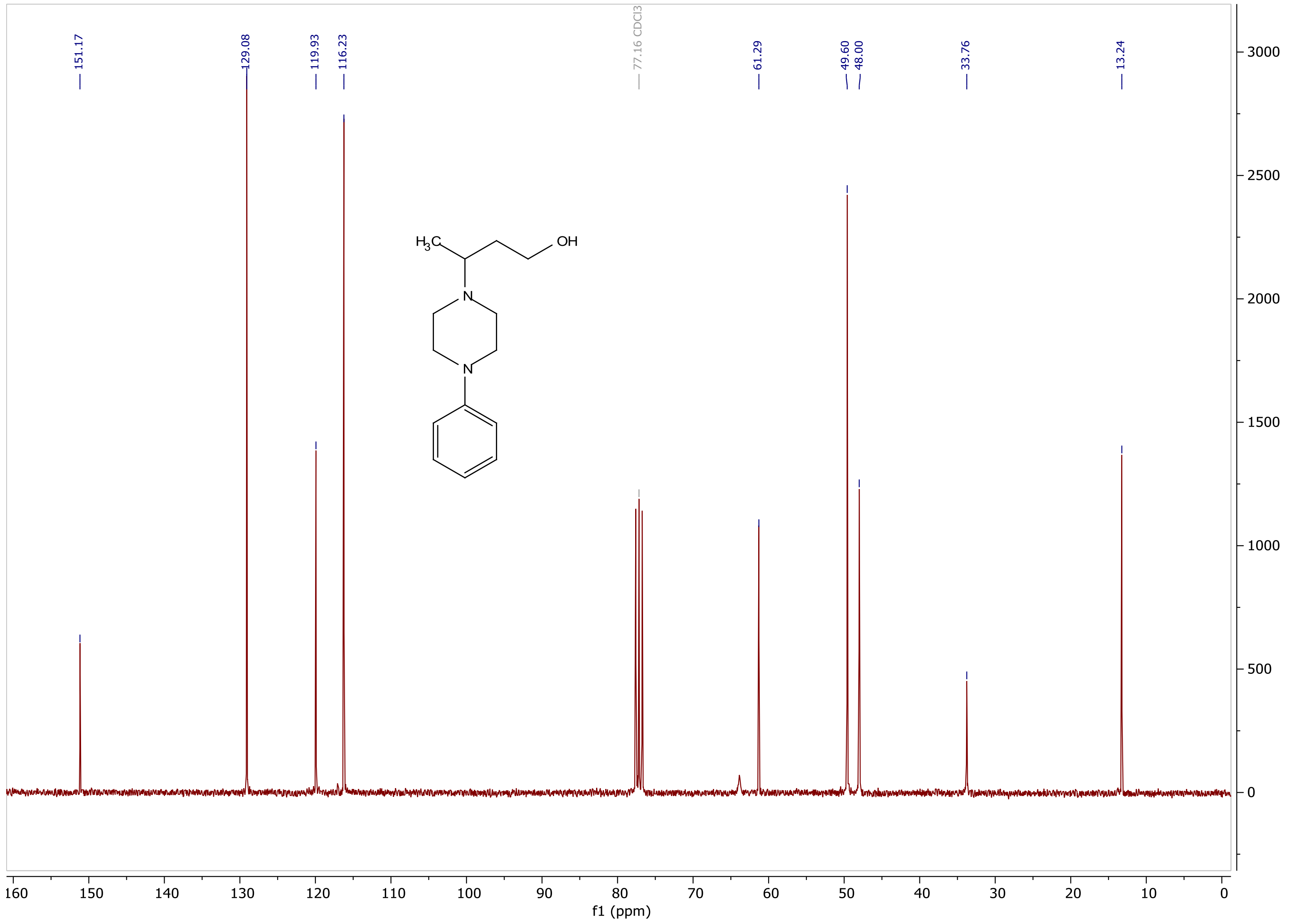

^{13}C NMR (75 MHz, $CDCl_3$) spectrum of compound **5a**.

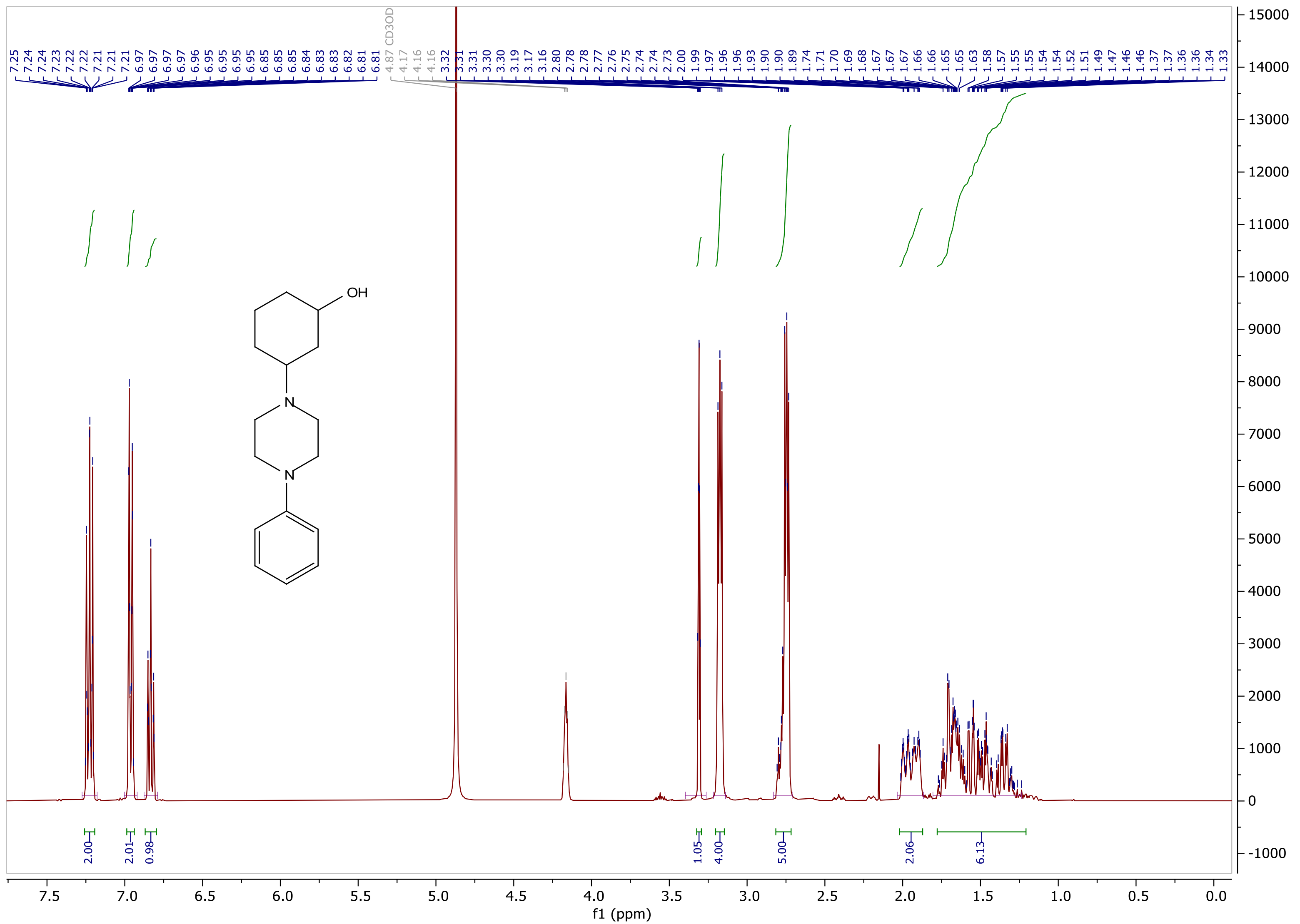

^{1}H NMR (300 MHz, CD_3OD) spectrum of compound **5b**.

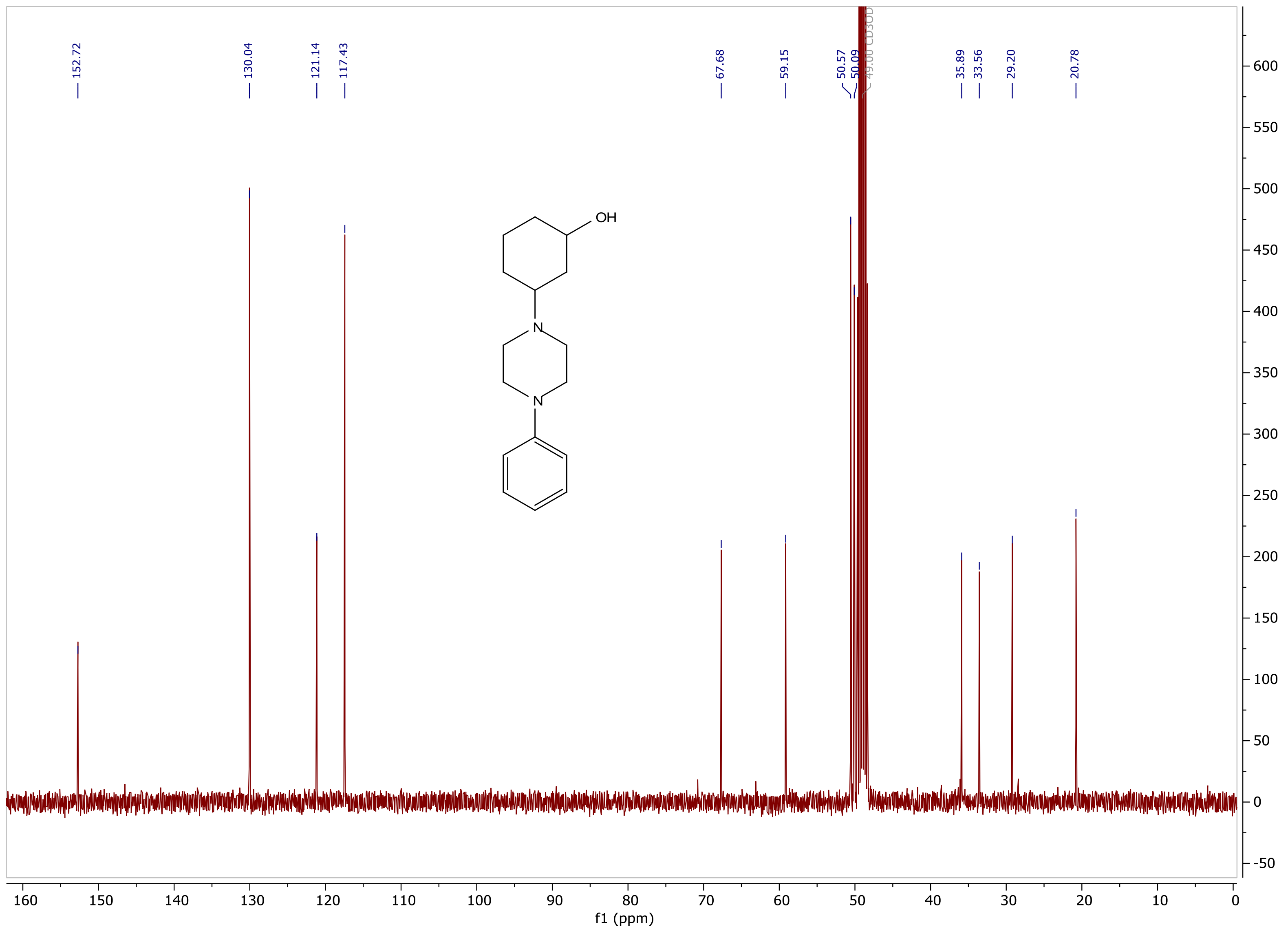

^{13}C NMR (75 MHz, CD_3OD) spectrum of compound **5b**.

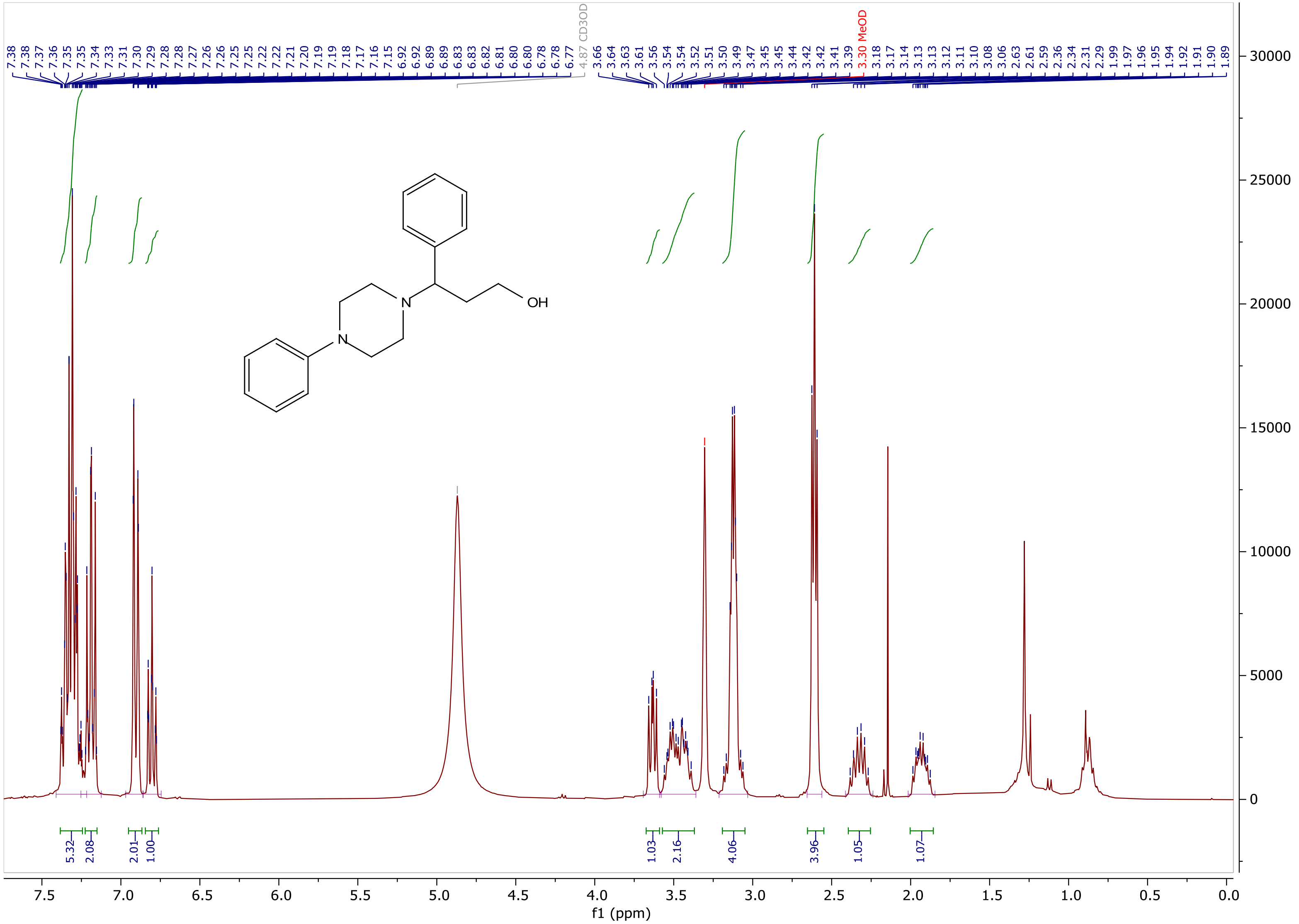

^{1}H NMR (300 MHz, CD_3OD) spectrum of compound **5c.**

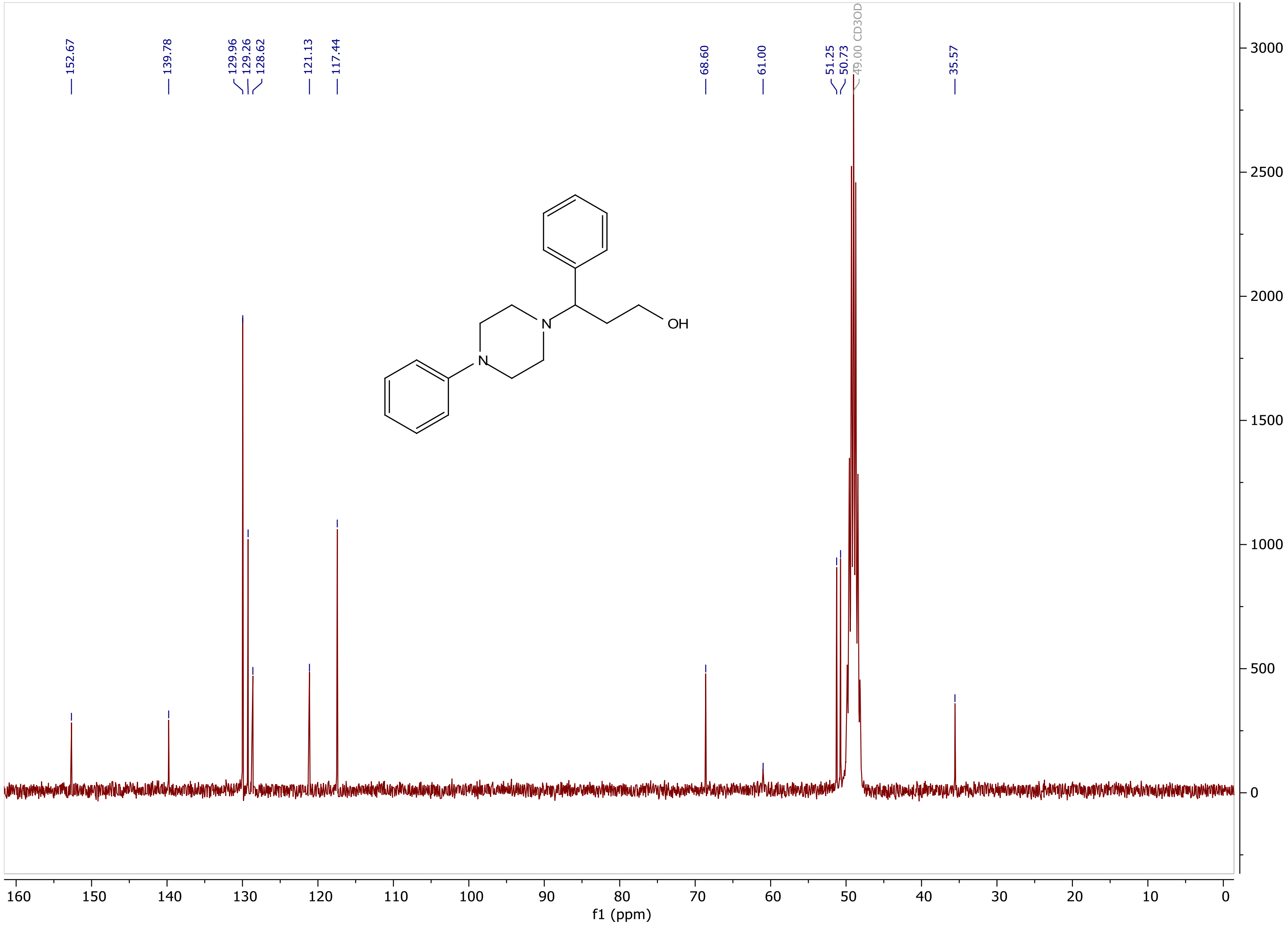

^{13}C NMR (75 MHz, CD_3OD) spectrum of compound **5c**.

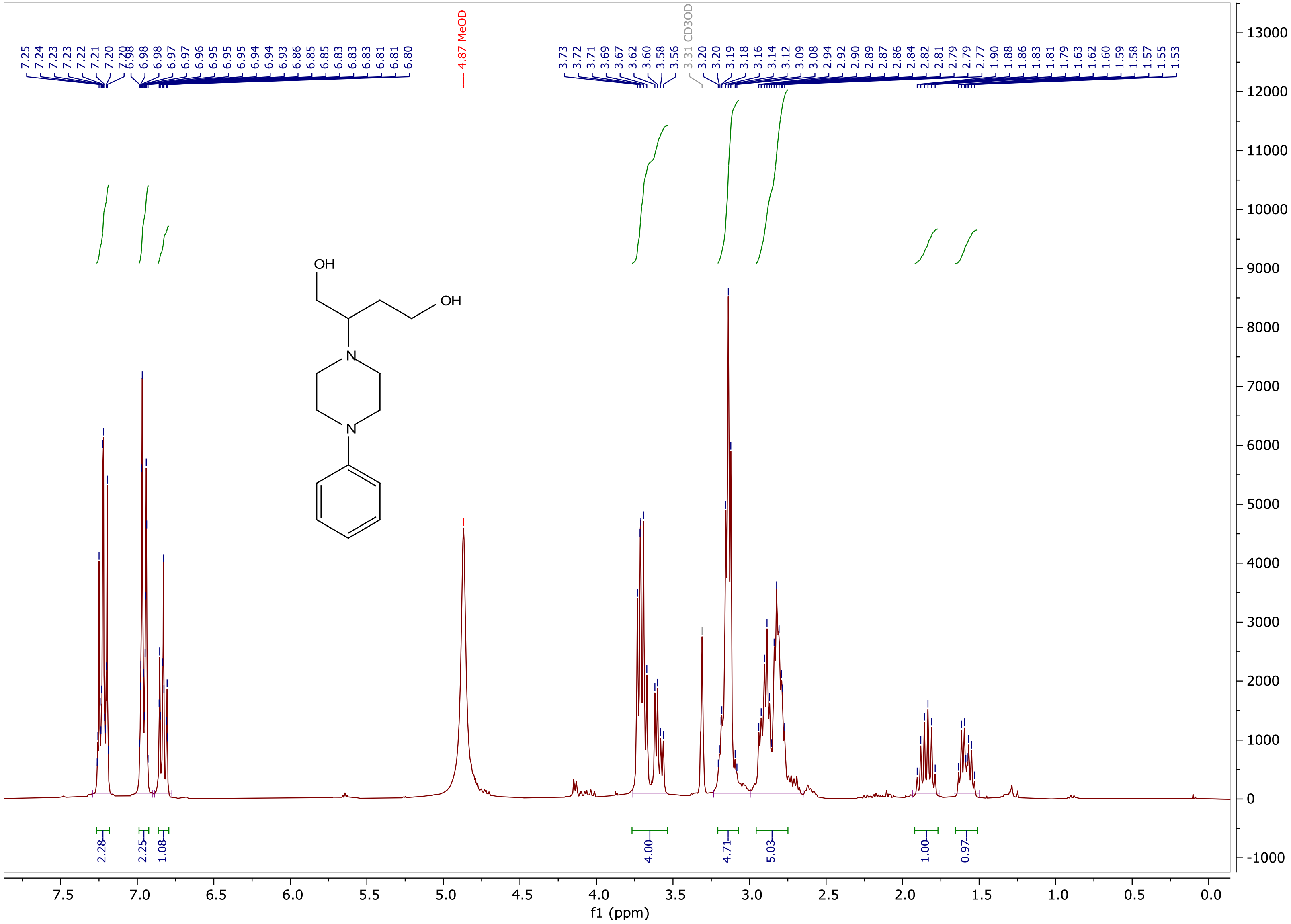

^{1}H NMR (300 MHz, CD_3OD) spectrum of compound **5d**.

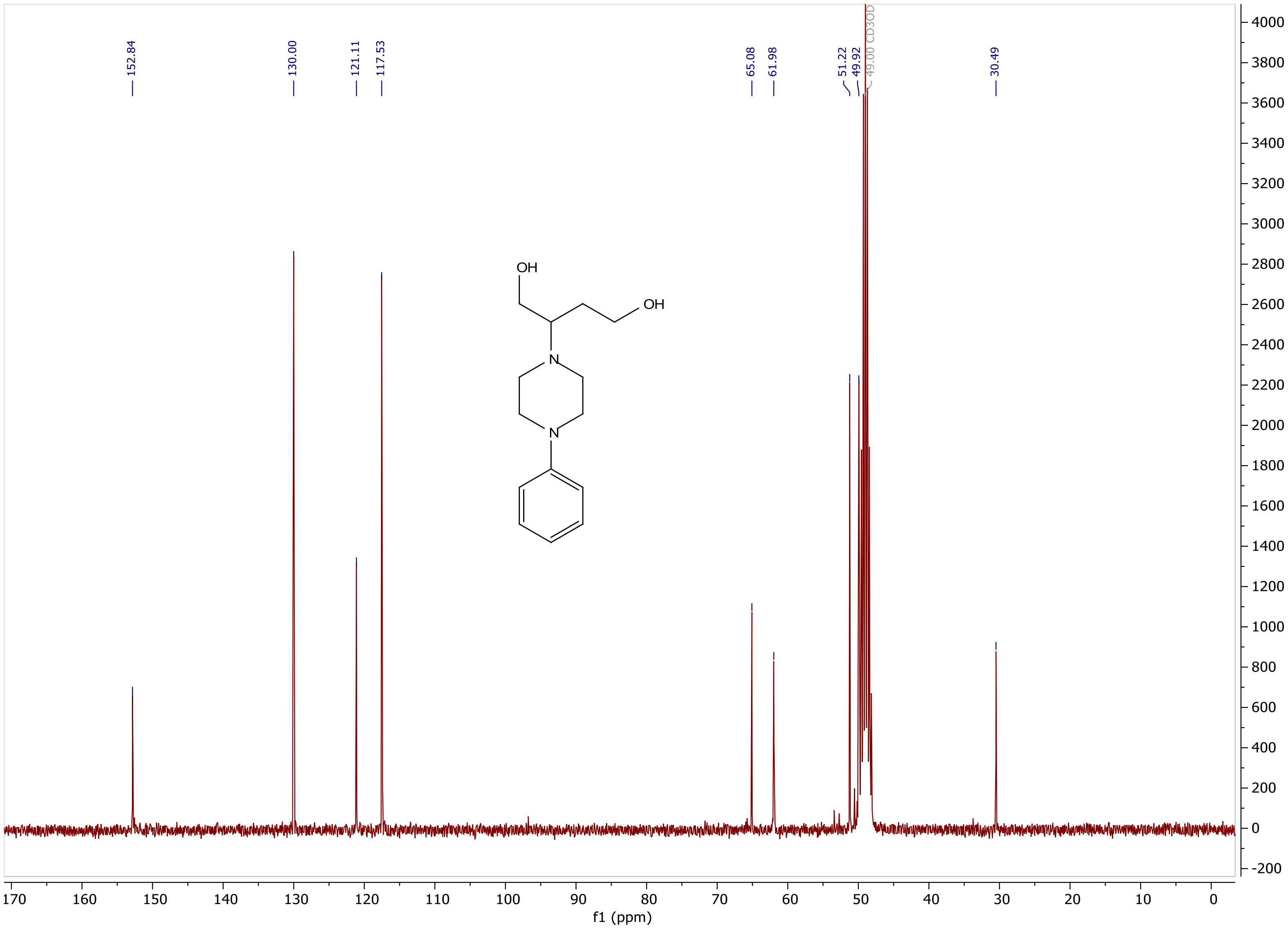

^{13}C NMR (75 MHz, CD_3OD) spectrum of compound **5d**.

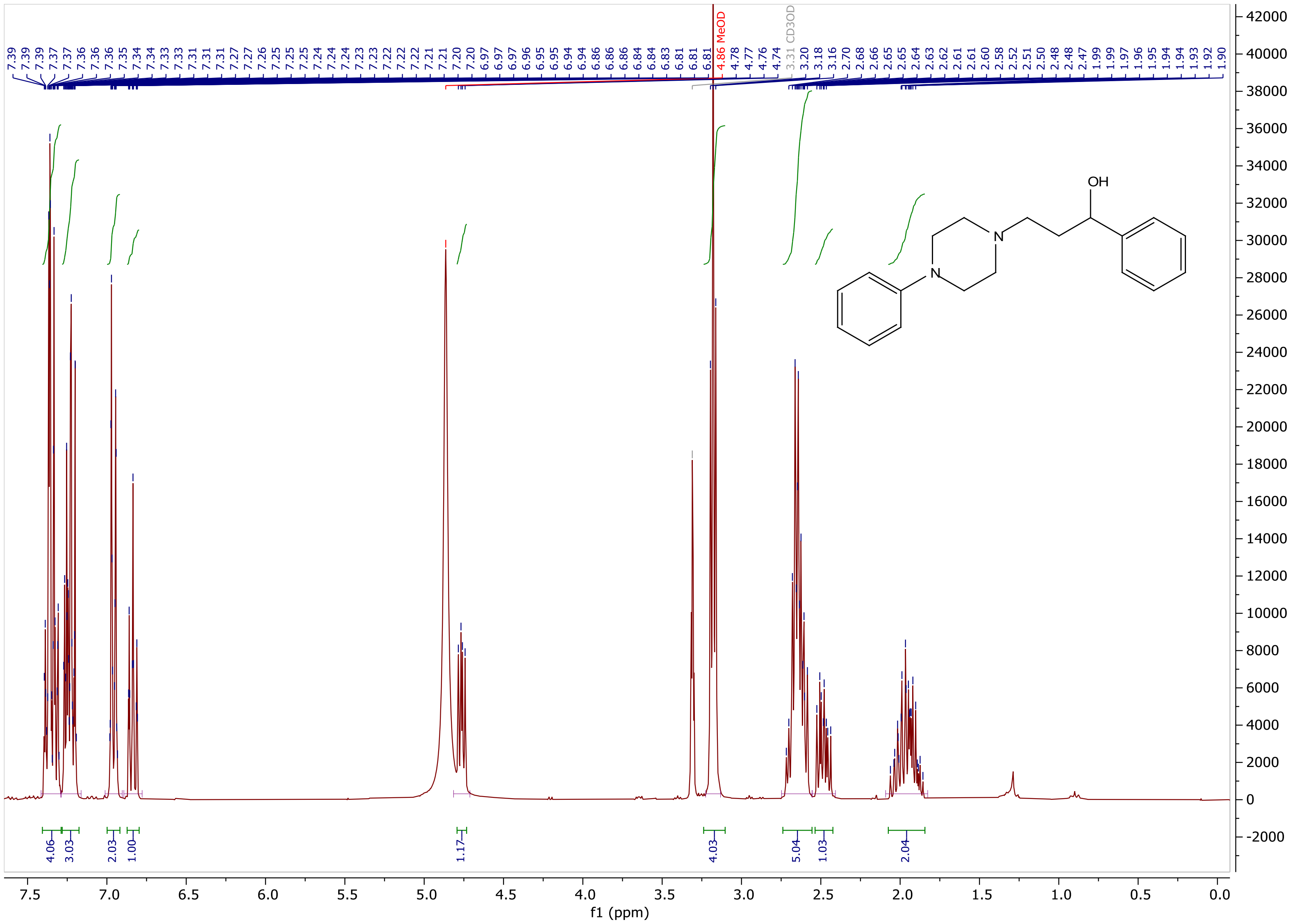

^{1}H NMR (300 MHz, CD_3OD) spectrum of compound **5e**.

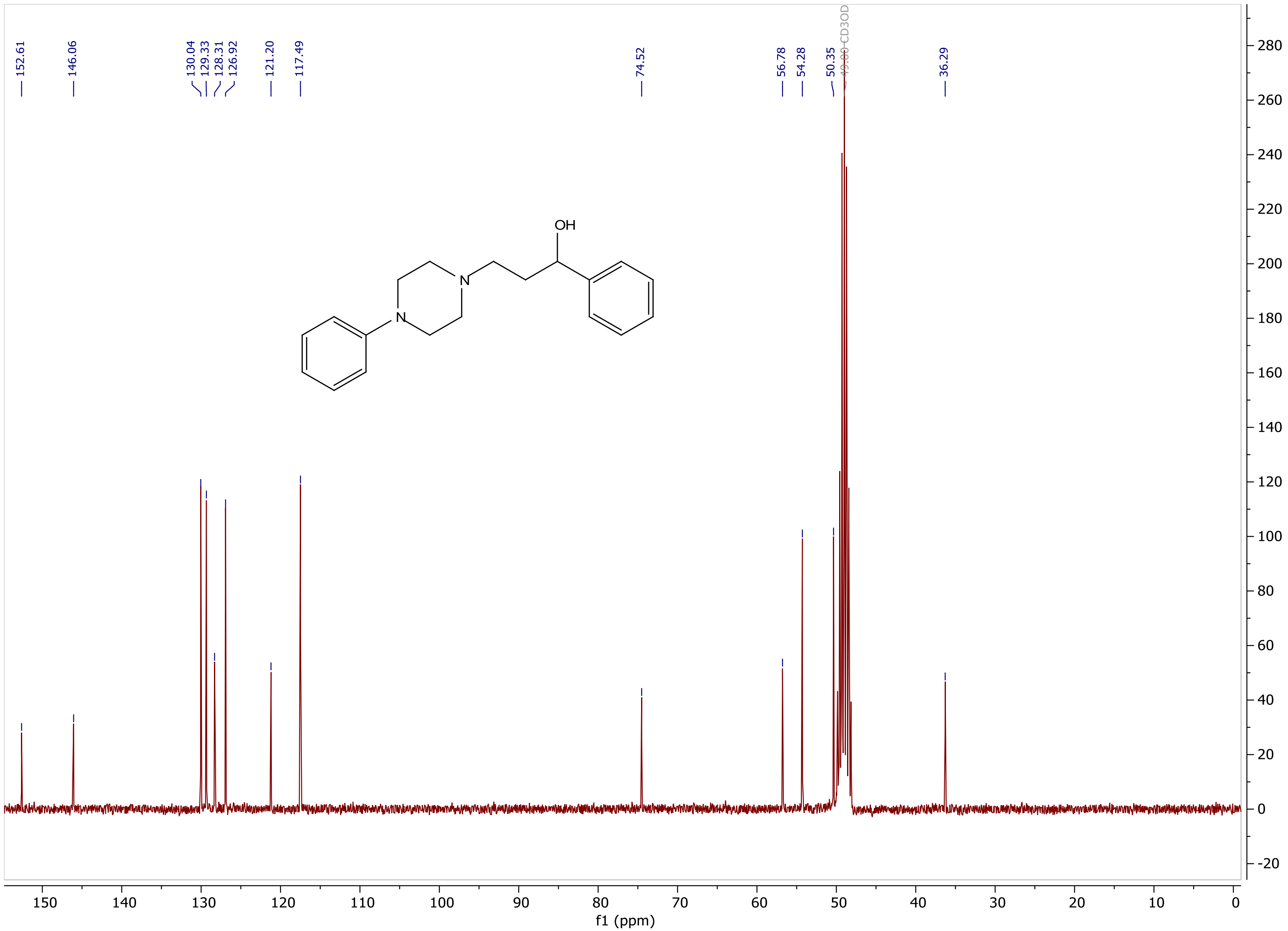

^{13}C NMR (75 MHz, CD_3OD) spectrum of compound **5e**.

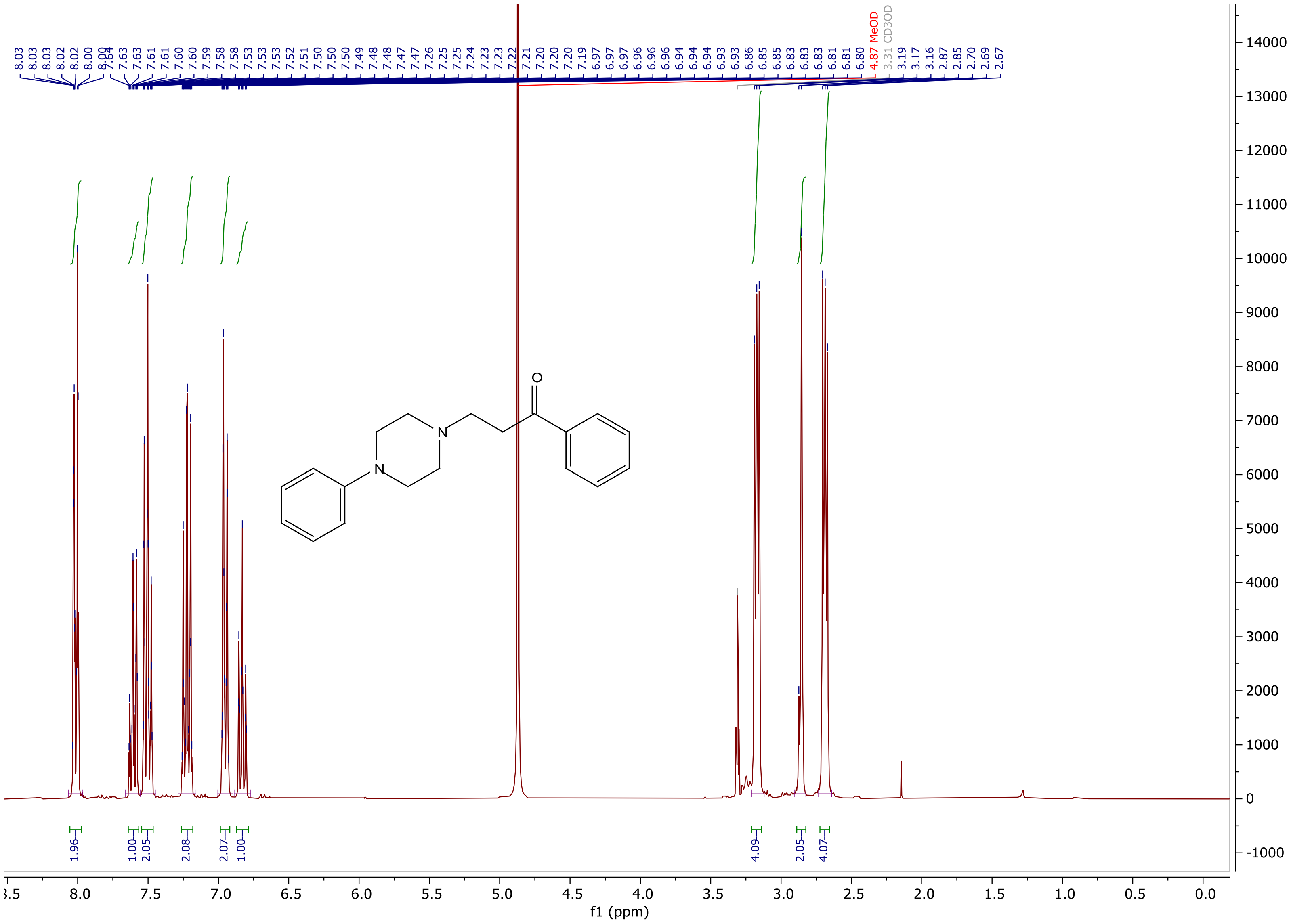

^{1}H NMR (300 MHz, CD_3OD) spectrum of compound **5e`**.

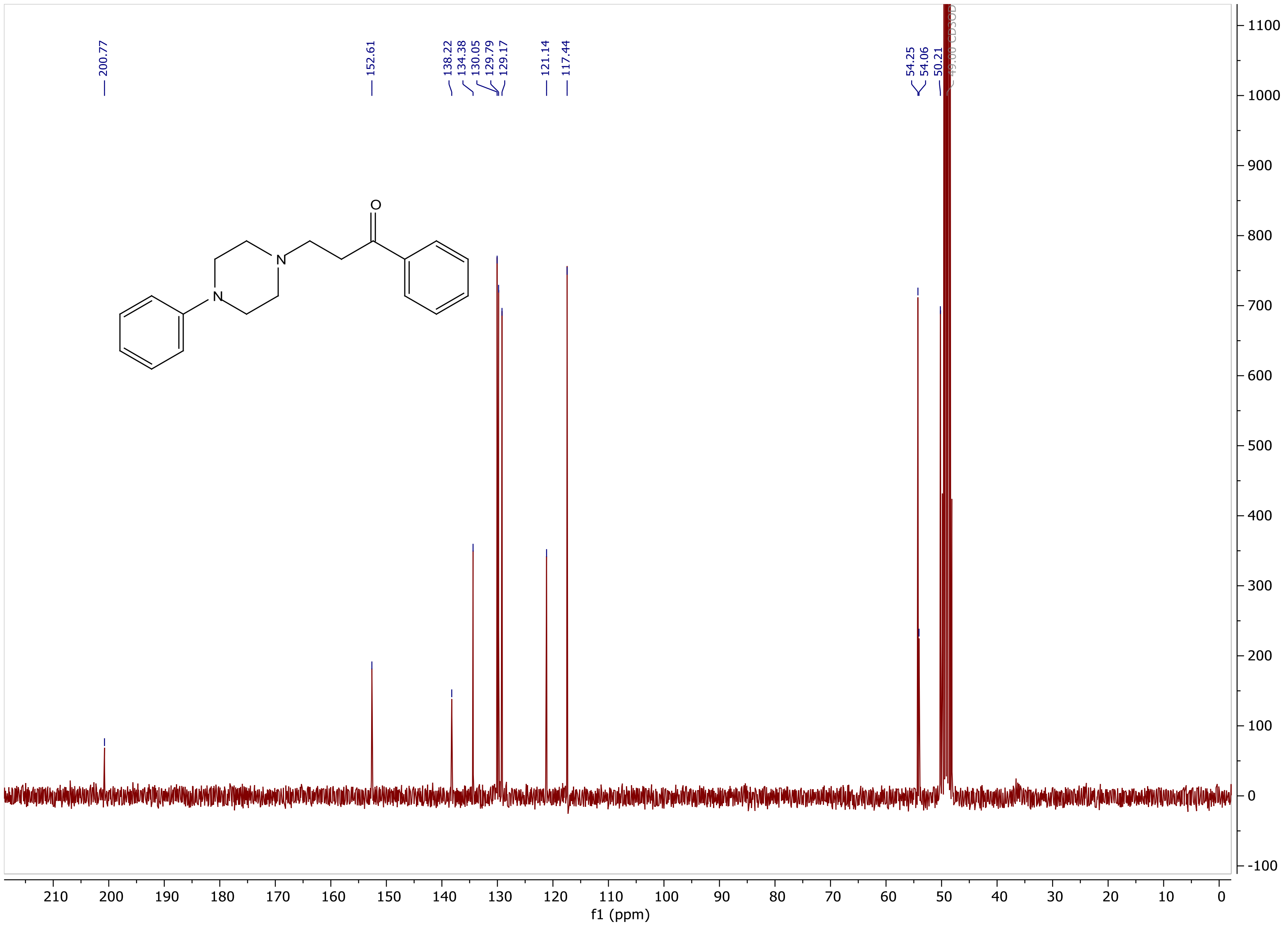

^{13}C NMR (75 MHz, CD_3OD) spectrum of compound **5e`**.

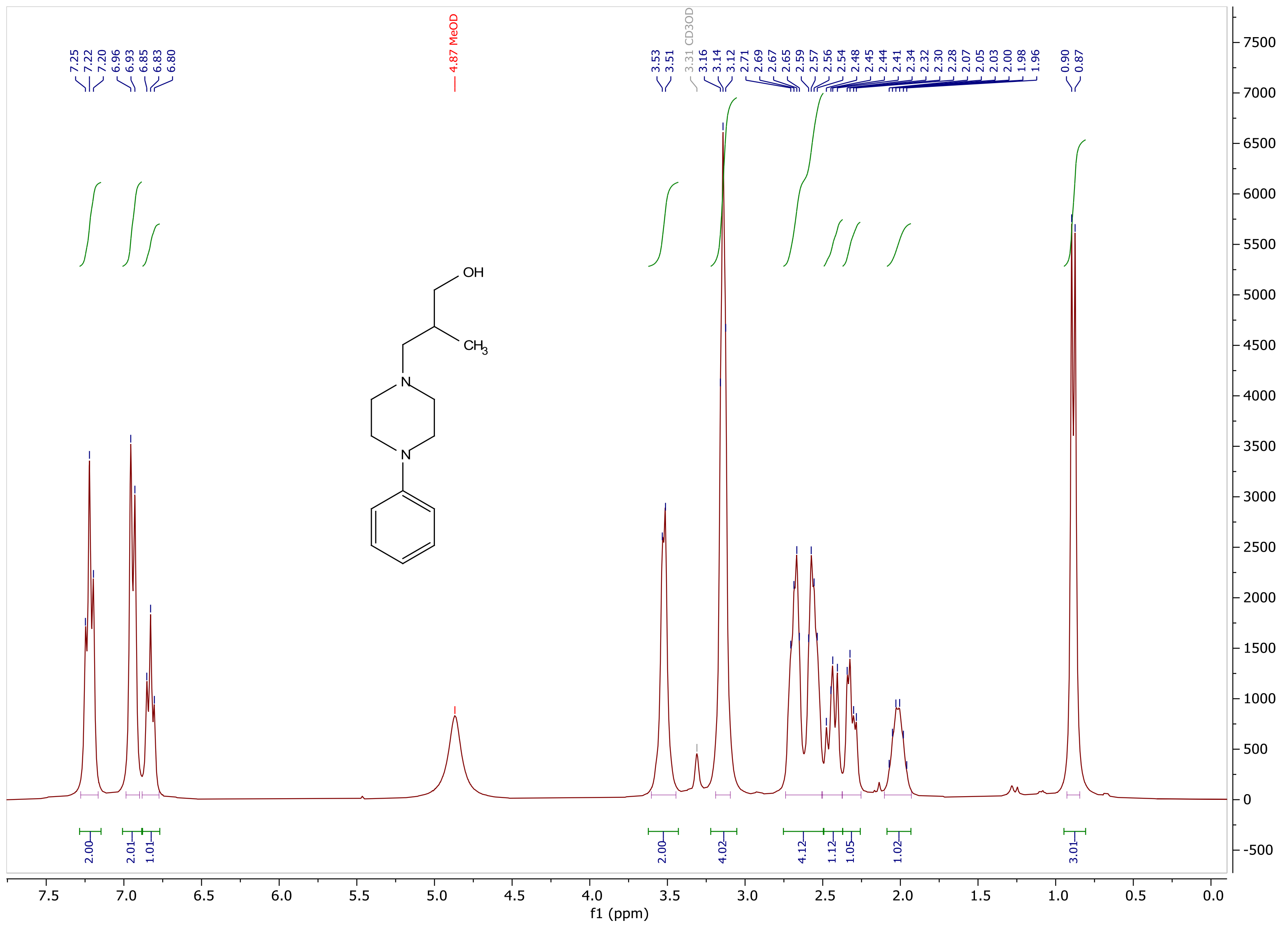

^{1}H NMR (300 MHz, CD_3OD) spectrum of compound **5f**.

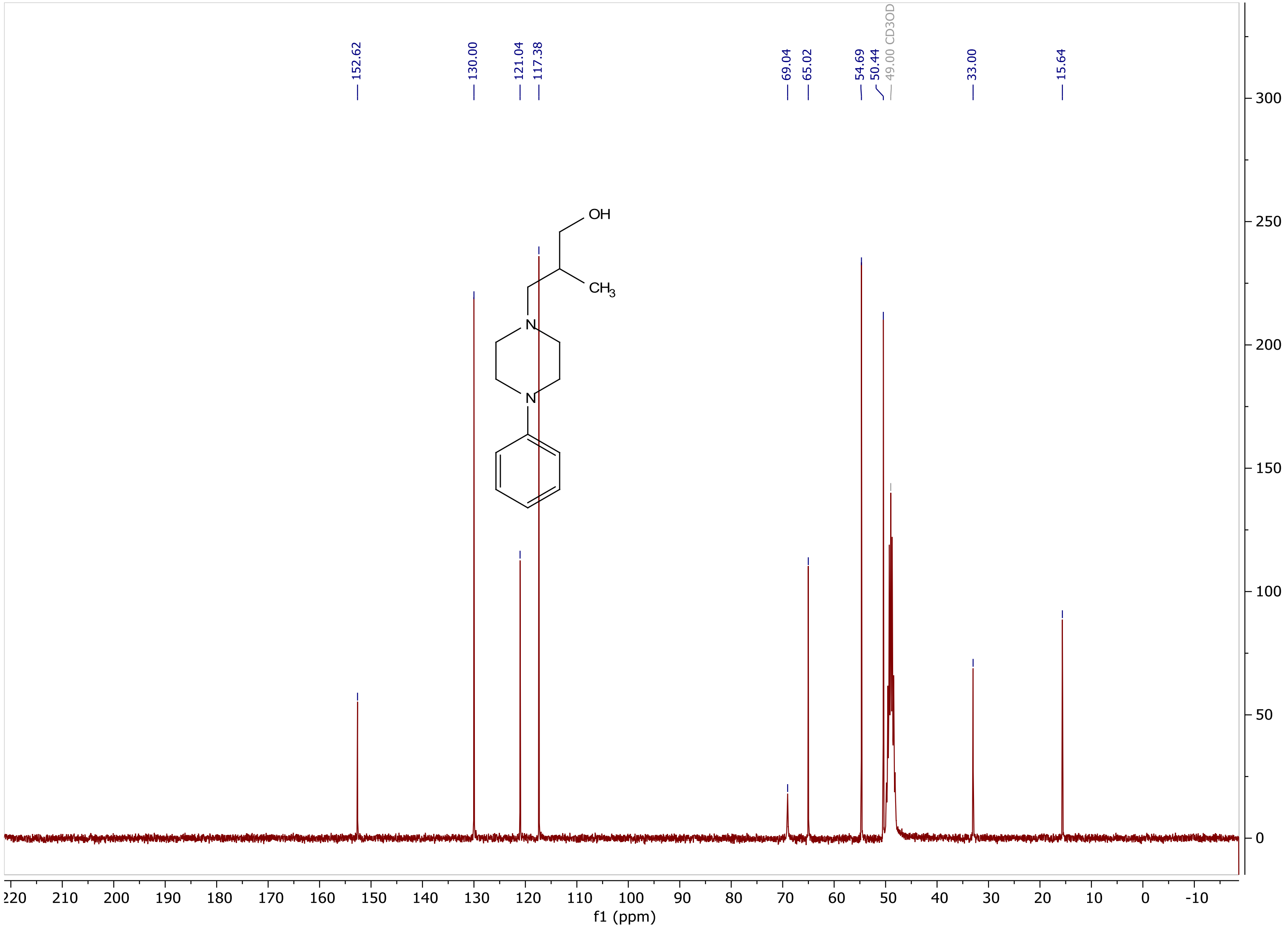

^{13}C NMR (75 MHz, CD_3OD) spectrum of compound **5f**.

7. References

1. M. Garbe, K. Junge, S. Walker, Z. Wei, H. Jiao, A. Spannenberg, S. Bachmann, M. Scalone and M. Beller, *Angew Chem Int Ed Engl*, 2017, **56**, 11237-11241.
2. S. Elangovan, M. Garbe, H. Jiao, A. Spannenberg, K. Junge and M. Beller, *Angew Chem Int Ed Engl*, 2016, **55**, 15364-15368.
3. M. Garbe, S. Budweg, V. Papa, Z. Wei, H. Hornke, S. Bachmann, M. Scalone, A. Spannenberg, H. Jiao, K. Junge and M. Beller, *Catalysis Science & Technology*, 2020, **10**, 3994-4001.
4. J. C. Borghs, Y. Lebedev, M. Rueping and O. El-Sepelgy, *Org Lett*, 2019, **21**, 70-74.
5. M. Anderez-Fernandez, L. K. Vogt, S. Fischer, W. Zhou, H. Jiao, M. Garbe, S. Elangovan, K. Junge, H. Junge, R. Ludwig and M. Beller, *Angew Chem Int Ed Engl*, 2017, **56**, 559-562.
6. S. Budweg, K. Junge and M. Beller, *Chem Commun (Camb)*, 2019, **55**, 14143-14146.
7. E. Alberico, P. Sponholz, C. Cordes, M. Nielsen, H. J. Drexler, W. Baumann, H. Junge and M. Beller, *Angew Chem Int Ed Engl*, 2013, **52**, 14162-14166.
8. W. Ma, S. Cui, H. Sun, W. Tang, D. Xue, C. Li, J. Fan, J. Xiao and C. Wang, *Chemistry*, 2018, **24**, 13118-13123.
